# Time‐Domain Visualization of Electron‐Phonon Coupling in Nanographenes

**DOI:** 10.1002/smtd.202500419

**Published:** 2025-05-19

**Authors:** Rafael Muñoz‐Mármol, Saurav Raj, Mattia Russo, Gianluca Serra, Hao Zhao, Giacomo Bassi, Andrea Lucotti, Francesco Scotognella, Giulio Cerullo, Guglielmo Lanzani, Matteo Tommasini, Margherita Maiuri, Akimitsu Narita, Giuseppe Maria Paternò

**Affiliations:** ^1^ Instituto Universitario de Materiales University of Alicante San Vicente del Raspeig 03690 Spain; ^2^ Department of Physics Politecnico di Milano Milano 20133 Italy; ^3^ Organic Carbon and Nanomaterials Unit Okinawa Institute of Science and Technology Graduate University Okinawa 904‐0495 Japan; ^4^ Department of Chemistry Materials and Chemical Engineering Giulio Natta Politecnico di Milano Milan 20133 Italy; ^5^ Department of Applied Science and Technology Politecnico di Torino Corso Duca degli Abruzzi 24 Torino 10129 Italy; ^6^ Center for Nanoscience and Technology Istituto Italiano di Tecnologia Milano 20134 Italy

**Keywords:** coherent molecular vibrations, collective vibrational modes, full‐quantum calculations, impulsive vibrational spectroscopy, nanographenes, Raman spectroscopy, ultrafast transient absorption

## Abstract

Coherent molecular vibrations determine many molecular properties like intersystem crossing or intramolecular charge transfer, holding potential for developing systems with vibrationally controlled electronic dynamics and reactivity. Research efforts have been focused mainly on localized vibrational modes, leaving collective vibrational modes widely unexplored despite their prominent role in driving molecular dynamics. Besides, the lower intensity associated to collective vibrational modes and their low frequency makes their study a demanding task. In this sense, nanographenes are promising materials that can be synthesized with tailored shapes and sizes—including edge substituents—, offering a great platform for studying collective vibrational modes. Here, femtosecond impulsive vibrational spectroscopy, Raman spectroscopy, and density functional theory calculations are combined to investigate for the first time low‐frequency vibrational motions in two dibenzo[*hi*,*st*]ovalene (DBOV) nanographenes. The systematic study of mesityl‐substituted DBOV (DBOV‐Mes) and its chloro‐functionalized derivative (Cl‐DBOV‐Mes) demonstrates that collective vibrational modes supported by DBOV derivatives can be altered with edge substitution, while optoelectronic properties are preserved. The multidisciplinary approach followed in this work sets the stage for studies on collective vibrational modes in nanographenes and other *π*‐conjugated systems.

## Graphical abstract

Impulsive vibrational spectroscopy, Raman spectroscopy, and density functional theory calculations are gathered together to unveil elusive low‐frequency collective vibrational modes in dibenzo[*hi*,*st*]ovalene nanographenes, enabling the detailed comparison between vibrational modes in derivatives with different peripheral groups. The study demonstrates that collective vibrational modes can be modified without altering the original properties of the nanographene.

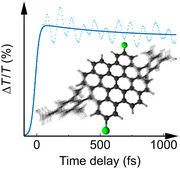

## 1 Introduction

Coherent molecular vibrations and their dephasing determine numerous molecular properties and photochemical reactions. In this sense, selective activation of these vibrations can potentially control electronic dynamics and material reactivity, a holy grail in photochemistry.^[^ ^1^ ^]^ Achieving this daunting task proved difficult, and only a few experimental demonstrations exist today, mostly focused on the modulation of charge transfer (CT),^[^ ^2^, ^3^, ^4^ ^]^ photoisomerization,^[^ ^5^ ^]^ and chemical reaction triggering.^[^ ^6^ ^]^ This scarcity of results is likely due to the nature of the targeted vibrational modes. Typically, research has focused on individual/localized vibrational modes (e.g., CC triple bond stretching) that seem to promote the CT process.^[^ ^2^, ^3^, ^7^ ^]^ However, there is growing evidence linking collective vibrational modes (e.g., breathing modes) with some relevant molecular processes (e.g., singlet fission or thermally activated delayed fluorescence).^[^ ^8^, ^9^ ^]^ Additionally, the relationship between collective vibrational modes and chemical structure—such as the effect of edge substituents—is not well understood. Therefore, mastering these molecular vibrations is crucial for effectively implementing on‐demand photocontrolled properties and reactions.

In this context, nanographenes are promising materials capable of supporting collective vibrational modes that have attracted the attention of the organic optoelectronic community through recent advances in organic synthesis, spectroscopy, and materials science.^[^ ^10^ ^]^ These compounds consist of large polycyclic aromatic hydrocarbons with quasi‐0D graphene structures that confine the electronic wavefunction, opening a finite electronic band gap. Therefore, their optoelectronic properties rely strongly on their size, shape and edge structure, all of them controllable with atomic precision during their synthesis.^[^ ^11^, ^12^, ^13^ ^]^ As a result, nanographenes exhibit a rich excited‐state photophysics and fascinating properties that can be further tuned with edge substitutions,^[^ ^13^ ^]^ making them suitable for different applications ranging from lasing to organic solar cells.^[^ ^11^, ^14^ ^]^ In this context, dibenzo[*hi*,*st*]ovalene (DBOV) derivatives are particularly promising, with demonstrated applications in lasing and strong light‐matter coupling.^[^ ^15^, ^16^ ^]^ They also offer a unique opportunity to investigate the effect of peripheral substitution on their scaffold.^[^ ^17^, ^18^ ^]^ Despite their relevance, data on the collective vibrational modes of DBOV derivatives and other nanographenes remain limited, as the detection of low‐frequency Raman lines*—*i.e., in the order of magnitude of tens to hundreds of cm^−1^—associated to collective vibrational modes is experimentally demanding. Besides, their collective nature results in a lower intensity compared to localized vibrational modes (e.g., CC stretching) making their detection even more challenging.

In this work, femtosecond impulsive vibrational spectroscopy and Raman spectroscopy are implemented synergistically to comprehensively study low‐frequency vibrational motions in mesityl‐substituted DBOV (DBOV‐Mes) and its chloro‐functionalized derivative, Cl‐DBOV‐Mes. Chloro groups were selectively introduced on the apex positions of DBOV‐Mes through a nine‐step synthesis, enabling unambiguous investigation of the effect of the edge functionalization. In this multidisciplinary approach, coherent oscillations present in the temporal dynamics of ultrafast transient absorption (TA) and 2D electronic spectroscopy (2DES) were studied to retrieve information on potential energy surfaces, vibrational frequencies, electron‐phonon coupling, and vibrational dephasing. We identified excited‐state low‐frequency vibrational modes in both DBOV‐Mes and Cl‐DBOV‐Mes, that were assigned to breathing modes through complementary Raman spectroscopy and DFT calculations. Remarkably, while the chloro substitution virtually did not affect the optical properties of DBOV‐Mes, it significantly altered the vibrational landscape, changing the number, position, and electron‐phonon interaction of the collective low‐frequency modes. These results demonstrate that coherent excited‐state collective vibrations can be modulated through edge substitution, making DBOV a promising platform for the development of functionalized derivatives with vibrationally controlled properties—e.g., CT or intersystem crossing. Additionally, the robust multidisciplinary approach demonstrated in this work establishes the foundation for developing methodologies to investigate vibrationally controlled properties.

## 2 Results and Discussion

### 2.1 Synthesis

**Figure 1:**
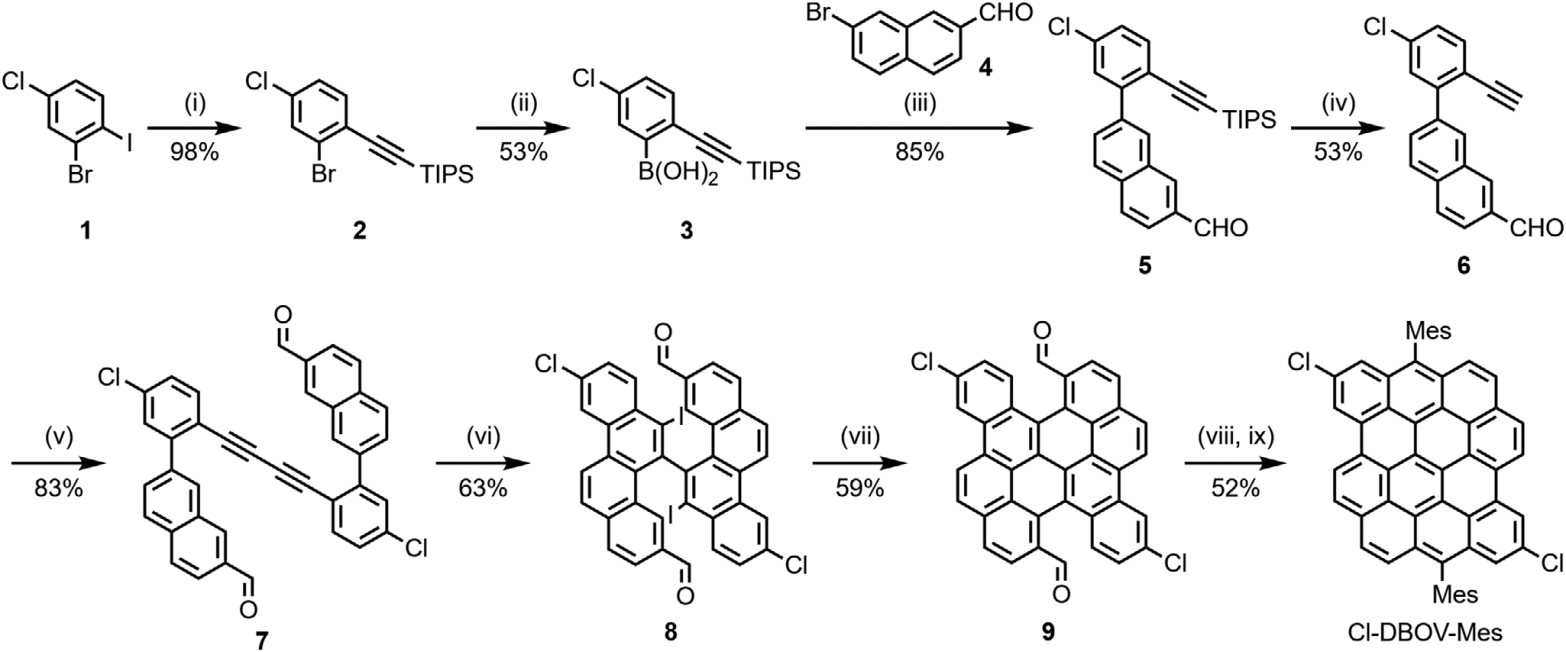
Synthesis of Cl‐DBOV‐Mes. Reagents and conditions: *i*) PdCl_2_(PPh_3_)_2_ (0.05 eq.), CuI (0.1 eq.), TIPS‐acetylene (1.2 eq.), triethylamine, room temperature (rt), 20 h; *ii*) *n*‐BuLi (1.2 eq., 1.6 m in hexane), trimethyl borate (2.0 eq.), tetrahydrofuran (THF), −78 °C, 16 h, *iii*) Pd(PPh_3_)_4_ (0.08 eq.), K_2_CO_3_ (3.0 eq.), toluene/ethanol/H_2_O (4:1:1), 80 °C, 6 h; *iv*) tetrabutylammonium fluoride (1.1 eq., 1.0 m in THF), THF, rt, 2 h; *v*) CuCl (1.0 eq.), dimethylformamide, 80 °C, 10 h; *vi*) ICl (1.0 eq.), dichloromethane, −78 °C, 2 h; *vii*) triethylamine, acetone, *hv*, 2 h; *viii*) 2‐mesitylmagnesium bromide, THF, rt, 3 h; then aq. NH_4_Cl; *ix*) BF_3_•OEt_2_, dichloromethane, rt, 2 h; then *p*‐chloranil, rt, 2 h.

DBOV‐Mes was prepared through our previously reported protocol,^[^ ^16^, ^19^ ^]^ and Cl‐DBOV‐Mes was synthesized in a similar manner as shown in **Figure** **1**. Initially, a Sonogashira coupling of 2‐bromo‐4‐chloro‐1‐iodobenzene (**1**) with triisopropylsilyl (TIPS)‐acetylene gave 2‐bromo‐4‐chloro‐1‐(TIPS‐ethynyl)benzene (**2**) in 98% yield. Borylation of **2** provided boronic acid **3** in 53% yield, which was subjected to a Suzuki coupling with 7‐bromo‐2‐napthaldehyde (**4**) to yield TIPS‐ethynylphenyl‐naphthaldehyde **5** in 85% yield. Subsequent deprotection of **5** yielded ethynylphenyl‐naphthaldehyde **6** in 53% yield, and a Glaser coupling of **6** gave diaryldiacetylene **7** in 83% yield. An iodine monochloride (ICl)‐induced electrophilic iodocyclization^[^ ^20^ ^]^ of **7** yielded diiodobichrysene **8** in 63% yield, which underwent a photochemical cyclodehydroiodination^[^ ^21^, ^22^ ^]^ to afford fused bichrysene **9** in 59% yield, as a red solid. Finally, dialdehyde **9** was subjected to a nucleophilic addition of the mesityl Grignard reagent, followed by Friedel–Crafts cyclization and oxidation,^[^ ^23^, ^24^ ^]^ to obtain Cl‐DBOV‐Mes in 52% yield, as a blue solid.

### 2.2 Steady‐State Optoelectronic Characteristics

**Figure 2:**
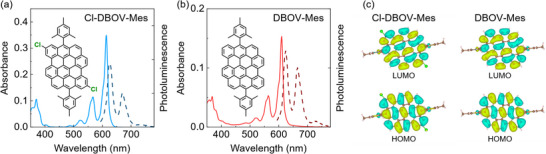
Absorption (solid line) and PL (dashed line) spectra of a) Cl‐DBOV‐Mes and b) DBOV‐Mes in toluene solution (0.1 mg mL^−1^; 1 mm cuvette). Chemical structures are included as insets. c) Frontier molecular orbitals (isosurfaces at 0.01 bohr^−3/2^) of Cl‐DBOV‐Mes and DBOV‐Mes calculated with TD‐DFT. Regions with different colors represent opposite signs of the wavefunctions.

Despite the substitution with chloro groups, the optical properties of Cl‐DBOV‐Mes resemble closely those of DBOV‐Mes. **Figure** **2**a shows the absorption spectrum (solid blue line) and the photoluminescence (PL; dashed dark blue line) spectrum of Cl‐DBOV‐Mes in toluene solution (0.1 mg mL^−1^). The steady state absorption shows two vibronic progressions with principal maxima at 614 and 370 nm that are in good agreement with time‐dependent density functional theory (TD‐DFT) calculations—617 and 368 nm, respectively. The PL spectrum presents a maximum at 617 nm with vibronic replicas at 670 and 734 nm, mirroring absorption. The small Stokes shift (3 nm; 10 meV) and high PL quantum yield (PLQY; 91%) are typical of rigid planar molecular structures. Similar findings were reported for DBOV‐Mes (see Figure 2b),^[^ ^17^ ^]^ which presents 85% PLQY and 3‐nm blue‐shifted absorption and PL spectra compared with Cl‐DBOV‐Mes. Seemingly, the experimental HOMO‐LUMO gap remains unaffected by chloro groups despite its electron withdrawing character. The chloro groups apparently have a minor impact on the stationary optical properties most probably because, according to TD‐DFT calculations, they are situated in nodes of the HOMO and LUMO (see Figure 2b). As shown in Figure S2 (Supporting Information), the relative energy position and the shape of the frontier orbitals of both molecules are very similar, which accounts for the similarity of their UV–vis absorption spectra.

### 2.3 Impulsive Vibrational Spectroscopy

**Figure 3:**
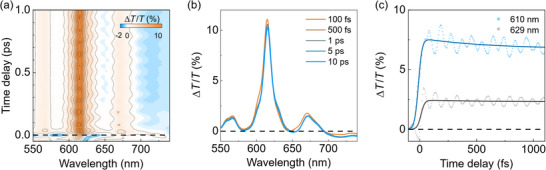
a) Differential transmission (Δ*T*/*T*) map of Cl‐DBOV‐Mes in toluene solution (1 mg mL^−1^; 0.2 mm cuvette) as a function of wavelength and time delay. b) Δ*T*/*T* spectra at various time delays. c) Time traces at 610 (blue squares) and 629 nm (gray circles) probe wavelengths. Full lines are exponential fits to the data. The sample was pumped with 1.2 mJ cm^−2^ (repetition rate = 1 kHz) broad band excitation pulses centered at 600 nm that provided high temporal resolution (15 fs).

Femtosecond TA spectroscopy was used to explore the excited state dynamics of Cl‐DBOV‐Mes, which was dominated by the radiative recombination on the nanosecond timescale. **Figure** **3**a shows the TA map of Cl‐DBOV‐Mes in toluene solution as a function of probe wavelength and delay, measured upon excitation with a 15‐fs pulse at 550–650 nm. The TA spectra, shown in Figure 3b for selected delays, are composed of several features that can be related to three different physical phenomena. In first place, there are positive peaks at 566 and 615 nm matching the ground state absorption spectrum that correspond to ground state bleaching (GSB)—i.e., an increase of the transmitted probe light induced by the ground state population depleted by the pump. The remaining contributions to the TA spectrum correspond to interactions of the excited state population with the probe light, resulting in the promotion of electrons to higher‐lying states (excited state absorption, ESA) or stimulation of radiative decay (stimulated emission, SE), which can be recognized in the TA spectra as the negative band at 718 nm and positive peak at 671 nm, respectively. The SE also contributes to the 615 nm peak. Complementary TA measurements with 70‐fs pulses at 610 nm were used to investigate the dynamics up to 1 ns, showing that all these features decay on the nanosecond timescale (*τ* ≈ 6.2 ns) with a small contribution in the picosecond timescale (*τ* ≈ 4.2 ps); these decays can be ascribed to radiative recombination and conformational rearrangements, respectively (see Figure S3, Supporting Information). The long excited state lifetimes are consistent with the high PLQY, typically related with reduced non‐radiative decay channels. The amplitude of the TA signal scales linearly with the pump fluence, and the shape of the TA spectra is independent of concentration, meaning that there are no saturation or aggregation effects (Figures S3–S5, Supporting Information).

**Figure 4:**
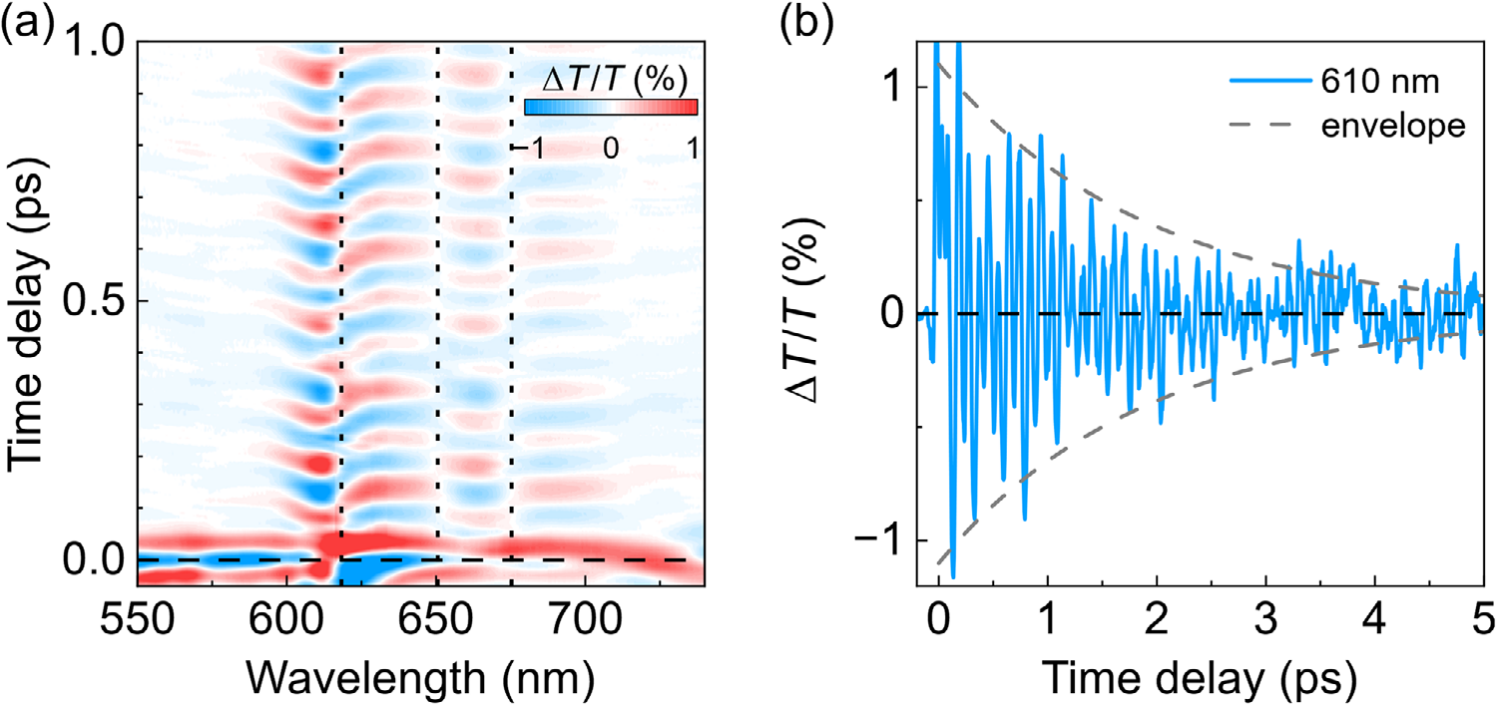
a) Map of the oscillatory component of the Δ*T*/*T* signal for Cl‐DBOV‐Mes in toluene solution (1 mg mL^−1^; 0.2 mm cuvette) obtained after subtracting the slow electronic dynamics. Vertical dotted lines indicate flips of sign in the phase. b) Oscillation dynamics extracted at 610 nm probe wavelength. Dashed gray lines are the exponential envelope function used to calculate the vibrational coherence dephasing time (**≈**1.9 ps).

Figure 3c shows the TA dynamics acquired at 610 and 629 nm up to 1 ps, which display a clear periodic modulation that accounts for impulsively generated vibrational wave packet motion on the ground and excited states.^[^ ^25^ ^]^ These coherent oscillations can be used to study the vibrational landscape of the nanographene following previous studies on other organic materials like oligomers,^[^ ^26^ ^]^ conjugated polymers,^[^ ^27^, ^28^, ^29^ ^]^ or single walled carbon nanotubes.^[^ ^30^, ^31^, ^32^ ^]^ In a typical TA experiment the sample interacts with two ultrashort laser pulses, where the first one acts as a pump and the second one as a probe. When the pump pulse duration is shorter than the molecular vibrational period, coherent vibrational wave packets are generated in the molecular excited and ground states, that follow a classical trajectory of motion on the potential energy surfaces.^[^ ^33^ ^]^ This molecular dynamics modulate the optical response as detected by the probe pulse, providing valuable information about the potential energy surfaces, vibrational frequencies, electron‐phonon coupling, vibrational dephasing, etc.^[^ ^34^, ^35^ ^]^ Such vibrational coherence can be isolated by subtracting the slowly varying incoherent electronic dynamics at each wavelength, leading to the isolated coherent oscillations map shown in **Figure** **4**a. The oscillation pattern is most evident in correspondence with the SE band, suggesting that the vibrational coherence originates mainly from the excited state. Figure 4b shows the oscillatory dynamics at 610 nm, where apparently more than one frequency seems to contribute to the periodic oscillation, which is damped on the picosecond timescale (≈1.9 ps time constant) due to vibrational dephasing.

**Figure 5:**
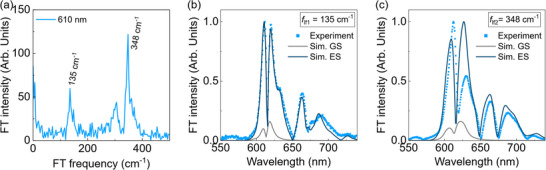
a) Impulsive Raman spectrum for Cl‐DBOV‐Mes in toluene solution (1 mg mL^−1^; 0.2 mm cuvette) at 610 nm and experimental wavelength dependent amplitude of the oscillation for the modes at b) 135 and c) 348 cm^−1^. The corresponding simulated contributions from the ground (gray line) and excited states (blue line) are included for comparison.

Better insight into the impulsively excited vibrational coherences can be gained from the frequency domain analysis. The Fourier transform of the oscillatory signal at each wavelength provides the associated impulsive Raman spectrum, see **Figure** **5**a. In the case of Cl‐DBOV‐Mes, the oscillation consists of two‐low frequency modes at 135 ± 3 and 348 ± 3 cm^−1^. Figure 5b and 5c show the probe wavelength‐dependent amplitude of each of these modes. Both modes show dips at ≈615 and 675 nm, corresponding to the peaks of the PL vibronic replicas. Indeed, the probe wavelength‐dependent phase shows a sign flip occurring at those positions (see Figure S7, Supporting Information) and between both vibronic replicas. These phase flips are also visible in the maps in Figure 4a—vertical dotted lines.

**Figure 6:**
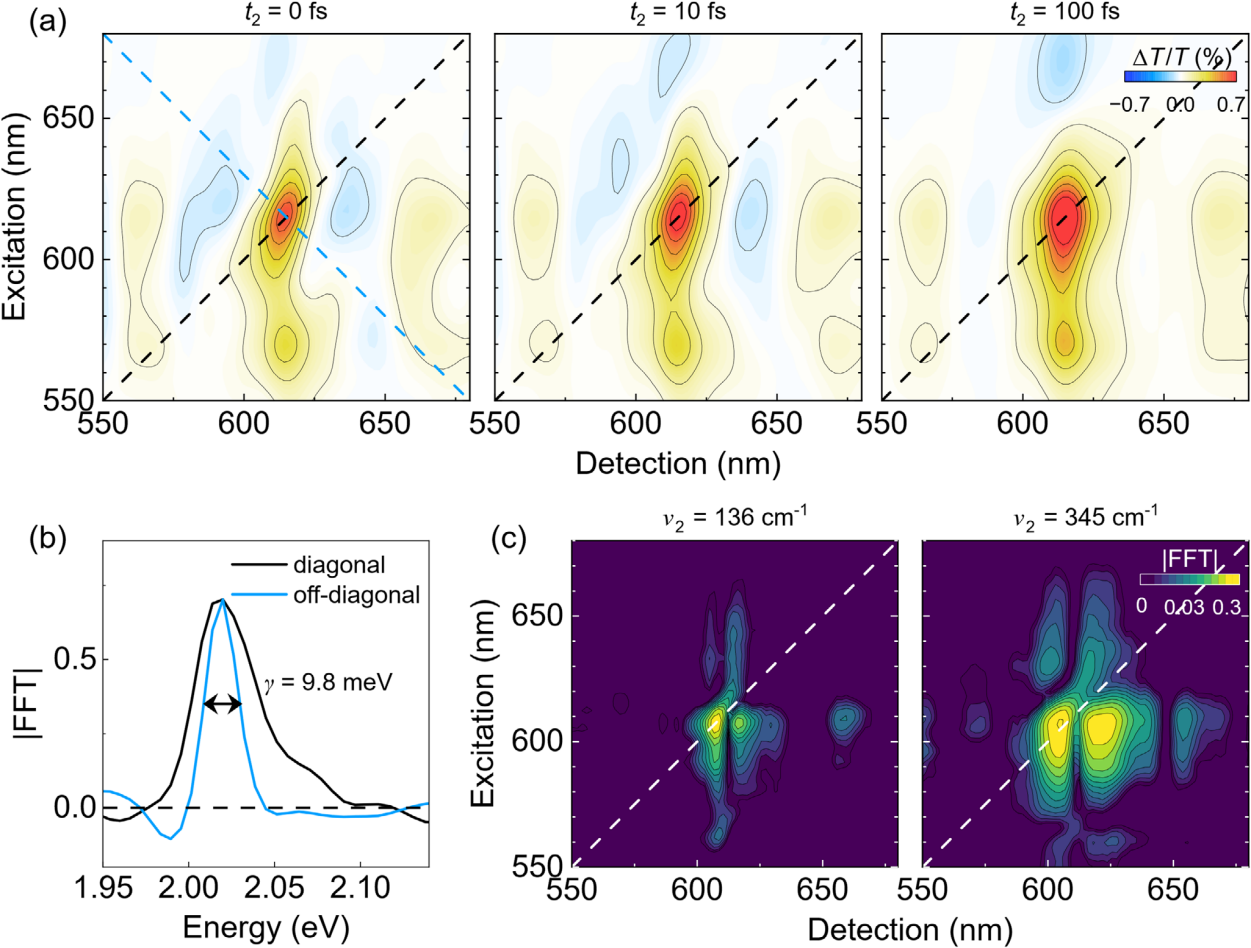
a) 2DES maps at *t*_2_ = 0, 10 and 100 fs for Cl‐DBOV‐Mes in toluene solution (1 mg mL^−1^; 0.2 mm cuvette). b) Diagonal (black) and off‐diagonal (blue) cuts of the 2D map acquired at *t*_2_ = 0 fs. c) 2DES beating map at *ν*_2_ = 136 and 345 cm^−1^.

To develop a model that fully reproduces the experimental data and could provide an explanation for the role of chloro groups in the excited‐state photophysics of DBOV‐Mes, we should characterize the excitation wavelength dependence of the excited state modes and the electronic dephasing time. Although the TA technique provides precious information regarding the time‐domain behaviour, other aspects, such as the excitation energy dependence and the electronic dephasing time of the excited states, are still difficult to measure with a TA experiment. In this perspective, 2DES is perfectly suited for this purpose since it combines high temporal and spectral resolution by providing time‐resolved 2D maps correlating excitation and detection wavelength. In 2DES, three delayed laser pulses interact with system, generating a nonlinear signal: two excitation (pump) pulses, separated by the coherence time *t*_1_, and a detection (probe) pulse, delayed by the waiting time *t*_2_.^[^ ^36^ ^]^ In a 2D map, for a fixed value of *t*_2_, the excitation wavelength is obtained by Fourier transforming the nonlinear signal with respect to *t*_1_, while the detection wavelength is obtained by recording the nonlinear signal with a spectrometer.^[^ ^37^ ^]^ Here we perform 2DES with 15‐fs broadband laser pulses that allow to cover all the GSB and SE peaks observed in the TA measurement. **Figure** **6**a shows 2DES maps for Cl‐DBOV‐Mes at *t*_2_ = 0, 10, and 100 fs. Here, the positive peaks along the diagonal line reflect the GSB signal at 566 and 615 nm, as in the TA spectra. The data also show several cross peaks located at off‐diagonal positions; in particular, by exciting the system at 615 nm, we observe a positive signal at 566 nm (due to the coupling with the vibronic replica of the ground state absorption) and 670 nm (due to the SE signal). Similar behaviour is observed when the system is excited at 566 nm since cross peaks at 615 and 675 nm appear due to GSB/SE and SE, respectively.

The electronic dephasing time is a crucial property of the excited states that can be estimated through the line shape analysis.^[^ ^38^ ^]^ Specifically, 2DES can disentangle homogeneous and inhomogeneous broadening contributions at *t*_2_ = 0 fs by extracting the diagonal and off‐diagonal linewidths of the peaks appearing along the diagonal peak of the 2D map. Figure 6b shows the diagonal (black curve) and anti‐diagonal (blue curve) line shapes of the diagonal peak at 615 nm at *t*_2_ = 0 fs (black and blue dashed lines in Figure 6a). The electronic dephasing rate *γ* = 9.8 meV*, *calculated as the full width at half maximum of the anti‐diagonal profile, corresponds to a dephasing time *T*_2_ = 68 fs.

The high temporal resolution of 2DES allows to capture the oscillations observed in the TA experiment (2DES time trace reported in Figure S9, Supporting Information), giving information on their excitation wavelength dependence. To visualize this contribution, we generate the 2DES beating maps associated with the modes ≈136 ± 7 and 348 ± 7 cm^−1^ by Fourier transforming with respect to the *t*_2_ axis the entire 2DES data set of the coherent part of the signal, while the incoherent part is isolated thorough global fitting and then subtracted from the signal.^[^ ^39^ ^]^ The left panel in Figure 6c shows the 2D beating map at 136 cm^−1^, which represents the amplitude of the mode as a function of the excitation and detection wavelength. In agreement with the TA experiment, the results show the same peak positions at 615 and 675 nm, but here we can observe how the mode couples for different excitation wavelengths—e.g., the mode also appears at cross peak detected ≈615 nm under 566 nm excitation. Besides, the 136 cm^−1^ mode is visible at the SE cross peak (675 nm) for excitation ≈615 nm, but not at 566 nm excitation. This might be indicative of lower coherence under non‐resonant excitation. Indeed, the right panel in Figure 6c shows the 2D beating map at 345 cm^−1^ where the mode appears less localized with respect to the mode at 136 cm^−1^—it is spectrally broader—, but it presents again a lower intensity under 566 nm excitation. Notice that the mode is also slightly visible at the cross peak detected at 566 nm under 615 nm excitation. As a consistency check, we report in Figure S10 (Supporting Information) the 2D beating maps for both modes integrated over the excitation wavelength, which agree with the wavelength dependent oscillatory amplitudes obtained from the TA experiment and reported in Figure 5b,c.

### 2.4 Theoretical Model

An interpretation of the vibrational coherent response of the system can be given in the framework of the time‐dependent wave packet theory for two‐level molecular vibronic system,^[^ ^34^ ^]^ which provided a successful interpretation in the case of conjugated polymers^[^ ^28^, ^29^ ^]^ or single walled carbon nanotubes.^[^ ^40^ ^]^ Typically, the Δ*T*/*T* signal detected in the TA experiment is described by the third‐order susceptibility formalism. However, in the impulsive regime (i.e., for time delays much longer than excitation pulse duration, so that the pulse can be approximated by a Dirac delta), the effective linear response approach can be adopted, in which the nonlinear response is expressed as the linear evolution of a nonstationary wave packet. Thus, the nonstationary state susceptibility is derived from the variation of the density matrix of the system induced by the second‐order interaction with the pump pulse.^[^ ^35^ ^]^ The typical microscopic model is that of a two‐level system with linear electron‐phonon coupling to several vibrational modes. In this model, the same vibrational frequencies *f* are used for the ground and excited states, and only the equilibrium position changes by a dimensionless displacement Δ, which encodes the electron‐phonon coupling. Other important parameters are the energy gap, the phonon population—given by the Bose‐Einstein distribution—and the electronic dephasing time *T*_2_ = 68 fs, that we extract from the 2DES results (Figure 6b). Initially, all the parameters are known except for the displacements. Initial guesses can be retrieved from direct comparison between the simulated and experimental absorption spectrum, see Figure S6 (Supporting Information). Then, the change in the population is calculated using a super‐Gaussian profile that replicates the excitation pulse, the field strength, and dipole moment. Once all those are set, the amplitude and phase of the femtosecond vibrational coherence spectra are calculated. Again, a direct comparison with the experimental spectra is used to finely adjust all the parameters.

In this regard, the simulation with five vibrational modes reproduces in detail the experimental spectra shown in Figure 5, assuming that the coherent vibrational motion happens in the excited state. The two modes with the lowest frequency among the five have the same electron‐phonon coupling—Δ_lf1_ = 0.6 (*f*_lf1_ = 135 cm^−1^) and Δ_lf2_ = 0.6 (*f*_lf2_ = 348 cm^−1^). In both cases, the major contribution to the femtosecond coherence spectra comes from the excited state. The small deviations might be originated from the discrepancy between the experimental pump pulse profile and the super‐Gaussian used in the calculation,^[^ ^40^ ^]^ or additional non‐Franck‐Condon contributions. According to the model, the phase flip and amplitude dip of the excited state contribution happen close to the central position of PL peaks, because the excited state wave packet oscillates around the emitting state equilibrium position, shifted by Δ from the ground state. Besides these two modes, we introduced two other vibrational modes to reproduce the second vibronic peak of the absorption spectrum—*f*_D_ = 1310 cm^−1^ (Δ_D_ = 0.7) and *f*_G_ = 1570 cm^−1^ (Δ_G_ = 0.5)—and an additional mode at low frequency—*f*_Inh_ = 7 cm^−1^ (Δ_Inh_ = 1.5)—accounting for the inhomogeneous broadening. These modes are out of our experimental detection window either because they would require a higher temporal resolution than the one available in our experiments, or because their period is longer than the vibrational coherence dephasing.

The same energy level model is also applied to simulate 2DES data by using a Brownian oscillator model where the Hamiltonian of the system includes electron‐vibrational coupling following the method proposed by Troiani and coworkers.^[^ ^41^ ^]^ The assumptions and the experimental parameters used in the model are detailed elsewhere.^[^ ^42^ ^]^ The Simulated 2DES maps and dynamics are reported in Figure S9 (Supporting Information), showing a good agreement with the experiment.

### 2.5 Raman Characterization

**Figure 7:**
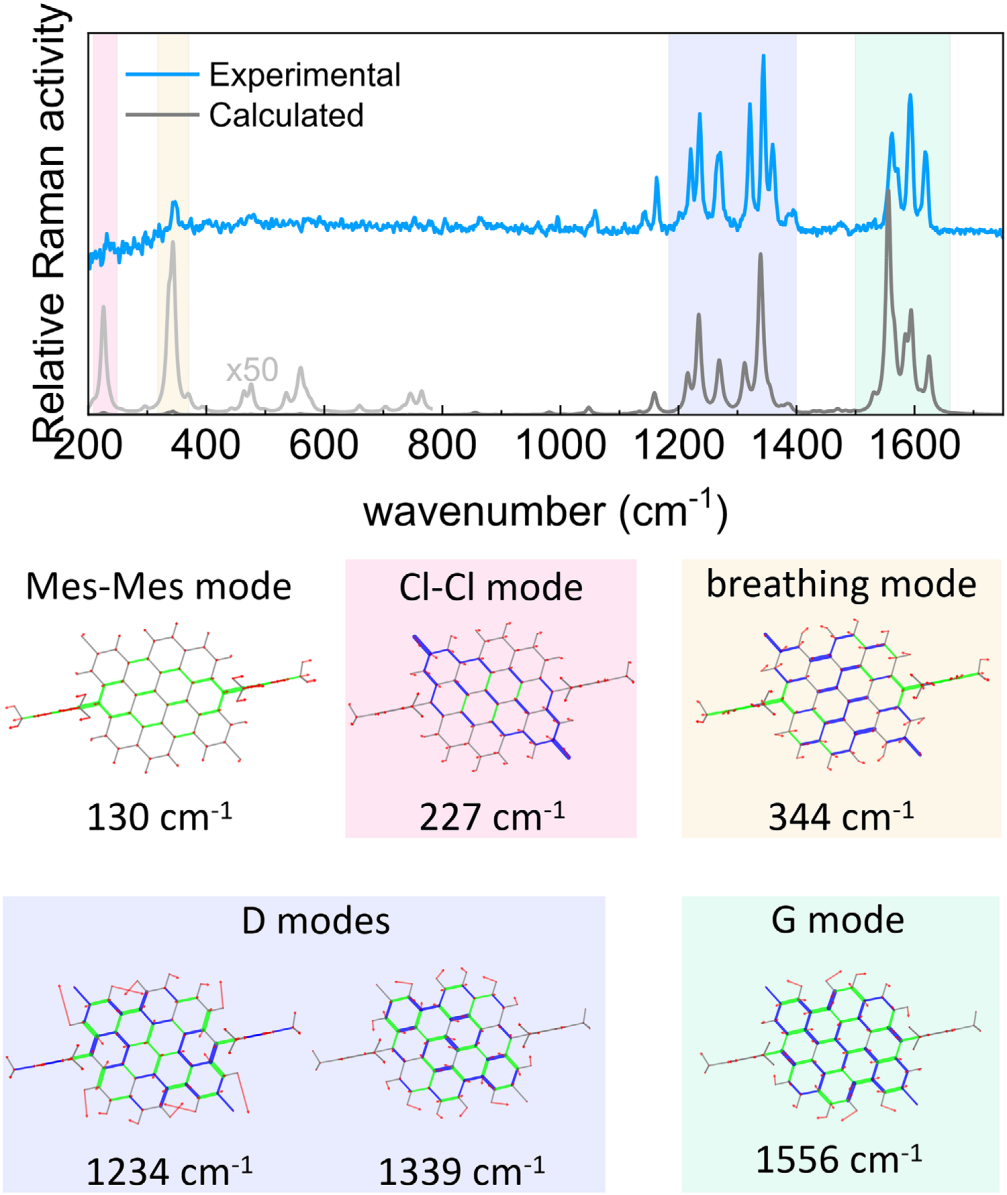
Raman spectrum of Cl‐DBOV‐Mes (blue line, 1064 nm excitation) and calculated Raman spectrum (gray line). Representations of the calculated vibrational normal modes involved in selected Raman‐active transitions are included. Each mode is represented as a cartoon where green and blue segments represent respectively contracting and elongating bonds, and red arrows indicate nuclear displacements. Below each cartoon, the scaled wavenumber of the corresponding mode is reported.

Spontaneous Raman measurements and DFT calculations were performed to reinforce our findings and characterize the vibrational modes. Three sets of vibrational modes contribute to the Raman spectrum in **Figure** **7**. The first set comprises two modes appearing at low frequencies (227 and 344 cm^−1^) that DFT calculations assign to collective vibrational modes along the chloro‐chloro diagonal and full‐core breathing, respectively. The latter mode consists of a breathing mode with a superimposed component along the chloro‐chloro diagonal as shown in the corresponding cartoon in Figure 7. DFT calculations also predict a mode at 130 cm^−1^ assignable to a collective vibration along the mesityl‐mesityl diagonal. The remaining two sets of modes comprise several peaks appearing ≈1270 and 1600 cm^−1^. These are termed D and G modes, respectively, and correspond to in‐plane deformations of the condensed rings. Particularly, the G and D modes correspond to CC stretching and breathing of the benzene rings, respectively.

Some of these modes identified by Raman spectroscopy can be associated with the vibrational modes observed with impulsive vibrational spectroscopy. This is supported by the sizeable electron‐phonon coupling suggested by the TDDFT‐computed geometry difference between the excited and ground states. Indeed, the geometry difference displays significant variations along the CC bonds involved in these normal modes (see Figure S11, Supporting Information). The vibrational modes *f*_lf1_ = 134 cm^−1^ and *f*_lf2_ = 354 cm^−1^ seemingly correspond with the Raman modes at 130 and 344 cm^−1^, respectively. The modes *f*_G_ and *f*_D_ can be assigned to the G and D Raman modes, respectively, predicted by the quantum chemical calculations. These assignments are further supported by the magnitude of the dimensionless displacement parameters obtained from the theoretical model discussed above (Table S1, Supporting Information), which are equivalent to the Huang‐Rhys factors and agree with the results obtained from TDDFT calculations (Table S2, Supporting Information). The small frequency mismatch between the modes found with Raman and impulsive vibrational spectroscopy might be compatible with a variation between the ground and excited potential energy surfaces. Notice that ground‐state vibrational modes are observed with Raman spectroscopy, while impulsive vibrational spectroscopy mainly characterizes excited‐state oscillations. Moreover, non‐Franck‐Condon contributions cannot be ruled out. The data reported in Table S2 (Supporting Information) also shows selected Raman modes for which the Huang‐Rhys factors do not correlate with the Raman activities—e.g., the Raman mode at 227 cm^−1^—; such a correlation is usually observed when the Albrecht's *A* term rules the Raman cross‐section.^[^ ^43^, ^44^ ^]^ The observed miscorrelation is caused by non‐Franck‐Condon contributions to the Raman activities—i.e., *B* and *C* terms in Albrecht's expression of the Raman cross‐section—which involve the expectation value of the electron‐phonon operator between electronic states.^[^ ^43^ ^]^ As a result, the Raman mode at 227 cm^−1^ presents considerable Raman activity and a low Huang‐Rhys factor, which explains its absence in the impulsive vibrational spectrum.

## 3 Discussion

**Figure 8:**
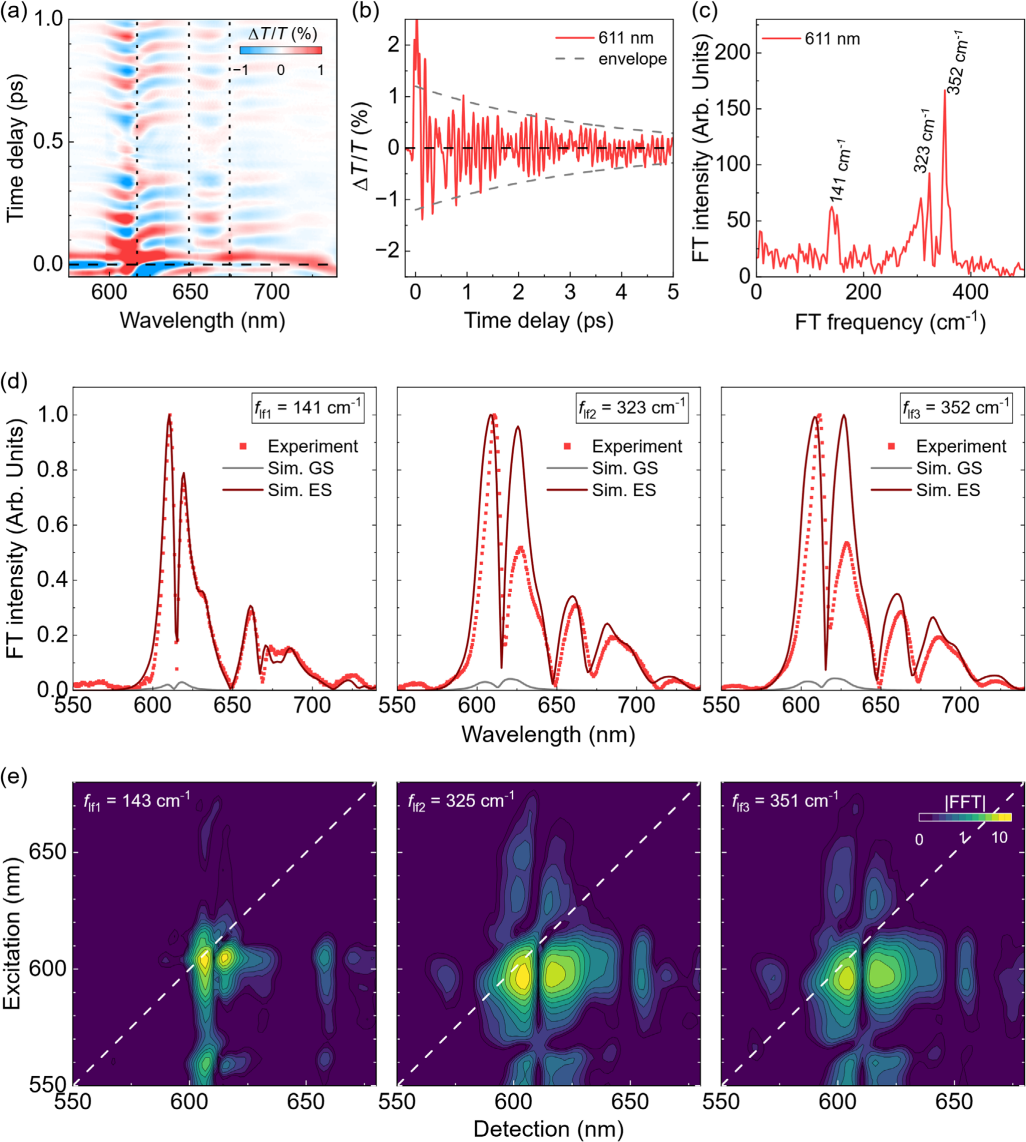
a) Map of the oscillatory component of the TA signal for DBOV‐Mes in toluene solution (1 mg mL^−1^; 0.2 mm cuvette) obtained after subtracting the slow electronic dynamics. Vertical dotted lines indicate flips of sign in the phase. b) Oscillation dynamics at 611 nm. Dashed gray lines are the exponential envelope function used to calculate the vibrational coherence dephasing time (≈3.5 ps). c) Impulsive Raman spectrum at 611 nm and d) experimental amplitude femtosecond coherence spectra for the modes at 141, 323, and 352 cm^−1^. The corresponding simulated contributions from the ground (gray line) and excited states (red line) are included for comparison. e) 2DES beating maps at *ν*_2_ = 143, 325 and 351 cm^−1^.

At this point, it is worth comparing Cl‐DBOV‐Mes with its unfunctionalized counterpart, DBOV‐Mes, to isolate the impact of chloro functionalization on the excited state dynamics. TA data of DBOV‐Mes was reported in a previous work^[^ ^17^ ^]^ showing a similar excited state behaviour to its chloro‐substituted counterpart with almost identical TA spectra and dynamics (see Figure S14–S16, Supporting Information), but the coherent oscillations were disregarded at the time. Indeed, the oscillatory pattern of DBOV‐Mes, reported in **Figure** **8**a, presents some subtle differences with respect to that of Cl‐DBOV‐Mes—e.g., the clear beating pattern in the dynamics or the longer vibrational coherence dephasing time (≈3.5 ps). This suggests that introducing chloro groups enhances the anharmonicity of the vibrational dynamics, reducing the vibrational coherence dephasing time. Further differences appear in the frequency domain. The impulsive Raman spectrum in Figure 8c displays three vibrational modes rather than the two present in Cl‐DBOV‐Mes. There are two modes at 141 and 352 cm^−1^ that spectrally resemble their associated modes found in Cl‐DBOV‐Mes, just shifted to higher energies (6 cm^−1^; 0.5 meV). The mass increase associated with the chloro groups could explain the lower frequencies found in Cl‐DBOV‐Mes. Besides, there is a mode at 323 cm^−1^ resembling the 352 cm^−1^ mode. Seemingly, the 348 cm^−1^ mode of Cl‐DBOV‐Mes has split into two separated modes in DBOV‐Mes, and the small difference in frequency between these two modes is responsible for the beating pattern observed in the oscillation dynamics.

The same difference in the beating pattern is observed in the 2DES data for the DBOV‐Mes, which are reported in Figure S19 (Supporting Information). The 2D maps are very similar to Cl‐DBOV‐Mes in terms of position and incoherent dynamics of the cross peaks. The main difference comes from the frequency analysis of the data where we can still observe, as in the TA experiment, the presence of three modes at 143, 325, and 351 cm^−1^. Figure 8e shows the 2D beating maps associated with these modes, where the low frequency 143 cm^−1^ mode shows a similar distribution to the mode at 136 cm^−1^ in Cl‐DBOV‐Mes, apart from a stronger amplitude in the cross peaks by exciting at 566 nm. The 2D beating maps at 325 and 351 cm^−1^ show a similar excitation/detection wavelength distribution, indicating that they have the same nature. The electronic dephasing time is slightly longer (*Τ*_2_ = 82 fs, extracted from the homogeneous broadening of the 2DES map at *t*_2_ = 0 fs, Figure S21, Supporting Information) than that of the chlorinated DBOV.

Full‐quantum simulations, shown in Figure 8d as solid lines, predict a major contribution to the vibrational coherence from the excited state. Again, some deviations appear between the calculated and experimental modes that might be originated from the discrepancy between the experimental pump pulse profile and the super‐Gaussian used in the calculation, or additional non‐Franck‐Condon contributions. The values found for the displacements of some modes point to a higher electron‐phonon coupling in the chlorinated DBOV (Tables S2 and S3, Supporting Information).

**Figure 9:**
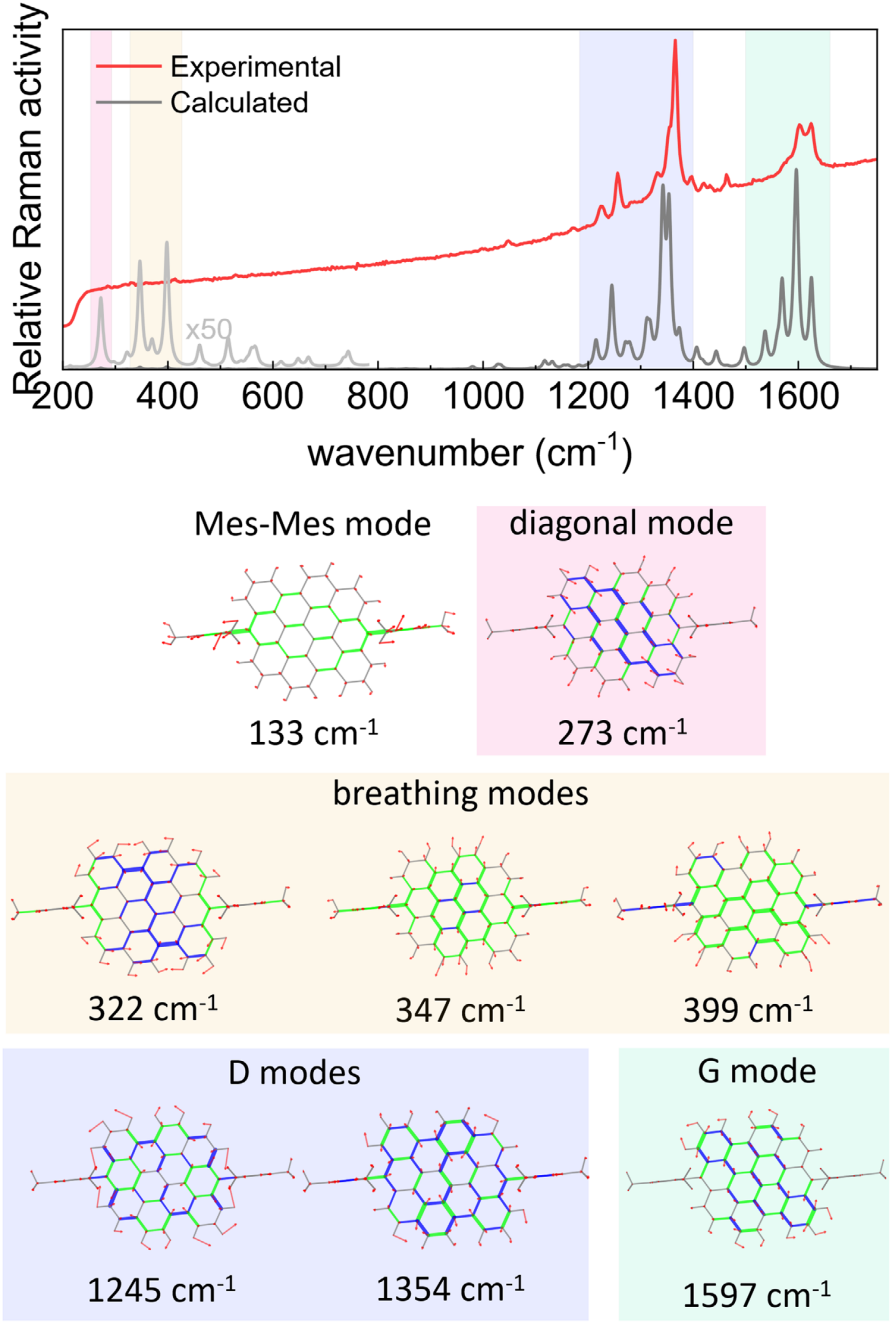
Raman spectrum of DBOV‐Mes (red line, 405 nm excitation) and calculated Raman spectrum (gray line). Representations of the calculated vibrational normal modes involved in selected Raman‐active transitions are included. Each mode is represented as a cartoon where green and blue segments represent respectively contracting and elongating bonds, and red arrows indicate nuclear displacements. Below each cartoon, the scaled wavenumber in cm^−1^ of the corresponding mode is reported.

Interesting conclusions can be extracted from the calculated Raman modes. The Raman spectrum of DBOV‐Mes in **Figure** **9** shows two sets of modes at ≈1280 and 1600 cm^−1^ associated with the D and G modes, respectively, similar to the characteristic structure of Cl‐DBOV‐Mes. Unfortunately, the low‐frequency modes are not observed in the experimental Raman spectrum of DBOV‐Mes due to two factors: *i*) the edge filter suppresses the signal below 220 cm^−1^; and *ii*) the PL background might hide the signal. Nevertheless, DFT calculations corroborate the presence of low‐frequency modes with high Huang‐Rhys factors matching those found with TA and 2DES spectroscopies, even though their computed Raman intensity is much lower than that calculated for the G and D bands. This exemplifies the experimental challenge in detecting collective vibrational modes with Raman spectroscopy. The lower frequency mode computed (133 cm^−1^) corresponds with a collective vibration along the mesityl‐mesityl diagonal of DBOV‐Mes, which is structurally identical to its Cl‐DBOV‐Mes counterpart (130 cm^−1^). It can be assigned to the 141 cm^−1^ mode observed during TA and 2DES experiments. The calculated mode at 273 cm^−1^ consists of a vibration along the apex‐apex diagonal of DBOV‐Mes similar to the chloro‐chloro vibration in Cl‐DBOV‐Mes (227 cm^−1^), just slightly less defined. Then, other three modes are calculated at slightly higher frequencies—347, 398, and 399 cm^−1^—corresponding to full‐core breathing modes. The latter two modes present significant Raman intensity but low Huang‐Rhys factor, explaining their absence in the impulsive vibrational spectrum. On the other hand, the mode at 347 cm^−1^ can be assigned to the 352 cm^−1^ vibrational mode observed with TA and 2DES spectroscopies. The quantum chemical calculations predict another breathing mode at 322 cm^−1^ with low Raman intensity and high Huang‐Rhys factor that can be associated with the vibrational mode observed at 323 cm^−1^. Interestingly, none of these two modes—322 and 347 cm^−1^—fully match the mode calculated at 344 cm^−1^ for Cl‐DBOV‐Mes. Instead, their combined representation resembles the 344 cm^−1^ mode computed for Cl‐DBOV‐Mes, which, despite being just a full‐core breathing mode, also presents coupled Cl‐Cl vibrations that lead to the chloro‐chloro oscillation. Therefore, chloro atoms may play a role in coupling independent breathing modes of DBOV‐Mes into one mode in Cl‐DBOV‐Mes. In general, all the modes calculated for DBOV‐Mes present slightly higher frequencies than their counterparts in Cl‐DBOV‐Mes—following the tendency found with TA and 2DES—because chlorine atoms introduce extra inertia to the vibration. Besides, the vibrational landscape is significantly modified by the chloro substitution, but only some vibrational modes of DBOV‐Mes present significant Huang‐Rhys factors (Table S2, Supporting Information). It is possible to infer an increase of the electron‐phonon coupling associated to the chloro group through the comparison of the Huang‐Rhys factors calculated for vibrational modes with similar nature—i.e., the diagonal modes with negligible Huang‐Rhys factor in DBOV‐Mes have a sizable value in Cl‐DBOV‐Mes, and other vibrational modes like Mes‐Mes and breathing modes have a 25% higher Huang‐Rhys factor in the chloro substituted nanographene. However, such differences in the Huang‐Rhys factors are still in the range that preserves a comparable vibronic progression in the UV‐Vis spectra of both DBOV derivatives.

## 4 Conclusion 

In conclusion, we implemented a multidisciplinary approach that combines femtosecond impulsive vibrational spectroscopy and Raman spectroscopy for studying the electronic and low‐frequency vibrational dynamics in nanographenes and applied it to the case study of DBOV‐Mes and its chloro functionalized derivative Cl‐DBOV‐Mes. We found that the chloro groups modify the vibrational landscape, while preserving the excellent emission properties typical of DBOV‐Mes. Indeed, Cl‐DBOV‐Mes shows high PLQY (91%) and long excited state lifetime dominated by radiative recombination (*τ* ≈ 6.2 ns). The coherent oscillations present at short time delays in the TA dynamics of both derivatives correspond to the time‐evolution of vibrational coherence in the excited state, which gets damped faster in the chlorinated derivative according to its shorter dephasing time (≈ 1.9 ps) compared with DBOV‐Mes (≈3.5 ps). This suggests that the introduction of chloro groups enhances the anharmonicity of the vibrational dynamics, improving thermal relaxation and dissipation. The electronic dephasing time calculated from the 2DES at *t*_2_ = 0 is also shorter in the chloro‐substituted DBOV. Besides, the Fourier analysis revealed that unsubstituted DBOV‐Mes supports three low‐frequency vibrational modes at 141, 323, and 352 cm^−1^, while Cl‐DBOV‐Mes supports just two at 135 and 348 cm^−1^. Raman spectroscopy and DFT calculations were used to assign these modes to collective vibrational modes—mesityl‐mesityl, chloro‐chloro, and full‐core breathing modes—suggesting that the chloro groups are responsible for coupling breathing modes of DBOV‐Mes into a single one in Cl‐DBOV‐Mes. Besides, an increase of the electron‐phonon coupling can be inferred from the comparison of the calculated Huang‐Rhys factors and displacements. Our findings demonstrate that DBOV derivatives support coherent excited‐state collective vibrational modes that can be modified through edge substitution. Such capability is relevant for the development of nanographenes with vibrationally controlled properties. Besides, the approach implemented here proved beneficial for studying simultaneously the dynamics of photophysical processes and the involvement of vibrational modes in them. We are currently working to implement this approach to study charge‐separated states in more complex DBOV derivatives.

## 5 Experimental Methods

### Ultrafast TA (70 and 15 fs)

Ultrafast transient absorption (TA) experiments were performed with a Ti:Sapphire laser (800 nm, 2 kHz, 100 fs duration pulse, 2 mJ). The laser output was split in two used to generate the pump and probe beams. On the one hand, two different pump beams were used for the characterization with a different temporal resolution each: *i*) in the case of 15‐fs TA, broadband pulses—ranging from 550 to 650 nm—were generated with a non‐collinear optical parametric amplifier (NOPA) and compressed to 15‐fs duration using appropriated chirped mirror; *ii*) for 70‐fs TA, a narrowband NOPA was used to amplify a 610 nm signal matching the absorption band of the nanographenes.^[^ ^45^ ^]^ On the other hand, the broadband probe beam was generated by directly focusing the 800‐nm‐beam into a sapphire plate that provides supercontinuum pulses ranging from 400 to 750 nm. Probe's polarization is adjusted with a *λ*/2 waveplate placed before the supercontinuum generation to form a magic angle (54.7°) with respect to the pump's polarization to cancel any signal coming from molecular rotation in the solvent. Then, the pump and probe beams were overlapped on the same spot of the sample (pump diameter was 250 µm), while fluences were adjusted with neutral density filters keeping them low to avoid damaging the sample. The pump‐probe delay was set by means of a mechanical delay stage and pump was chopped to 1 kHz. Finally, the transient transmission spectra were collected by using an optical multichannel analyzer, where the transmittance difference signal (Δ*T*/*T*) was detected.

### 2D Electronic Spectroscopy (2DES)

The partially collinear geometry was adopted,^[^ ^46^, ^47^ ^]^ in which the first two pulses propagate along the same direction and the third pulse propagates along the same direction of the non‐linear signal emitted from the sample. The experimental 2DES apparatus is described in detail elsewhere.^[^ ^48^ ^]^ Briefly, the three pulses were generated by a NOPA,^[^ ^49^ ^]^ pumped by a Ti:Sapphire laser that emits 100 fs laser pulses centred at 800 nm with a 1 kHz repetition rate. The NOPA produces broadband visible pulses with a spectrum spanning from 550 to 700 nm which were compressed down to 15‐fs duration using chirped mirrors. The delay *t*_1_ between the first and the second pulse was controlled with a birefringent interferometer^[^ ^50^ ^]^ and the delay *t*_2_ between the second and the third pulse was controlled by a mechanical delay stage. A single 2D map at a specific delay *t*_2_ was obtained by Fourier transforming the signal acquired as a function of *t*_1_. The experiments were performed using a pump fluence of 100 µJ cm^−2^ with magic angle polarization between pump and probe pulses.

### Full‐Quantum Simulations

Full‐quantum calculations of the coherent phonon spectra were performed with the model proposed by Kumar et al.^[^ ^34^, ^35^ ^]^ The non‐stationary transmission originates from the third order polarizability induced in the sample, but in the impulsive excitation limit—pump‐probe delay *τ* is larger than the pulse duration—the third order susceptibility is formally a linear susceptibility with explicit pump‐probe delay dependence, Δ*χ*(*t*,*τ*). To start with, let us remind the link between linear susceptibility *χ* ^(1)^ and time correlator *K*_g_:

(1) $$\chi^{\left(1\right)}=\frac{i{\left|\mu_{\mathrm{eg}}\right|}^{2}}{\hbar }\;\left[K_{g}\left(t-\tau \right)-K_{g}^{*}\left(t-\tau \right)\right]$$

*µ*_eg_ is the transition dipole moment and ℏ is the reduced Planck constant. Then, Δ*χ*(*t*,*τ*) is in a similar way associated to the time dependent correlator

(2) $$\Delta \chi =\frac{i{\left|\mu_{\mathrm{eg}}\right|}^{2}}{\hbar }\;\left[C_{g}\left(t,\tau \right)-C_{g}^{*}\left(t,\tau \right)\right]+\frac{i{\left|\mu_{\mathrm{eg}}\right|}^{2}}{\hbar }\left[C_{e}\left(t,\tau \right)-C_{e}^{*}\left(t,\tau \right)\right]$$

where g and e refer to ground state and excited state coherence, respectively. For zero vibrational damping

(3) $$C_{u}\;\left(t,\tau \right)=K_{u}\;\left(t-\tau \right)\exp\left(i\omega_{0}A_{u}\Delta \int_{\tau }^{t}ds\;e^{-\gamma \left|s\right|}\times \cos\left(\omega s+\phi_{u}\right)\right)$$

*C*_u_(*t*,*τ*) is the correlator that includes vibrational coherence, showing modulation at the vibrational frequency *ω*. In Equation (2), *γ* is the electronic dephasing, *ω*_0_ is the reduced frequency of the mode, *ϕ*_u_ is an initial phase, and Δ is the dimensionless displacement for the vibrational mode. Notice that Δ = 0 implies no modulation, so only Franck‐Condon active modes can give rise to coherent phonons. $A_{u}=\sqrt{\left(Q_{0u}^{2}+P_{0u}^{2}\right)}\;$ specifies the initial position and momentum. For excited state wave packet *P*_0e_ = 0. Accordingly, the coherent vibrational amplitude in the ground state is ${\bar{Q}}_{g}\;\left(t\right)=\left|A_{g}\right|\;\cos\left(w_{0}t+\phi_{g}\right)$ and ${\bar{Q}}_{e}\;\left(t\right)=\Delta +\left|A_{e}\right|\cos\left(w_{0}t+\phi_{e}\right)$ in the excited state. The initial displacement *Q*_0u_ and momentum *P*_0e_ are obtained by integrals in the frequency space for the overlap between the absorption spectrum and the excitation pulse spectrum. In the original paper by Kumar et al. a very convenient series expansion is then introduced to obtain an analytical solution that can be worked out in the frequency space, thus avoiding cumbersome numerical evaluation in the time domain.

### Raman

The Raman spectrum of DBOV‐Mes was recorded using an HR800 UV dispersive Raman instrument coupled to an Olympus BX41 optical microscope and a Peltier‐cooled CCD. The excitation line was 405 nm. The sample was deposited on brass during the measurement, which was conducted in micro‐Raman mode with a laser spot size of about 1 micron, focusing the laser on the sample using a 50x objective with a numerical aperture of 0.75. The laser power at the sample was about 0.01 mW, and the accumulation time was 60 s (30 averages).

The Raman spectrum of Cl‐DBOV‐Mes was recorded using an FT‐Raman Nicolet NXR 9650 instrument equipped with an Nd‐YVO4 laser excitation at 1064 nm and a Peltier‐cooled InGaAs detector. The powder sample was deposited on a metallic support and analyzed with the micro‐stage FT‐Raman setup (spot size 50 µm, 0.8 W laser power, 4 cm^−1^ spectral resolution, 1024 scans).

### DFT Calculations

For both compounds, B3LYP/6‐31G(d,p) DFT calculations were carried out using the Gaussian program.^[^ ^51^ ^]^ Full geometry optimization was carried out in the gas phase. These molecular structures have been considered for the subsequent calculation of the Raman spectra. To match the different experimental conditions, the Raman polarizability tensors were calculated at 405 nm for DBOV‐Mes and in static (off‐resonance) conditions for Cl‐DBOV‐Mes. To account for the overestimation of the vibrational wavenumbers produced by DFT, the computed wavenumbers were multiplied by 0.97 before plotting the DFT Raman spectra. Time‐dependent B3LYP/6‐31G(d,p) calculations were carried out considering 200 states. Using the same computational setup, the full‐geometry optimization of the first optically allowed excited state with a strong HOMO‐LUMO character was also carried out. The evaluation of the Huang‐Rhys factors has been carried out as described in ref. [52, 53].

## Conflict of Interest

The authors declare no conflict of interest.

## Supporting information



Supporting Information

Supporting Information

## Data Availability

The data that support the findings of this study are openly available in [Zenodo] at [https://doi.org/10.5281/zenodo.14644352], reference number [14644352].
