# Supplementary material for: Time‐Domain Visualization of Electron‐Phonon Coupling in Nanographenes
Source: Small Methods. 2025 May 19;10(2):2500419. doi: 10.1002/smtd.202500419 (PMC12825327; doi:10.1002/smtd.202500419)
Supplement: Supplementary file 1 — Supporting Information [file SMTD-10-2500419-s002.docx]

Supporting Information

Time-Domain Visualization of Electron-Phonon Coupling in Nanographenes

Rafael Muñoz-Mármol,^†^ Saurav Raj,^†^ Mattia Russo,^†^ Gianluca Serra,^†^ Hao Zhao, Giacomo Bassi, Andrea Lucotti, Francesco Scotognella, Giulio Cerullo, Guglielmo Lanzani, Matteo Tommasini,* Margherita Maiuri,* Akimitsu Narita,* Giuseppe Maria Paternò*

*General experimental details*

The standard Schlenk line technique was used for all reactions that are air- and/or moisture-sensitive. All the chemicals were purchased from major chemical suppliers such as TCI, Sigma-Aldrich, and Wako and used as received unless noted otherwise. 7-Bromo-2-naphthaldehyde (**4**) was prepared according to a previously reported procedure.^[1]^ A Glass Contour solvent purification system was used for anhydrous tetrahydrofuran (THF), dichloromethane (DCM), *N*,*N*-dimethylformamide (DMF), and toluene. Thin-layer chromatography (TLC) was done on silica-gel-coated aluminum sheets (particle size 0.063-0.200 nm). Silica gel (230-400 mesh, particle size 0.040-0.060 mm) was used for column chromatography purification. Bruker Avance III Neo 500 MHz and Bruker Avance III Neo 400 MHz Nuclear magnetic resonance (NMR) spectrometers were used to record NMR spectra. Chemical shifts (*δ*) were expressed in parts per million (ppm) relative to the residual solvents (CDCl_3_, ^1^H: 7.26 ppm, ^13^C: 77.16 ppm; THF-*d_8_*, ^1^H: 3.58 and 1.73 ppm, ^13^C: 67.78 and 25.4 ppm). Coupling constants (*J*) were recorded in Hertz (Hz). High-resolution mass spectra (HRMS) were recorded on either Bruker UltrafleXtrem spectrometer by matrix-assisted laser desorption/ionization time-of-flight (MALDI-TOF), using 7,7,8,8-tetracyanoquinodimethane (TCNQ) as the matrix, or on Bruker Compact Quadrupole Time-of-flight (compact QTOF) spectrometer by atmospheric pressure chemical ionization (APCI). UV-Vis absorption and photoluminescence spectra were recorded on Fluorescence+absorbance spectrometer Horiba Duetta using toluene as a solvent in a 1-mm cuvette. Photoluminescence quantum yields (PLQY) were measured using an integrating sphere with a photoluminescence measurement unit (Quantaurus-QY, C11347-01, Hamamatsu Photonics).

**Computational details (DFT, TDDFT):**

We have built full molecular models of **DBOV-Mes** and **Cl-DBOV-Mes** and then performed density functional theory (DFT) and time-dependent DFT (TDDFT) calculations at the B3LYP/6-31G(d,p) level using the Gaussian09^[2]^ software. The geometry of each molecule was fully optimized before calculating any other spectroscopic and electronic properties. For both molecules, 200 excited states were computed by TDDFT.

The equilibrium structure of the lowest bright excited states of **DBOV-Mes** and **Cl-DBOV-Mes** was also computed by geometry optimization at the TDDFT level (Figure S11). The Mulliken charges are routinely reported by Gaussian after a geometry optimization, while the natural bond orbital (NBO) charge distribution analysis was performed on purpose on the optimized geometries of **DBOV-Mes** and **Cl-DBOV-Mes**.

To take into account the overestimation of vibrational wavenumbers produced by DFT, the computed vibrational wavenumbers were multiplied by a shift factor of 0.97 before plotting the simulated Raman spectra reported in the main text. To account for the different excitations used to record the Raman spectra, the Raman spectrum of **DBOV-Mes** was calculated by considering an excitation wavelength of 405 nm, while the calculation of the spectrum of **Cl-DBOV-Mes** was performed in off-resonance conditions. This is a reasonable approximation for the 1064 nm excitation wavelength experimentally adopted.

*Synthesis*

[(2-Bromo-4-chlorophenyl)ethynyl]triisopropylsilane (**2**)^[3]^

A 250-mL round bottom flask was charged with 4-bromo-2-chloro-1-iodobenzene (**1**) (10.0 g, 31.5 mmol), bis(triphenylphosphine)palladium(II)dichloride (Pd(PPh_3_)_2_Cl_2_) (1.11 g, 1.57 mmol), cuprous iodide (CuI) (0.609 g, 3.20 mmol), and triethylamine (100 mL). After degassing by argon bubbling for 30 min, triisopropylsilyl (TIPS)-acetylene (6.76 g, 37.1 mmol) was added dropwise under argon atmosphere. The reaction mixture was stirred at room temperature for 20 h and then quenched with saturated aqueous solution of ammonium chloride (80 mL). The aqueous phase was extracted with two portions of hexane. The organic phases were combined, washed with brine, dried over MgSO_4_, and evaporated. The residue was purified by silica gel column chromatography (eluent: hexane) to give the title compound as yellow oil (11.5 g, 98%). ^1^H NMR (400 MHz, CDCl_3_) *δ* 7.59 (d, *J* = 2.1 Hz, 1H), 7.42 (d, *J* = 8.3 Hz, 1H), 7.23 (dd, *J* = 8.3, 2.1 Hz, 1H), 1.14 (m, 21H). ^13^C NMR (126 MHz, CDCl_3_) *δ* 134.47, 134.31, 132.14, 127.29, 126.17, 124.21, 103.70, 97.45, 18.63, 11.24. HRMS (APCI, Positive): *m*/*z* Calcd. for C_17_H_24_BrClSi^+^: 371.0592 [M+H]^+^, found: 371.0573.

[5-Chloro-2-(triisopropylsilylethynyl)phenyl]boronic acid (**3**)

To a solution of compound **2** (10.0 g, 26.9 mmol) in anhydrous THF (150 mL) was added *n*-butyllithium (*n*-BuLi) (20 mL, 32 mmol, 1.6 M in hexane) dropwise under argon atmosphere at −78 °C. The mixture was stirred at −78 °C for 1 h, and then trimethyl borate (5.59 g, 53.8 mmol) was added to the reaction mixture at −78 °C. The reaction mixture was gradually warmed to room temperature and stirred for 15 h. The reaction was quenched with 2 M HCl aq. (150 mL) and extracted using diethyl ether (three times). The organic phases were combined, washed with brine, dried over MgSO_4,_ and evaporated. The residue was purified by silica gel column chromatography (eluent: hexane: ethyl acetate (EtOAc) = 10:1) to give the title compound as white solid (4.82 g, 53%). ^1^H NMR (400 MHz, CDCl_3_) *δ* 7.95 (d, *J* = 2.3 Hz, 1H), 7.45 (d, *J* = 8.3 Hz, 1H), 7.38 (dd, *J* = 8.3, 2.3 Hz, 1H), 5.93 (s, 2H), 1.15 (m, 21H). ^13^C NMR (126 MHz, CDCl_3_) *δ* 135.91, 135.32, 134.74, 131.24, 125.54, 107.67, 97.38, 18.97, 11.63. HRMS (APCI, Positive): *m*/*z* Calcd. for C_17_H_26_BClO_2_Si^+^: 337.1560 [M+H]^+^, found: 337.1542.

7-[5-Chloro-2-(triisopropylsilylethynyl)phenyl]-2-naphthaldehyde (**5**)

A 500-mL round bottom flask was charged with compound **3** (3.50 g, 10.4 mmol), 7-bromo-2-naphthaldehyde **4** (2.22 g, 9.45 mmol), K_2_CO_3_ (3.92 g, 28.4 mmol), tetrakis(triphenylphosphino)palladium(0) (Pd(PPh_3_)_4_) (0.874 g, 0.756 mmol), and a mixture of toluene, ethanol, and water in the ratio of 4:1:1 (140 mL, 35 mL, and 35 mL) under argon atmosphere. After degassing the reaction mixture by argon bubbling for 30 min, it was refluxed at 80 °C for 6 h. After cooling to room temperature, the resultant mixture was extracted with EtOAc (three times). The organic phases were combined, washed with brine, dried over MgSO_4_, and evaporated. The residue was purified by silica gel column chromatography (eluent: hexane: EtOAc = 20:1) to give the title compound as white solid (3.60 g, 85%). ^1^H NMR (500 MHz, CDCl_3_) *δ* 10.17 (s, 1H), 8.35 (d, *J* = 1.7 Hz, 1H), 8.18 (d, *J* = 1.9 Hz, 1H), 8.00 – 7.91 (m, 3H), 7.85 (dd, *J* = 8.4, 1.8 Hz, 1H), 7.58 (d, *J* = 8.3 Hz, 1H), 7.46 (d, *J* = 2.1 Hz, 1H), 7.33 (dd, *J* = 8.3, 2.2 Hz, 1H), 0.91 (m, 21H). ^13^C NMR (126 MHz, CDCl_3_) *δ* 192.58, 145.21, 138.63, 136.19, 135.38, 135.31, 134.84, 134.76, 132.72, 130.94, 130.15, 129.94, 129.24, 128.22, 128.01, 123.49, 121.20, 105.29, 96.16, 18.85, 11.51. HRMS (APCI, Positive): *m*/*z* Calcd. for C_28_H_31_ClOSi^+^: 447.1905 [M+H]^+^, found: 447.1908.

7-(5-Chloro-2-ethynylphenyl)-2-naphthaldehyde (**6**)

To a solution of compound **5** (8.00 g, 17.9 mmol) in anhydrous THF (240 mL) was added tetra-*n*-butylammonium fluoride (TBAF) (20 mL, 20 mmol, 1.0 M in THF) dropwise under argon atmosphere at 0 °C. The reaction mixture was stirred at room temperature for 2 h. After adding methanol (MeOH) (80 mL) and stirring for another 30 min, the reaction mixture was extracted with EtOAc (three times). The organic phases were combined, washed with brine, dried over MgSO_4_, and evaporated. The residue was purified by silica gel column chromatography (eluent: hexane: EtOAc = 10:1) to give the title compound as white solid (2.76 g, 53%). ^1^H NMR (500 MHz, CDCl_3_) *δ* 10.19 (s, 1H), 8.40 (d, *J* = 1.4 Hz, 1H), 8.19 (d, *J* = 1.7 Hz, 1H), 8.04 – 7.94 (m, 3H), 7.87 (dd, *J* = 8.5, 1.8 Hz, 1H), 7.61 (d, *J* = 8.3 Hz, 1H), 7.49 (d, *J* = 2.2 Hz, 1H), 7.36 (dd, *J* = 8.3, 2.1 Hz, 1H), 3.08 (s, 1H). ^13^C NMR (126 MHz, CDCl_3_) *δ* 192.59, 145.40, 138.19, 136.24, 135.57, 135.51, 135.15, 134.90, 132.79, 130.73, 130.20, 130.14, 129.34, 128.29, 128.15, 123.78, 119.65, 82.27, 81.89. HRMS (APCI, Positive): *m*/*z* Calcd. for C_19_H_11_ClO^+^: 291.0571 [M+H]^+^, found: 291.0559.

1,4-Bis[4-chloro-2-(7-formylnaphthalen-2-yl)phenyl]diacetylene (**7**)

To a solution of compound **6** (1.00 g, 3.44 mmol) in anhydrous DMF (200 mL) was added cuprous chloride (CuCl) (0.340 g, 3.44 mmol). The reaction mixture was refluxed at 80 °C for 10 h under air. After cooling to room temperature, the reaction mixture was diluted with EtOAc (200 mL) and washed with 1 M HCl aq. (100 mL). The aqueous phase was extracted with EtOAc (three times). The organic phases were combined, washed with saturated aqueous solution of sodium bicarbonate (Na_2_CO_3_) and then brine, dried over MgSO_4_, and evaporated. The residue was purified by recrystallization from DCM and MeOH to give the title compound as white solid (0.830 g, 83%). ^1^H NMR (500 MHz, CDCl_3_) *δ* 10.19 (s, 2H), 8.40 (d, *J* = 1.4 Hz, 2H), 8.16 (d, *J* = 1.7 Hz, 2H), 8.03 – 7.97 (m, 6H), 7.84 (dd, *J* = 8.5, 1.8 Hz, 2H), 7.55 (d, *J* = 8.3 Hz, 2H), 7.49 (d, *J* = 2.1 Hz, 2H), 7.34 (dd, *J* = 8.3, 2.2 Hz, 2H). ^13^C NMR (126 MHz, CDCl_3_) *δ* 192.07, 144.87, 137.65, 135.74, 134.80, 134.62, 134.44, 132.39, 130.03, 129.69, 129.48, 128.83, 127.85, 127.70, 123.30, 119.16, 71.88, 67.77. HRMS (APCI, Positive): *m*/*z* Calcd. for C_38_H_20_Cl_2_O_2_^+^: 579.0913 [M+H]^+^, found: 579.0899.

9,9'-Dichloro-3,3'-diformyl-6,6'-diiodo-[5,5'-bichrysene] (**8**)

To a solution of compound **7** (742 mg, 1.28 mmol) in anhydrous DCM (160 mL) was added iodine monochloride (ICl) (1.3 mL, 1.3 mmol, 1.0 M in DCM) dropwise at −78 °C. The reaction mixture was stirred at room temperature for 2 h. The reaction was quenched with saturated aqueous solution of sodium thiosulfate (Na_2_S_2_O_3_) (16 mL) and extracted with DCM. The organic phases were combined, washed with brine, dried over MgSO_4_, and evaporated. The residue was purified by recrystallization from DCM and MeOH to give the title compound as white solid (0.450 g, 42%). ^1^H NMR (400 MHz, CDCl_3_) *δ* 9.02 (d, *J* = 9.2 Hz, 2H), 8.93 (d, *J* = 2.1 Hz, 2H), 8.76 (s, 2H), 8.48 – 8.44 (m, 2H), 8.40 (d, *J* = 8.9 Hz, 2H), 8.29 – 8.20 (m, 2H), 8.02 (d, *J* = 8.3 Hz, 2H), 7.84 (dd, *J* = 8.2, 1.4 Hz, 2H), 7.70 (dd, *J* = 8.9, 2.0 Hz, 2H). ^13^C NMR (126 MHz, CDCl_3_) *δ* 191.34, 149.54, 136.98, 136.58, 135.43, 134.01, 133.10, 132.33, 130.91, 130.45, 130.30, 130.01, 129.61, 129.48, 129.37, 124.79, 123.81, 123.10, 112.57. HRMS (APCI, Positive): *m*/*z* Calcd. for C_38_H_18_Cl_2_I_2_O_2_^+^: 830.8846 [M+H]^+^, found: 830.8835.

2,11-Dichloro-5,14-diformylbenzo[*a*]dinaphtho[2,1,8-cde:1',2',3',4'-ghi]perylene (**9**)

A 600-mL cylindrical quartz reactor was charged with compound **8** (60.0 mg, 0.0723 mmol), acetone (240 mL), and triethylamine (2.4 mL). After degassing by argon bubbling for 30 min, the reaction mixture was irradiated with UV lamps (350 nm, 14 W × 16 lamps) in a photochemical reactor (RPR-200, Southern New England Ultraviolet Company) for 2 h. The solvents were evaporated, and the residue was purified by column chromatography (eluent: hexane/EtOAc: 4:1) to give the title compound (24.5 mg, 59%) as a red solid. ^1^H NMR (400 MHz, CDCl_3_) *δ* 9.39 (s, 2H), 9.04 (d, *J* = 9.3 Hz, 2H), 8.93 (d, *J* = 2.1 Hz, 2H), 8.53 (d, *J* = 8.1 Hz, 2H), 8.50 (d, *J* = 9.0 Hz, 4H), 8.42 (d, *J* = 8.0 , 2H), 8.38 (d, *J* = 9.0 Hz, 2H), 7.58 (dd, *J* = 9.0, 2.0 Hz , 2H). ^13^C NMR (126 MHz, CDCl_3_) *δ* 190.49, 133.84, 133.49, 132.05, 130.25, 129.78, 129.26, 128.74, 128.68, 127.57, 127.48, 126.47, 125.03, 124.81, 123.85, 123.72, 123.04, 121.04, 120.94. HRMS (MALDI-TOF, Positive): *m*/*z* Calcd. for C_38_H_16_Cl_2_O_2_^+^: 574.0522 [M]^+^, found: 574.0547.

4,12-Dichloro-6,14-dimesityldibenzo[*hi,st*]ovalene (**Cl-DBOV-Mes**)

To a solution of compound **9** (10.0 mg, 17.4 μmol) in dry THF (5 mL) was added 2-mesityl magnesium bromide (0.35 mL, 0.35 mmol, 1.0 M in diethyl ether) dropwise at 0 °C. The reaction mixture was stirred for 2 h at room temperature. After quenching with saturated aqueous solution of ammonium chloride (NH_4_Cl) (8 mL), the mixture was extracted with EtOAc (10 mL) three times. The organic phases were combined, washed with brine, dried over MgSO_4_, and evaporated. The obtained diol intermediate was dried and then dissolved in anhydrous DCM (10 mL). After degassing by argon bubbling for 30 min, boron trifluoride etherate (BF_3_∙OEt_2_) (0.1 mL, 0.8 mmol) was added, and the reaction was stirred for 2 h at room temperature. MeOH (0.50 mL), and then *p*-chloranil (4.3 mg, 17 μmol) were added to the reaction mixture, which was stirred for another 2 h at room temperature. The solvents were evaporated, and the residue was purified by column chromatography (eluent: hexane: DCM = 3:1) to give the title compound as a blue-solid (7.0 mg, 52%). ^1^H NMR (500 MHz, THF-*d_8_*:CS_2_ = 1:1) *δ* 9.55 (d, *J* = 8.3 Hz, 2H), 9.17 (d, *J* = 1.9 Hz, 2H), 8.63 (d, *J* = 8.2 Hz, 2H), 8.17 (d, *J* = 9.2 Hz, 2H), 7.82 (d, *J* = 1.8 Hz, 2H), 7.74 (d, *J* = 9.1 Hz, 2H), 7.23 (s, 4H), 2.54 (s, 6H), 1.94 (s, 12H). ^13^C NMR (126 MHz, THF-*d_8_*:CS_2_ = 1:1) *δ* 137.89, 137.50, 134.76, 134.24, 134.02, 133.99, 132.25, 131.50, 129.96, 129.36, 129.00, 128.69, 126.60, 126.38, 124.55, 124.13, 123.72, 123.50, 123.43, 123.11, 122.99, 122.36, 121.45, 30.01, 29.96. HRMS (MALDI-TOF, Positive): *m*/*z* Calcd. for C_56_H_35_Cl_2_^+^: 776.2032 [M]^+^, found: 776.2035.

**Supporting Figures and Tables**


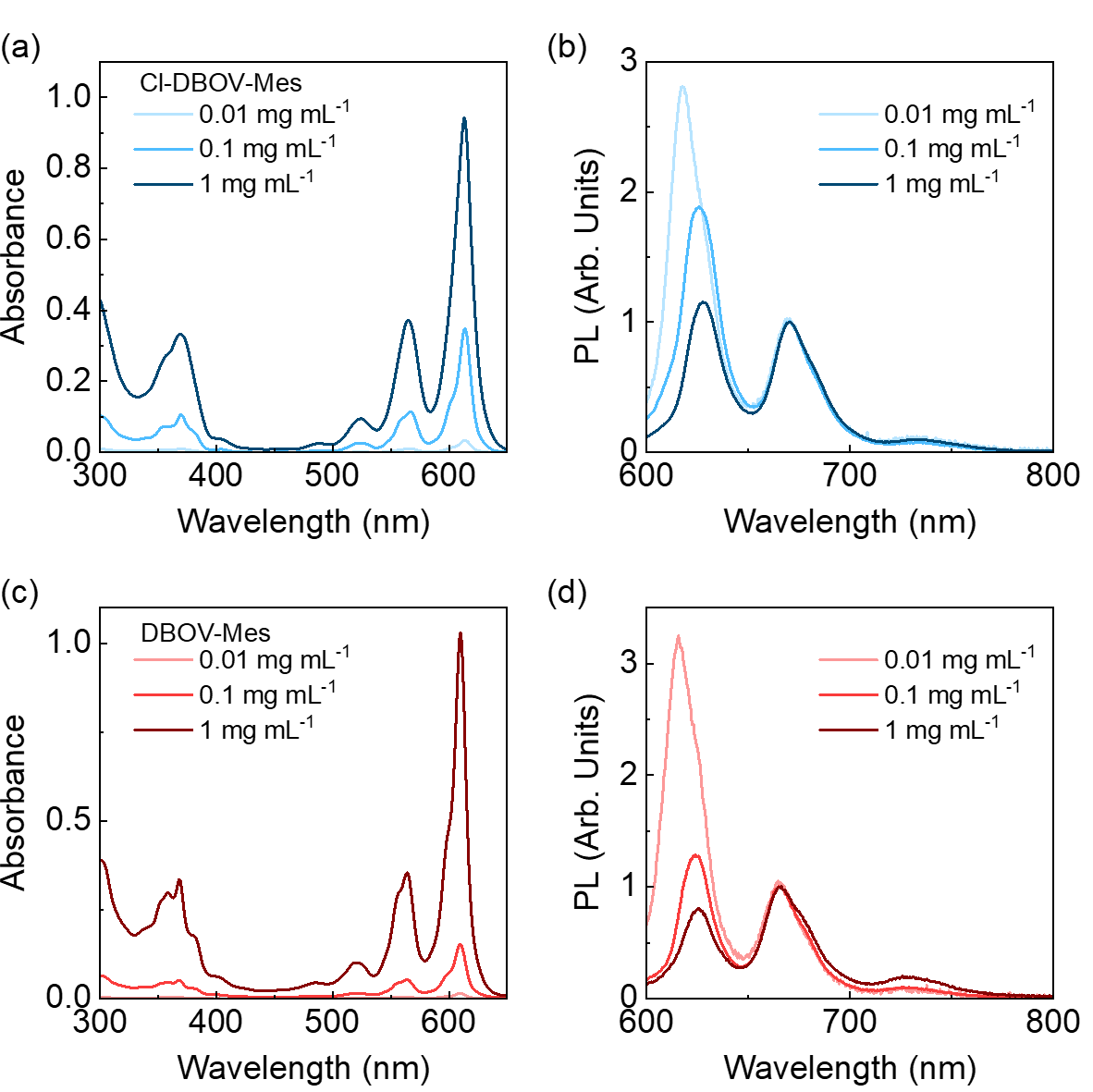


**Figure S1.** Absorption and PL spectra of Cl-DBOV-Mes (a,b) and DBOV-Mes (c,d) in toluene solution (1, 0.1 and 0.01 mg mL^-1^). PL spectra have been normalized to their corresponding second vibronic maxima. The measurements were performed with 1 mm cuvettes.

**Figure S2.** Representation of the molecular orbitals most involved in the two low-lying optical absorption of DBOV-Mes and Cl-DBOV-Mes (after B3LYP/6-31G(d,p) DFT calculations). The lowest bright state (Cl-DBOV-Mes: 617 nm; DBOV-Mes: 615 nm) is assigned by TD-DFT to a transition with strong HOMO-LUMO character and the second bright state (Cl-DBOV-Mes: 367 nm; DBOV-Mes: 366 nm) is assigned to the HOMO-2-LUMO transition coupled with HOMO-LUMO+2. The energy of the orbitals (eV) is also reported relative to the energy of the HOMO orbital which was selected as reference. The energy difference between the HOMOs of DBOV-Mes and Cl-DBOV-Mes is 0.28 eV, with the HOMO of Cl-DBOV-Mes being lower in energy than DBOV-Mes.


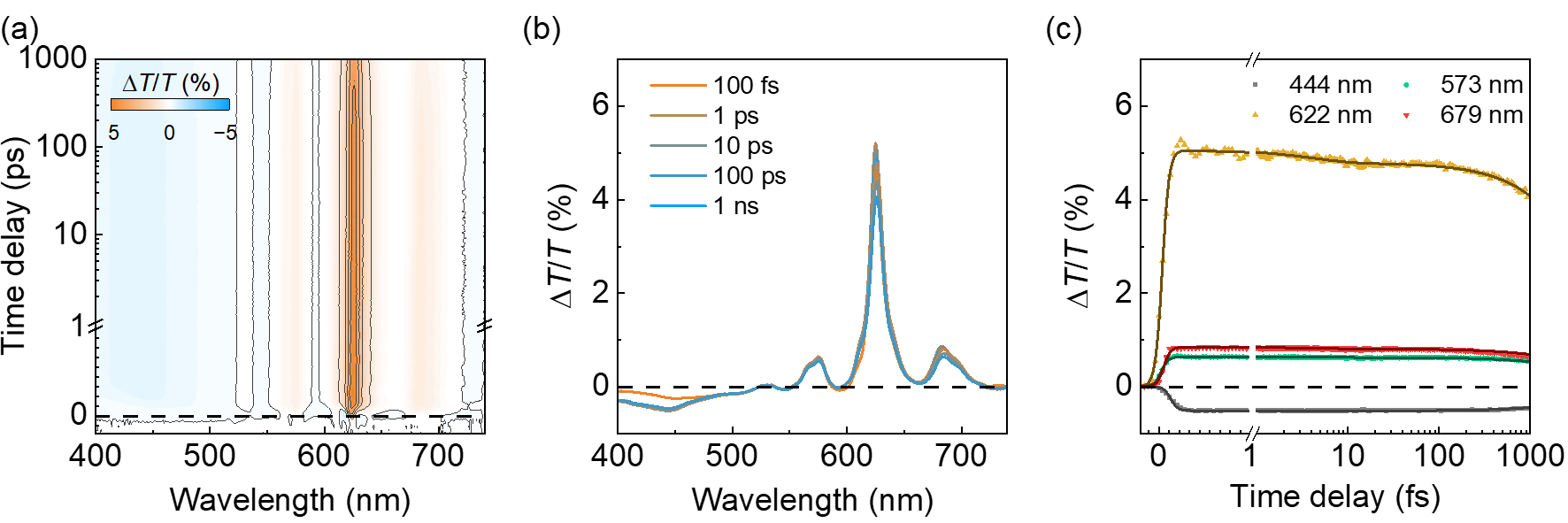


**Figure S3.** (a) Differential transmission (Δ*T*/*T*) map of Cl-DBOV-Mes in toluene solution (0.1 mg mL^-1^; 1 mm cuvette) as a function of wavelength and time delay. (b) Δ*T*/*T* spectra at various time delays. (c) Time traces at 444 (grey squares; ESA), 573 (green circles; GSB), 622 (orange up triangles; GSB+SE) and 679 nm (red down triangles; SE) probe wavelengths. Full lines are exponential fits to the data. The sample was pump at *λ*_pump_ = 610 nm with a fluence *F*_pump_ = 41 µJ cm^-2^ (repetition rate = 1 kHz; Δ*t*_pump_ = 70 fs).


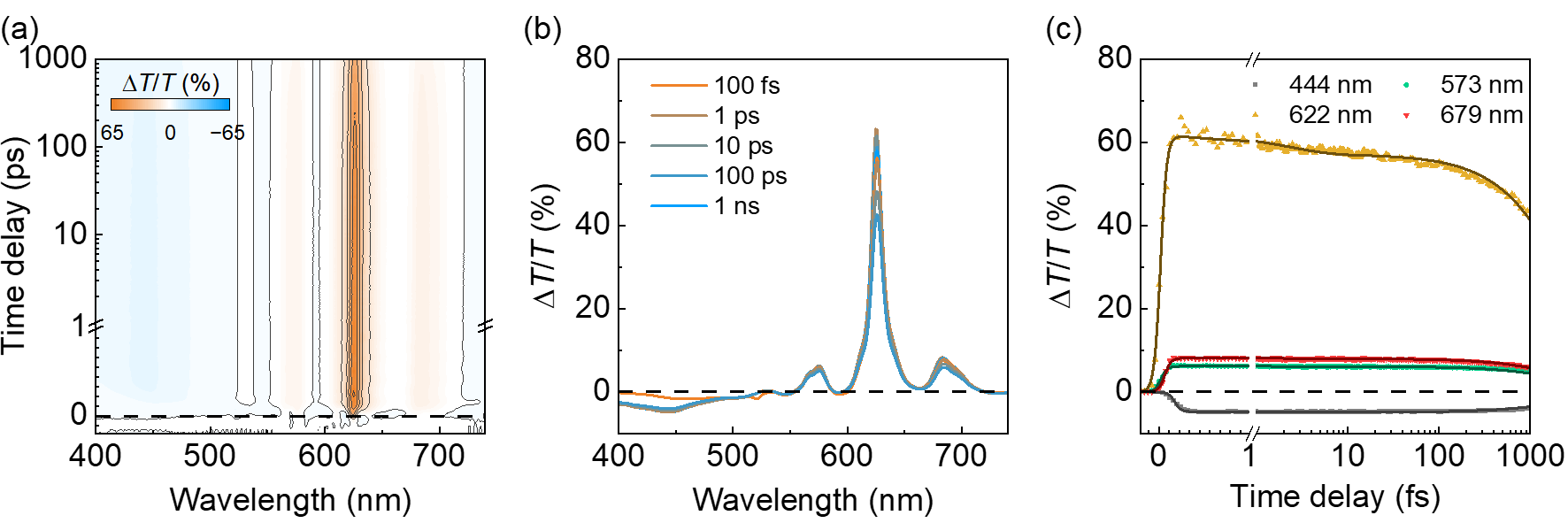


**Figure S4.** (a) Differential transmission (Δ*T*/*T*) map of Cl-DBOV-Mes in toluene solution (0.1 mg mL^-1^; 1 mm cuvette) as a function of wavelength and time delay. (b) Δ*T*/*T* spectra at various time delays. (c) Time traces at 444 (grey squares; ESA), 573 (green circles; GSB), 622 (orange up triangles; GSB+SE) and 679 nm (red down triangles; SE) probe wavelengths. Full lines are exponential fits to the data. The sample was pump at *λ*_pump_ = 610 nm with a fluence *F*_pump_ = 410 µJ cm^-2^ (repetition rate = 1 kHz; Δ*t*_pump_ = 70 fs).


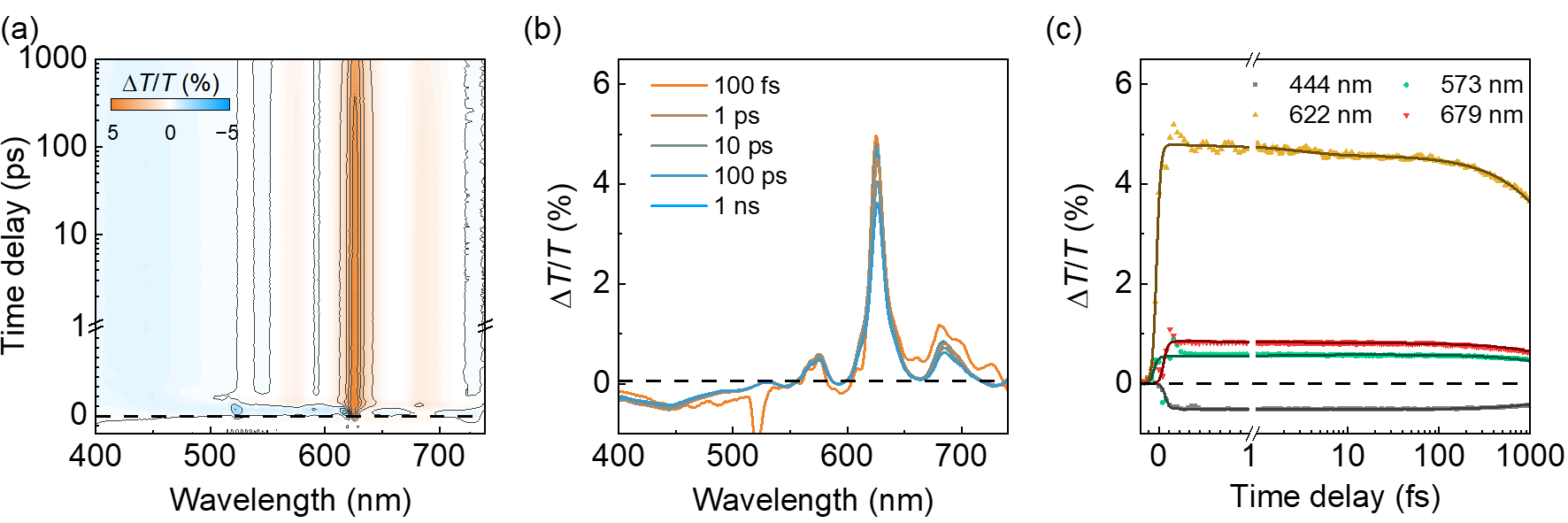


**Figure S5.** (a) Differential transmission (Δ*T*/*T*) map of Cl-DBOV-Mes in toluene solution (0.01 mg mL^-1^; 1 mm cuvette) as a function of wavelength and time delay. (b) Δ*T*/*T* spectra at various time delays. (c) Time traces at 444 (grey squares; ESA), 573 (green circles; GSB), 622 (orange up triangles; GSB+SE) and 679 nm (red down triangles; SE) probe wavelengths. Full lines are exponential fits to the data. The sample was pump at *λ*_pump_ = 610 nm with a fluence *F*_pump_ = 410 µJ cm^-2^ (repetition rate = 1 kHz; Δ*t*_pump_ = 70 fs).


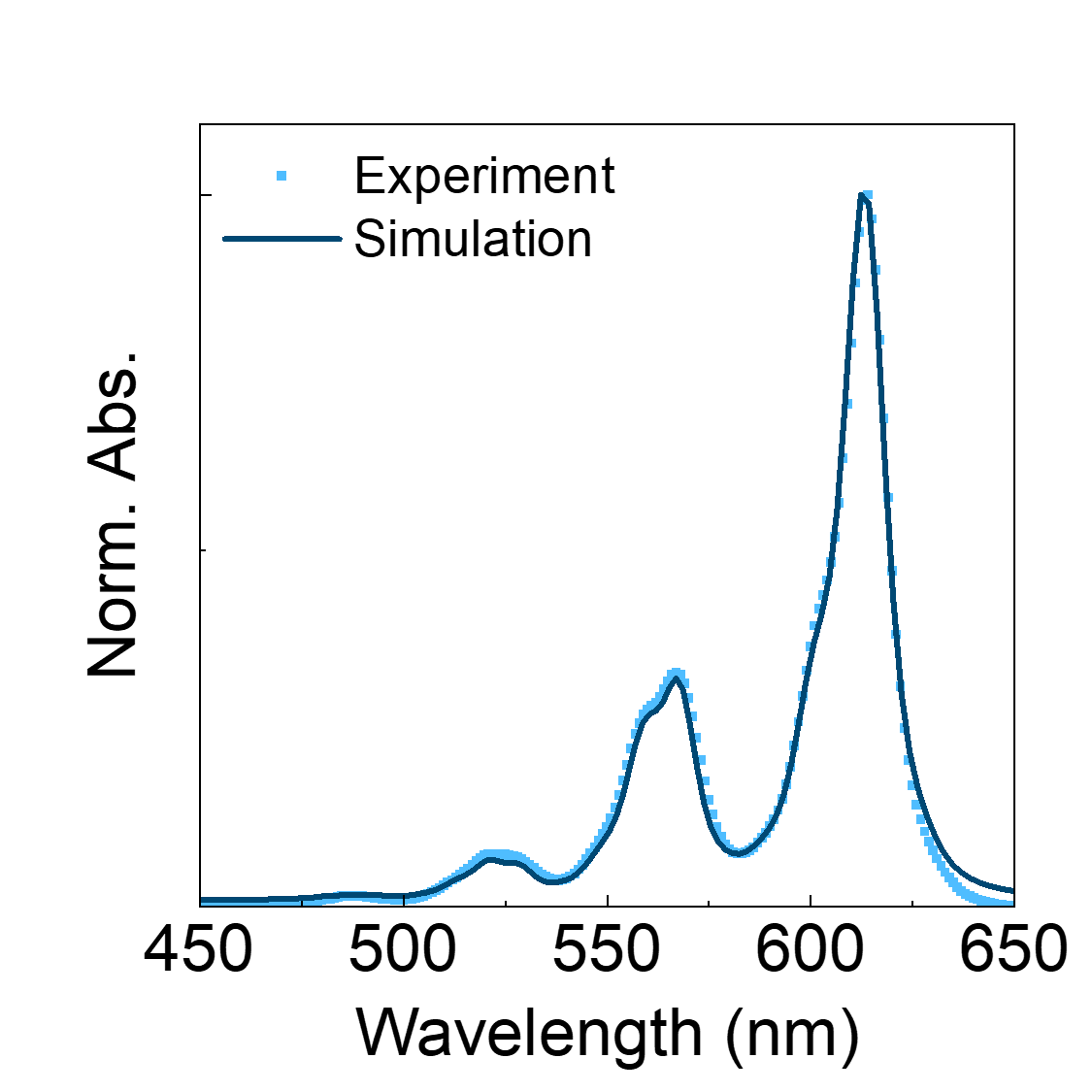


**Figure S6.** Experimental and simulated absorption spectra of Cl-DBOV-Mes in toluene solution at 0.1 mg mL^-1^ (blue dots and full line, respectively). The simulation parameters are in listed in Table S1. The measurements were performed with 1 mm cuvettes.


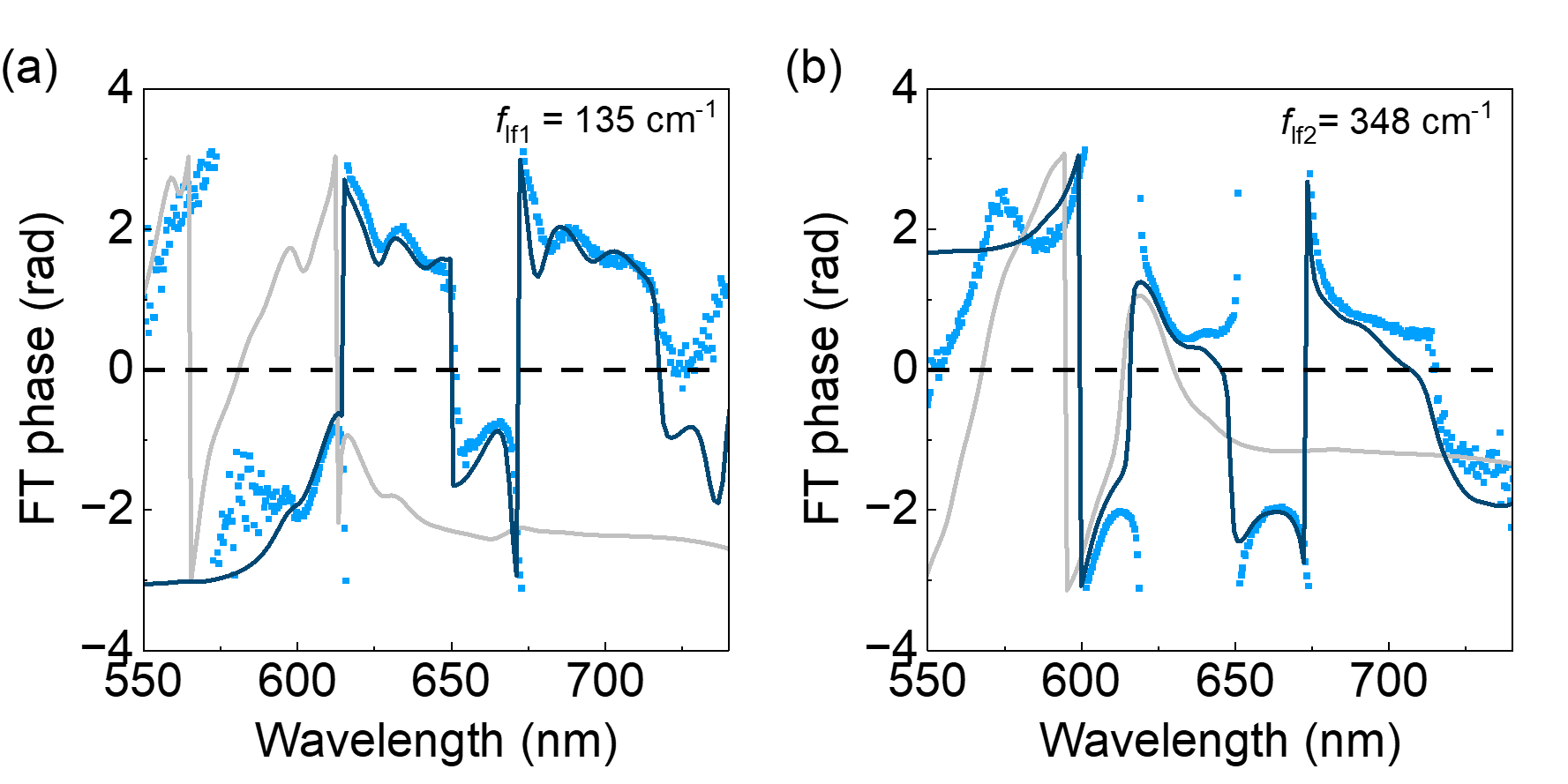


**Figure S7.** Experimental phase femtosecond coherence spectra of Cl-DBOV-Mes in toluene solution (1 mg mL^-1^; 0.2 mm cuvette; blue squares) for the modes at (a) 135 and (b) 348 cm^-1^ and corresponding simulated contributions from the ground (grey line) and excited states (blue line).

**Table S1.** Frequency modes and associated dimensionless displacements used for the full-quantum simulation of the coherent oscillations of Cl-DBOV-Mes. The electronic dephasing was set at *T*_2_ = 68 fs, the temperature at *T* = 300 K and the gap energy at 614 nm.

| Mode | frequency, *f* [cm^-1^] | Displacement, Δ |
| --- | --- | --- |
| lf1 | 135 | 0.6 |
| lf2 | 348 | 0.6 |
| D | 1310 | 0.7 |
| G | 1570 | 0.5 |
| Inh | 7 | 1.5 |


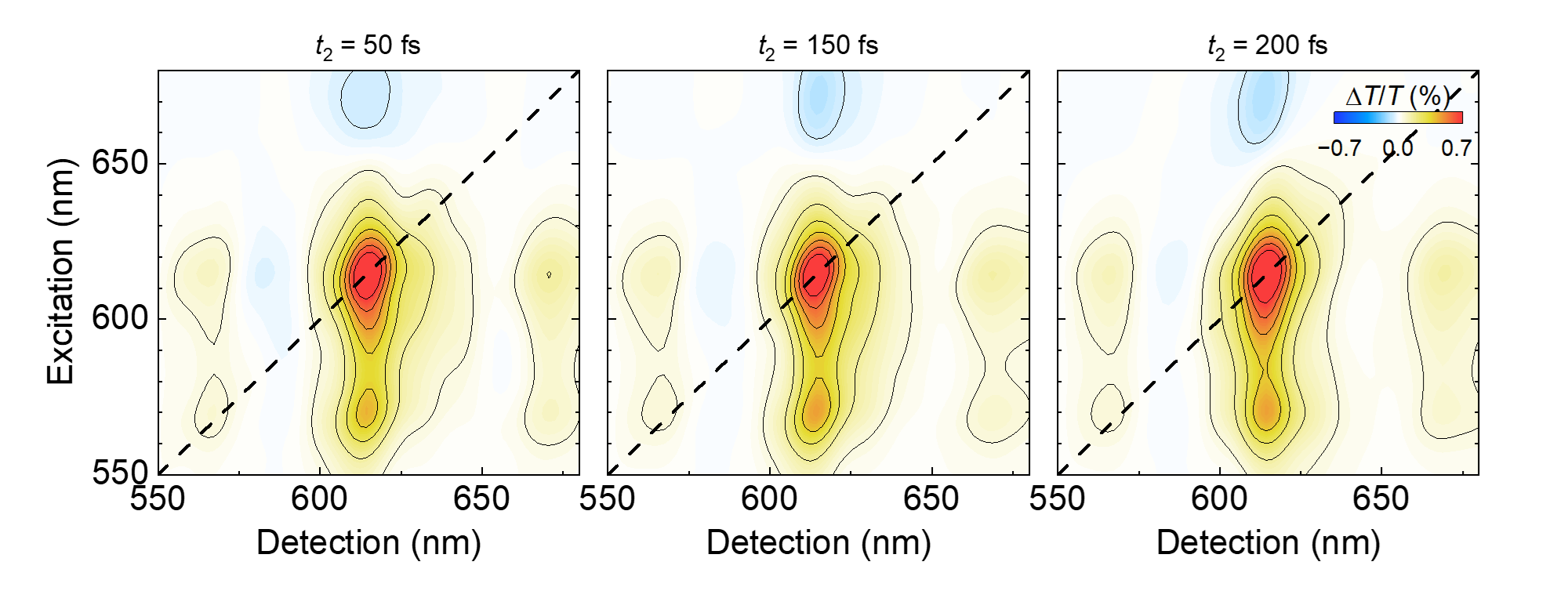


**Figure S8.** Two-dimensional electronic spectroscopy (2DES) maps at *t*_2_ = 50, 150 and 200 fs for Cl-DBOV-Mes in toluene solution (1 mg mL^-1^; 0.2 mm cuvette).


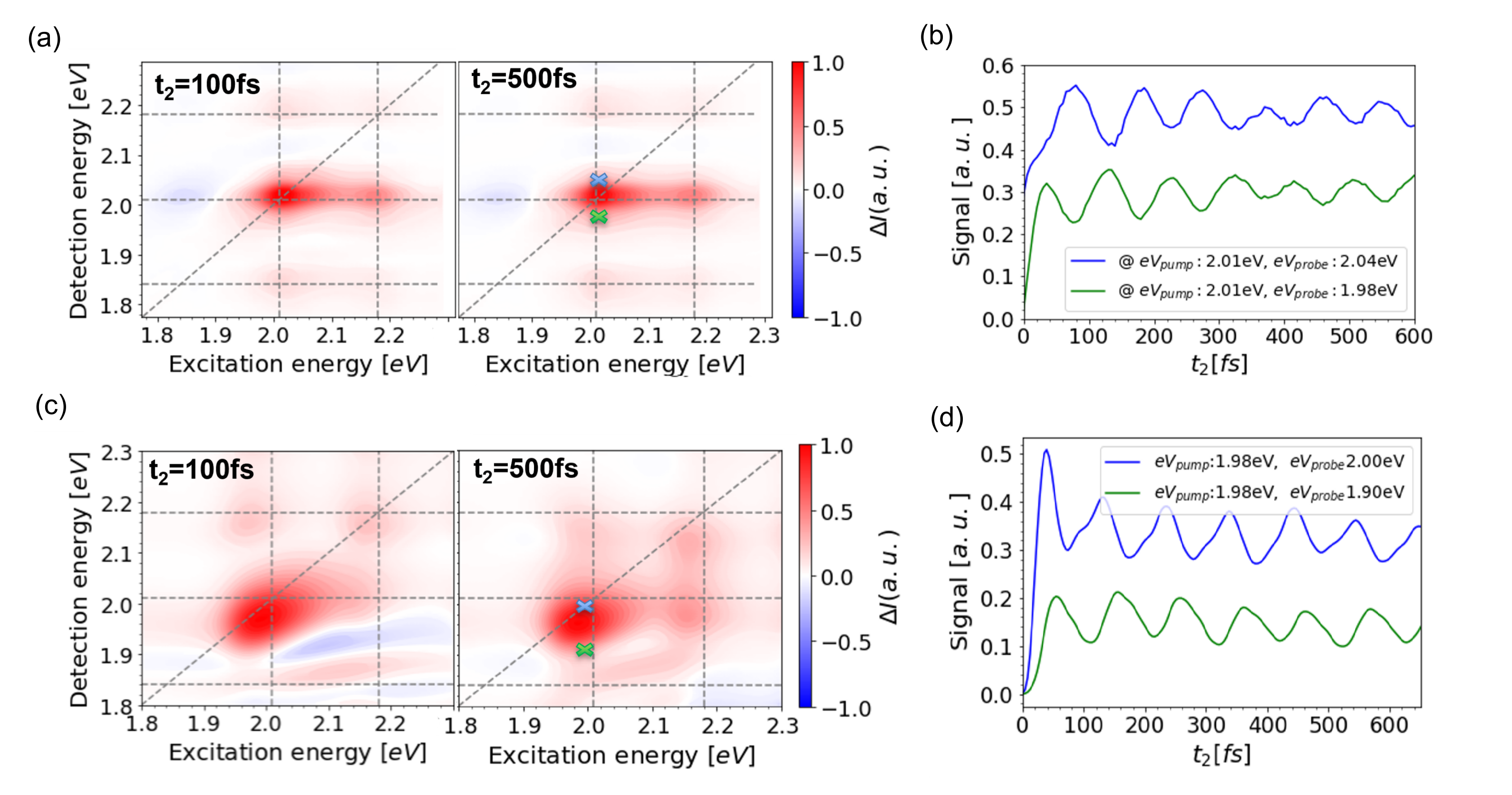


**Figure S9.** (a) Experimental 2DES maps of Cl-DBOV-Mes at *t*_2_ = 100 and 500 fs. (b) 2DES dynamics for the peaks obtained by exciting at 2.01 eV (617 nm) and detecting at 2.04 eV (608 nm) and 1.98 eV (626 nm). (c) Simulated 2DES maps of Cl-DBOV-Mes at *t*_2_ = 100 and 500 fs. (d) Simulated 2DES dynamics of the peaks obtained by exciting at 1.98 eV (626 nm) and detecting at 2 eV (620 nm) and 1.90 eV (653 nm). The details of the simulation are reported elsewhere.^[4]^


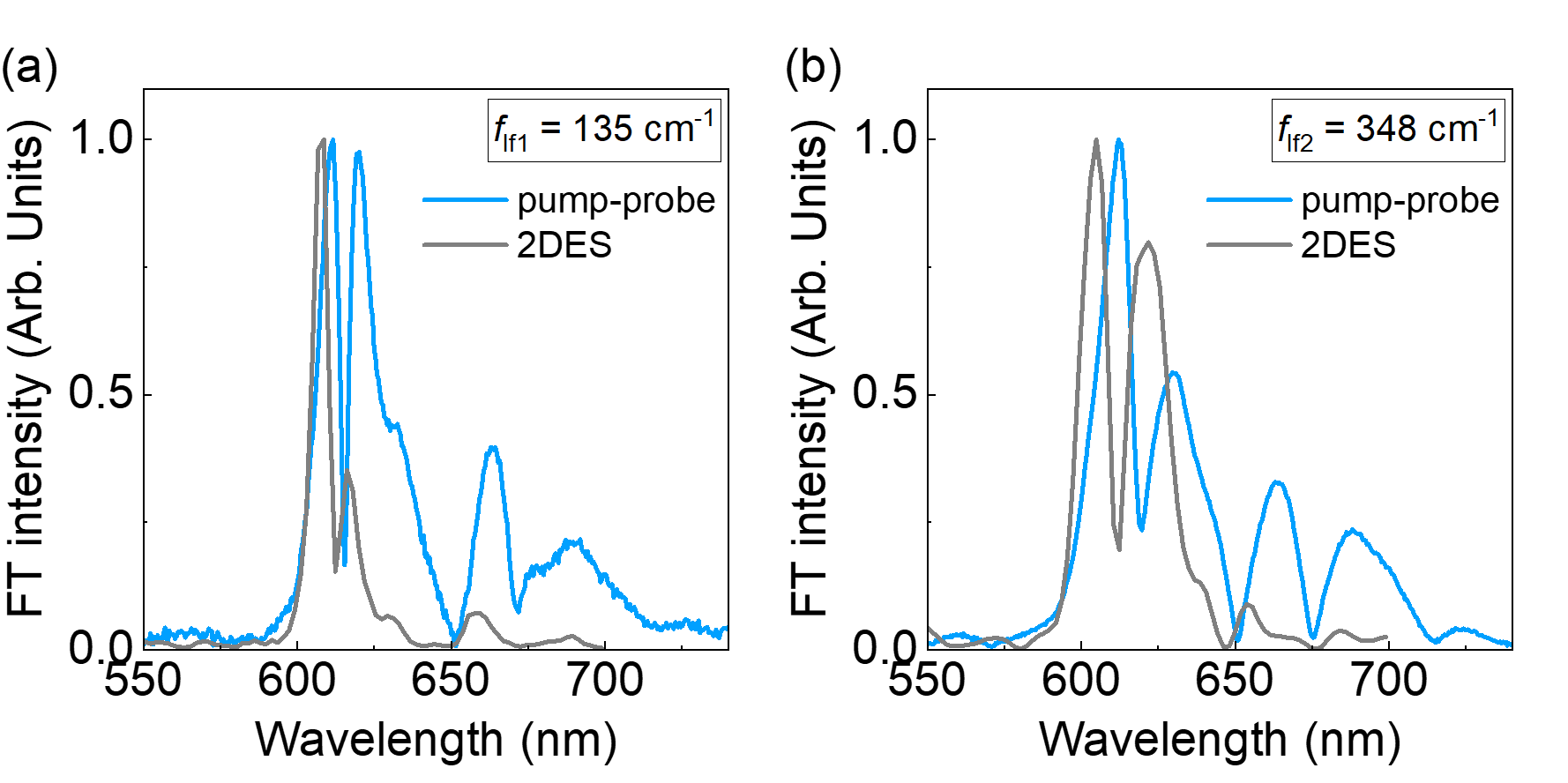


**Figure S10.** Femtosecond coherence spectra (blue line) and integrated 2D beating maps (grey line) of Cl-DBOV-Mes in toluene solution (1 mg mL^-1^; 0.2 mm cuvette).

**Figure S11.** Map of the difference of the bond lengths (pm) between the equilibrium structure of the lowest bright excited state and the ground state of DBOV-Mes and Cl-DBOV-Mes.

**Table S2.** Huang-Rhys factors of DBOV-Mes and Cl-DBOV-Mes obtained from TD-B3LYP/6-31G(d,p) calculations.

| DBOV-Mes | | | Cl-DBOV-Mes | | |
| --- | --- | --- | --- | --- | --- |
| Wavenumber (cm^-1^) | HR factor | Raman activity@405 nm (Å^4^/amu) | Wavenumber (cm^-1^) | HR factor | Off-resonance Raman activity (Å^4^/amu) |
| 138 | 0.083 | 35 | 134 | 0.106 | 2 |
| 281 | 0.000 | 648 | 233 | 0.011 | 125 |
| 332 | 0.088 | 102 | 355 | 0.143 | 178 |
| 357 | 0.114 | 970 | 1273 | 0.063 | 5893 |
| 410 | 0.002 | 509 | 1380 | 0.102 | 9255 |
| 411 | 0.002 | 648 | 1604 | 0.063 | 12972 |
| 1284 | 0.096 | 37823 |  |  |  |
| 1383 | 0.099 | 40298 |  |  |  |
| 1396 | 0.006 | 68658 |  |  |  |
| 1607 | 0.038 | 6415 |  |  |  |
| 1617 | 0.041 | 37244 |  |  |  |
| 1646 | 0.007 | 77085 |  |  |  |

**Mulliken and natural bond orbital (NBO) charge analyses:**

Figure S12 (a, b) shows the Mulliken charge distribution of **DBOV-Mes** and **Cl-DBOV-Mes**. In the left and center panels, the hydrogen atoms of both molecules are predicted to be positively charged, while their neighboring carbon atoms show a partial negative charge; moreover, the center of the graphenic core of both molecules is almost neutral. In **Cl-DBOV-Mes**, the two chlorine atoms are negatively charged, as expected from their relatively high electronegativity. As shown in Figure S12 (c), where we report the difference between the Mulliken charge of **Cl-DBOV-Mes** and **DBOV-Mes**, the introduction of the two chlorine atoms causes a perturbation of the charge distribution that is localized near the chlorine atoms and the rings that carry them. The charge difference map shows that, upon functionalization, the carbon atoms bonded to the chlorine atoms acquire a negative charge, whereas the second-nearest neighboring carbon atoms and the bonded hydrogen atoms acquire a positive charge. The natural bond orbital (NBO) charge distribution analysis, reported in Figure S13, emphasizes this picture. In particular, NBO still predicts charge localization at the periphery of **DBOV-Mes** and **Cl-DBOV-Mes**, with neutral graphenic cores, and the perturbation of the charge distribution caused by chlorination which is localized nearby the chlorine atoms. The main difference with respect to the Mulliken charge distribution analysis is that the carbon atom bonded to chlorine acquires a significant positive charge upon chlorination.

|  |  |  |
| --- | --- | --- |
| (a) **DBOV-Mes** | (b) **Cl-DBOV-Mes** | (c) **Cl-DBOV-Mes** – **DBOV-Mes** |

**Figure S12.** Mulliken charge analysis of **DBOV-Mes** and **Cl-DBOV-Mes**. Panels (a, b) show the Mulliken charges of **DBOV-Mes** and **Cl-DBOV-Mes** represented as circles with their area proportional to the magnitude of the charge and their color (red or blue) representing the sign of the charge (positive or negative, respectively). Panel (c) shows the difference between the Mulliken charges of **DBOV-Mes** and **Cl-DBOV-Mes** projected on the optimized geometry of **Cl-DBOV-Mes**. In panel (c), the charge q reported at the chlorine position is q(Cl) – q(H). The units on all the Cartesian axes are Å.

|  |  |  |
| --- | --- | --- |
| (a) **DBOV-Mes** | (b) **Cl-DBOV-Mes** | (c) **Cl-DBOV-Mes** – **DBOV-Mes** |

**Figure S13.** NBO charge analysis of **DBOV-Mes** and **Cl-DBOV-Mes**. Panels (a, b) show the NBO charges of **DBOV-Mes** and **Cl-DBOV-Mes** represented as circles with their area proportional to the magnitude of the charge and their color (red or blue) representing the sign of the charge (positive or negative, respectively). Panel (c) shows the difference between the NBO charges of **DBOV-Mes** and **Cl-DBOV-Mes** projected on the optimized geometry of **Cl-DBOV-Mes**. In panel (c), the charge q reported at the chlorine position is q(Cl) – q(H). The units on all the Cartesian axes are Å.


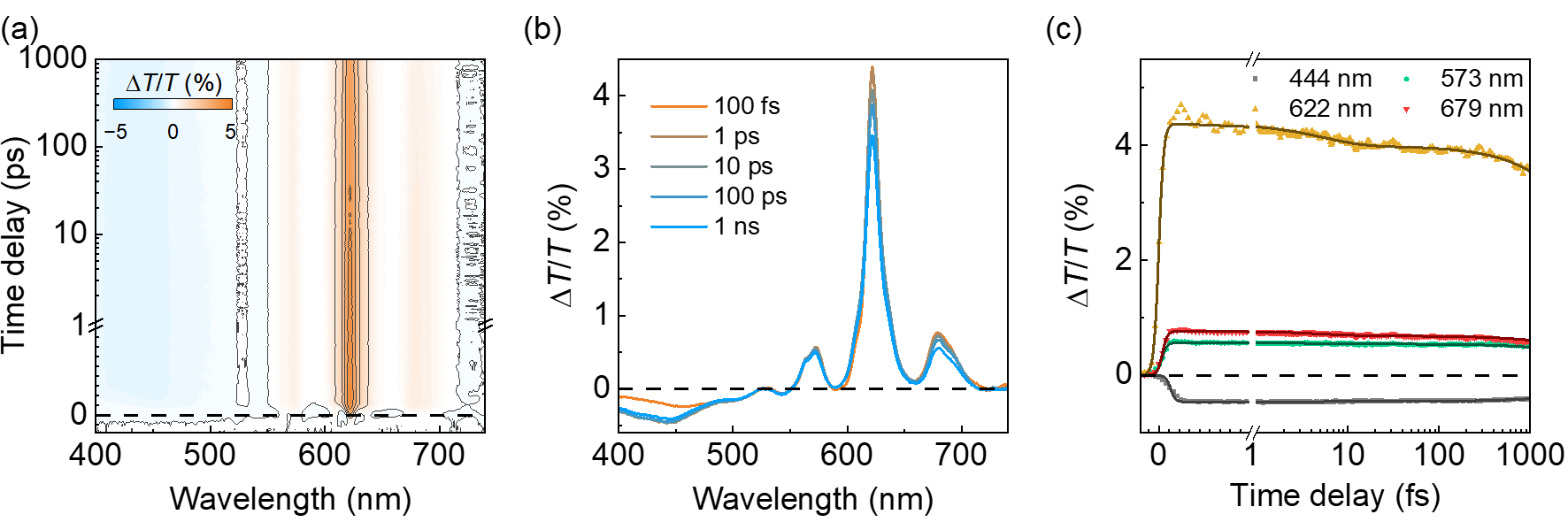


**Figure S14.** (a) Differential transmission (Δ*T*/*T*) map of DBOV-Mes in toluene solution (0.1 mg mL^-1^; 1 mm cuvette) as a function of wavelength and time delay. (b) Δ*T*/*T* spectra at various time delays. (c) Time traces at 444 (grey squares; ESA), 573 (green circles; GSB), 622 (orange up triangles; GSB+SE) and 679 nm (red down triangles; SE) probe wavelengths. Full lines are exponential fits to the data. The sample was pump at *λ*_pump_ = 610 nm with a fluence *F*_pump_ = 41 µJ cm^-2^ (repetition rate = 1 kHz; Δ*t*_pump_ = 70 fs).


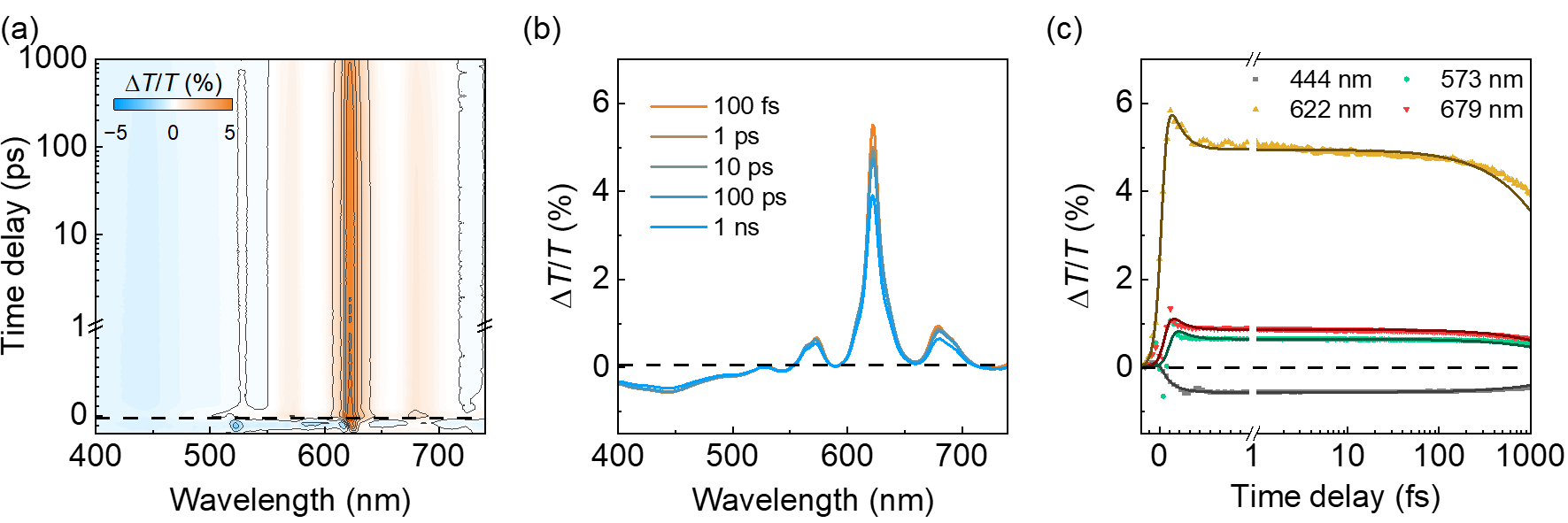


**Figure S15.** (a) Differential transmission (Δ*T*/*T*) map of DBOV-Mes in toluene solution (0.01 mg mL^-1^; 1 mm cuvette) as a function of wavelength and time delay. (b) Δ*T*/*T* spectra at various time delays. (c) Time traces at 444 (grey squares; ESA), 573 (green circles; GSB), 622 (orange up triangles; GSB+SE) and 679 nm (red down triangles; SE) probe wavelengths. Full lines are exponential fits to the data. The sample was pump at *λ*_pump_ = 610 nm with a fluence *F*_pump_ = 410 µJ cm^-2^ (repetition rate = 1 kHz; Δ*t*_pump_ = 70 fs).


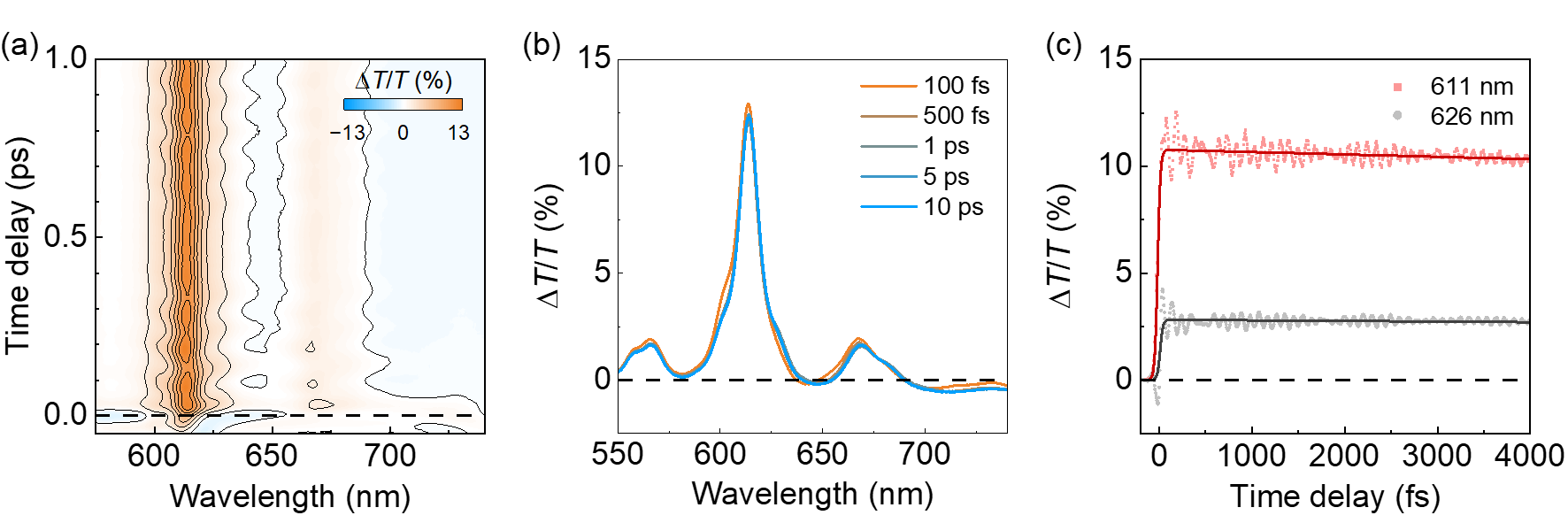


**Figure S16.** (a) Differential transmission Δ*T*/*T*) map of DBOV-Mes in toluene solution (1 mg mL^-1^; 0.2 mm cuvette) as a function of wavelength and time delay. (b) Δ*T*/*T* spectra at various time delays. (c) Time traces at 611 (red squares) and 626 nm (grey circles) probe wavelengths. Full lines are exponential fits to the data. The sample was pump with 600 µJ cm^-2^ (repetition rate = 1 kHz) broad band excitation pulses centred at 600 nm that provided high temporal resolution (15 fs).


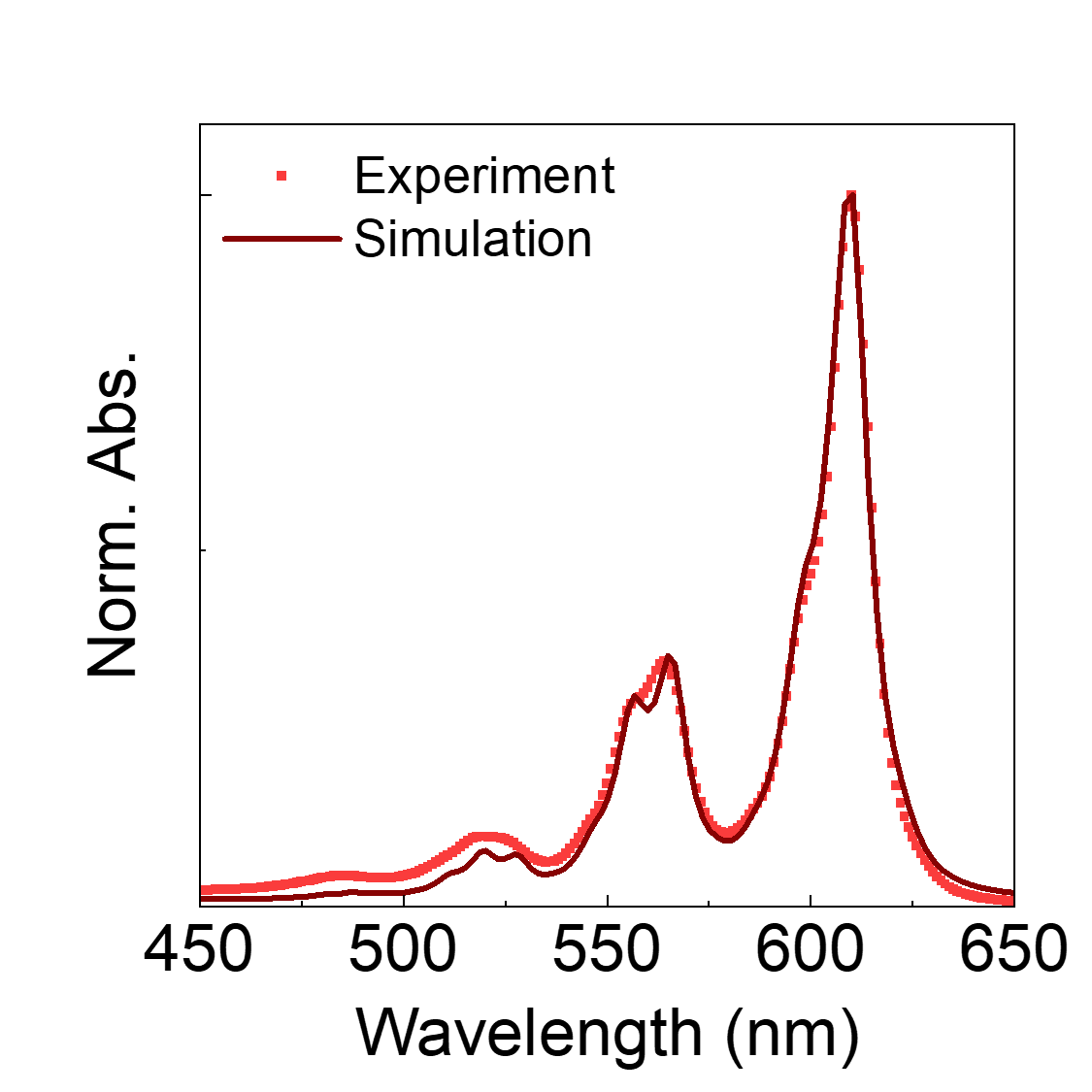


**Figure S17.** Experimental and simulated absorption spectra of DBOV-Mes in toluene solution at 0.1 mg mL^-1^ (red dots and full line, respectively). The simulation parameters are in listed in Table S3. The measurements were performed with 1 mm cuvettes.


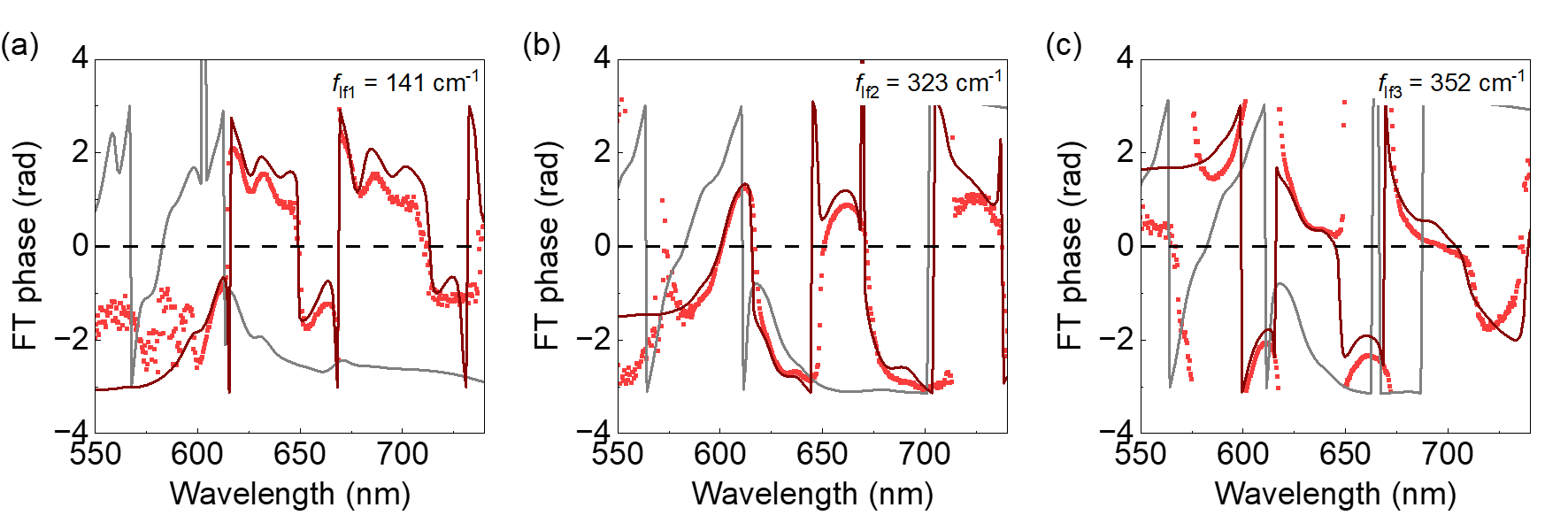


**Figure S18.** Experimental phase femtosecond coherence spectra of DBOV-Mes in toluene solution (1 mg mL^-1^; 0.2 mm cuvette; red squares) for the modes at (a) 141 (b) 323 and (c) 352 cm^-1^ and corresponding simulated contributions from the ground (grey line) and excited states (red line).

**Table S3.** Frequency modes and associated dimensionless displacements used for the full-quantum simulation of the coherent oscillations of DBOV-Mes. The electronic dephasing was set at *T*_2_ = 82 fs, the temperature at *T* = 300 K and the gap energy at 610 nm.

| Mode | frequency, *f* [cm^-1^] | Displacement, Δ |
| --- | --- | --- |
| lf1 | 141 | 0.6 |
| lf2 | 323 | 0.5 |
| lf3 | 352 | 0.4 |
| D | 1270 | 0.75 |
| G | 1570 | 0.4 |
| Inh | 12 | 1.2 |


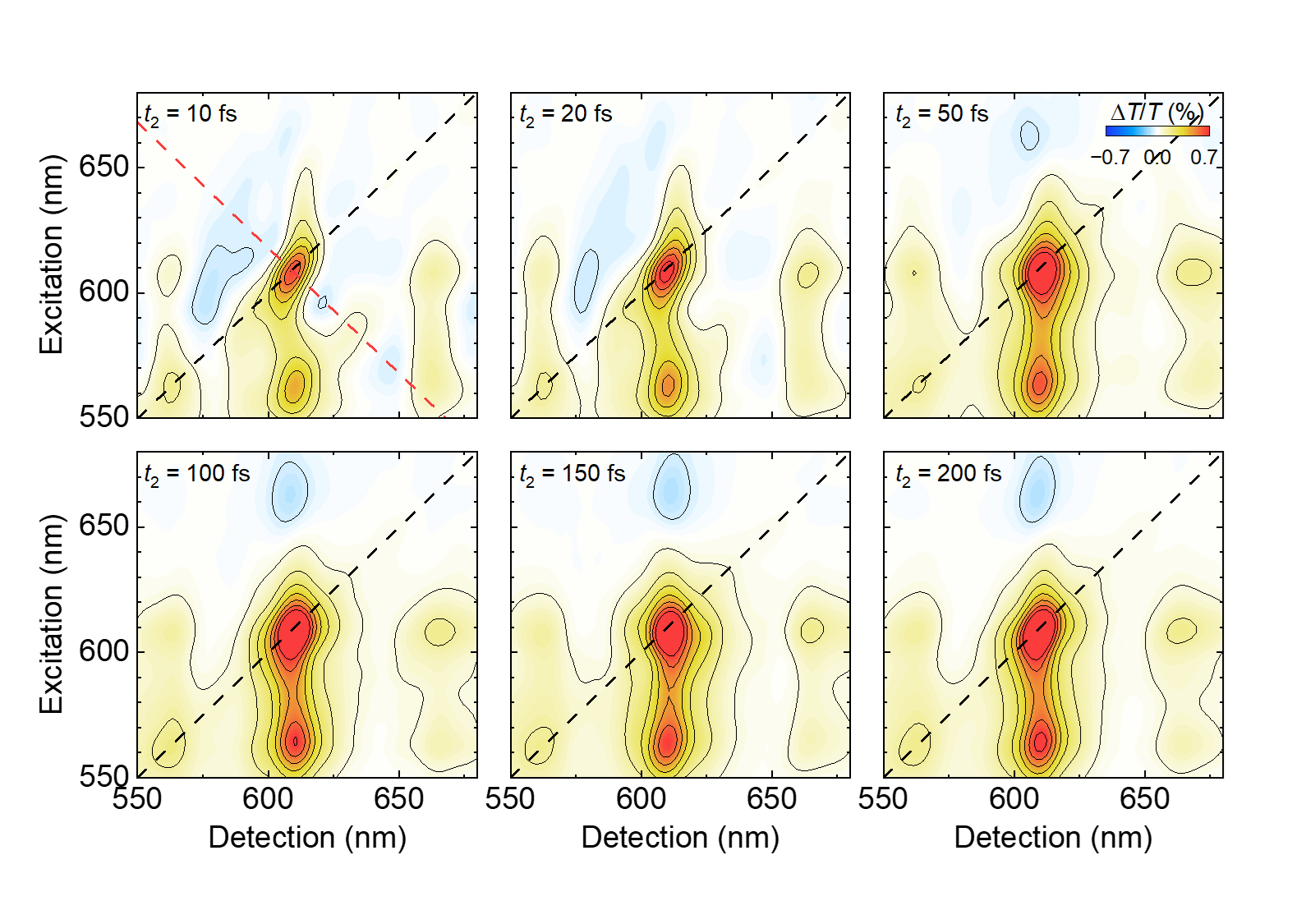


**Figure S19.** Two-dimensional electronic spectroscopy (2DES) maps at *t*_2_ = 10, 20, 50, 100, 150 and 200 fs for DBOV-Mes in toluene solution (1 mg mL^-1^; 0.2 mm cuvette).


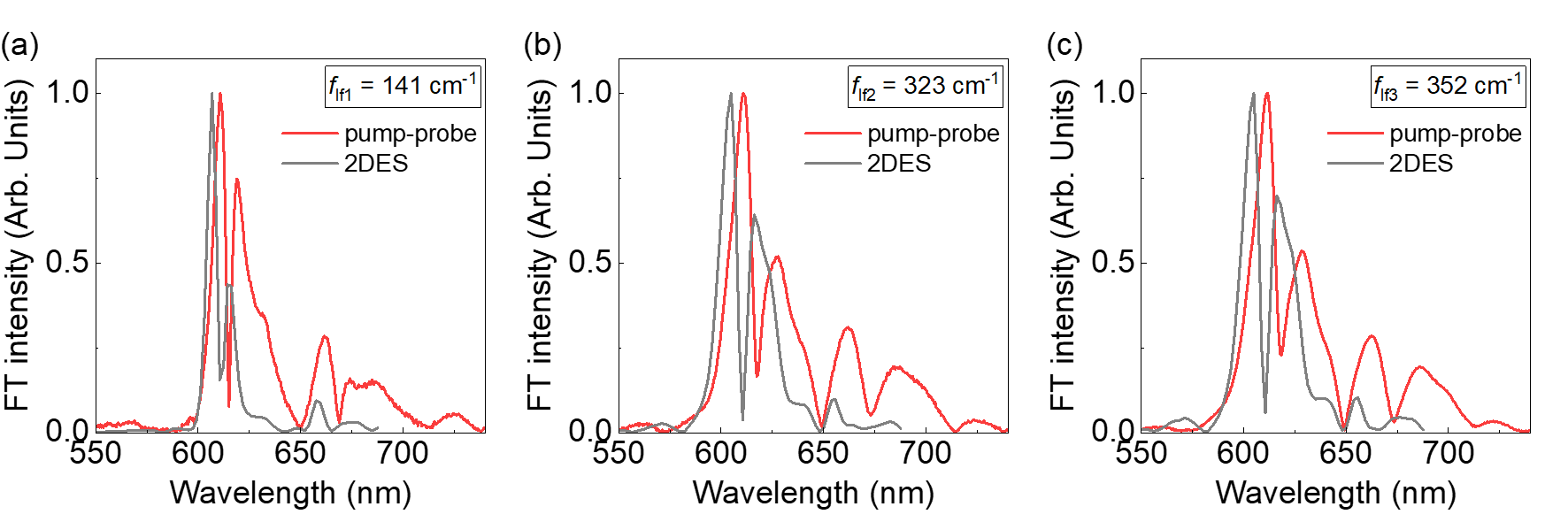


**Figure S20.** Femtosecond coherence spectra (red line) and integrated 2D beating maps (grey line) of DBOV-Mes in toluene solution (1 mg mL^-1^; 0.2 mm cuvette).


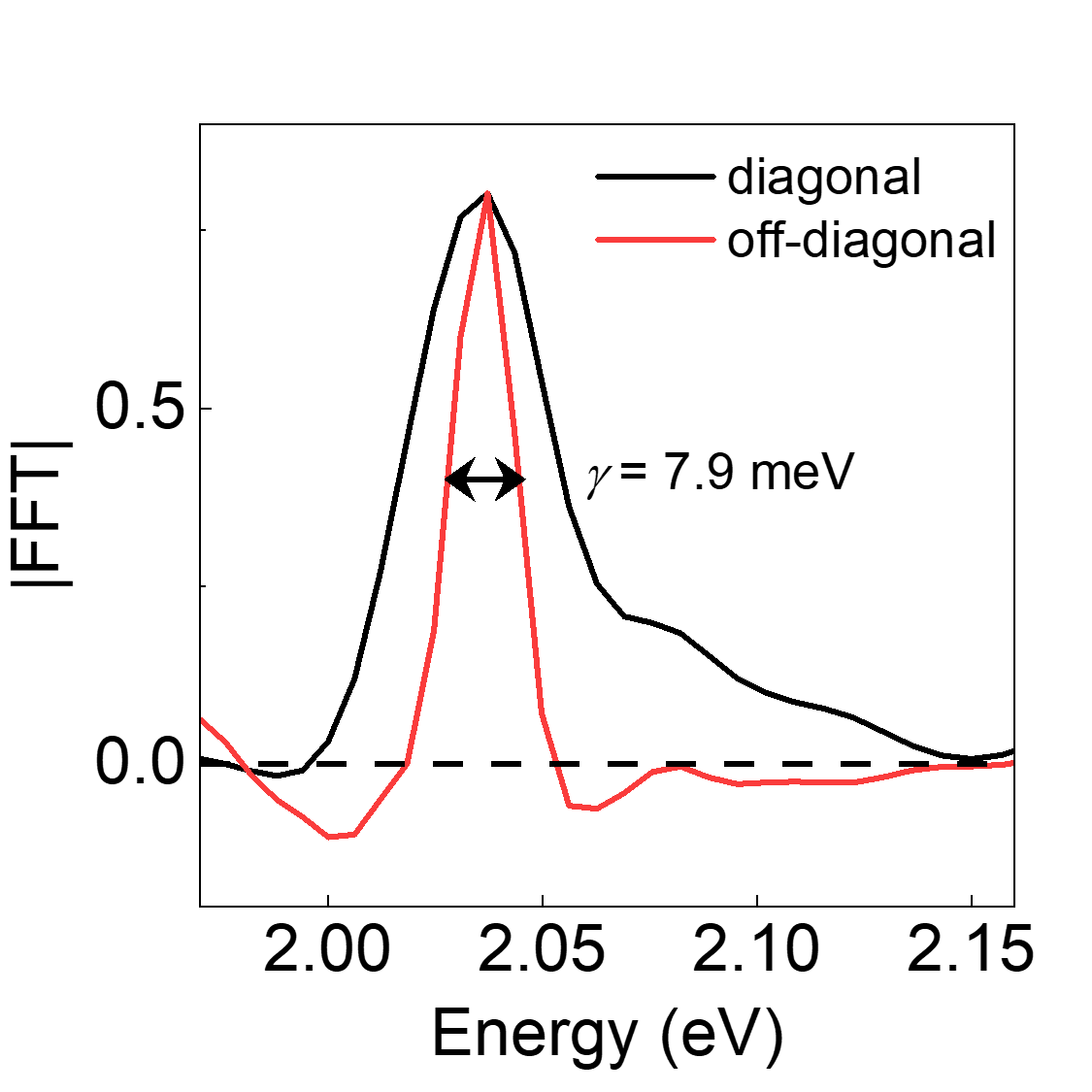


**Figure S21.** Diagonal (black) and off-diagonal (red) cuts of the 2D map acquired at *t*_2_ = 0 fs for DBOV-Mes in toluene solution (1 mg mL^-1^; 0.2 mm cuvette).

## NMR Spectra

**Figure S22.** ^1^H NMR spectrum of compound **2** (CDCl_3_, 400 MHz, 298 K).

**Figure S23.** ^13^C NMR spectrum of compound **2** (CDCl_3_, 126 MHz, 298 K).

**Figure S24.** ^1^H NMR spectrum of compound **3** (CDCl_3_, 400 MHz, 298 K).

**Figure S25.** ^13^C NMR spectrum of compound **3** (CDCl_3_, 126 MHz, 298 K).

**Figure S26.** ^1^H NMR spectrum of compound **5** (CDCl_3_, 500 MHz, 298 K).

**Figure S27.** ^13^C NMR spectrum of compound **5** (CDCl_3_, 126 MHz, 298 K).

**Figure S28.** ^1^H NMR spectrum of compound **6** (CDCl_3_, 500 MHz, 298 K).

**Figure S29.** ^13^C NMR spectrum of compound **6** (CDCl_3_, 126 MHz, 298 K).

**Figure S30.** ^1^H NMR spectrum of compound **7** (CDCl_3_, 500 MHz, 298 K).

**Figure S31.** ^13^C NMR spectrum of compound **7** (CDCl_3_, 126 MHz, 298 K).

**Figure S32.** ^1^H NMR spectrum of compound **8** (CDCl_3_, 400 MHz, 298 K).

**Figure S33.** ^13^C NMR spectrum of compound **8** (CDCl_3_, 126 MHz, 298 K).

**Figure S34.** ^1^H NMR spectrum of compound **9** (CDCl_3_, 400 MHz, 298 K).

**Figure S35.** ^13^C NMR spectrum of compound **9** (CDCl_3_, 126 MHz, 298 K).

**Figure S36.** ^1^H NMR spectrum of **Cl-DBOV-Mes** (THF-*d_8_*:CS_2_=1:1, 500 MHz, 298 K).

**Figure S37.** ^13^C NMR spectrum of **Cl-DBOV-Mes** (THF-*d_8_*:CS_2_=1:1, 126 MHz, 298 K).

## Mass Spectra

**Figure S38.** High-resolution APCI MS spectrum of compound **2**.

**Figure S39.** Experimental (red) and simulated (black) isotopic distributions of compound **2** (HRMS APCI).

**Figure S40.** High-resolution APCI MS spectrum of compound **3**.

**Figure S41.** Experimental (red) and simulated (black) isotopic distributions of compound **3** (HRMS APCI).

**Figure S42.** High-resolution APCI MS spectrum of compound **5**.

**Figure S43.** Experimental (red) and simulated (black) isotopic distributions of compound **5** (HRMS APCI).

**Figure S44.** High-resolution APCI MS spectrum of compound **6**.

**Figure S45.** Experimental (red) and simulated (black) isotopic distributions of compound **6** (HRMS APCI).

**Figure S46.** High-resolution APCI MS spectrum of compound **7**.

**Figure S47.** Experimental (red) and simulated (black) isotopic distributions of compound **7** (HRMS APCI).

**Figure S48.** High-resolution APCI MS spectrum of compound **8**.

**Figure S49.** Experimental (red) and simulated (black) isotopic distributions of compound **8** (HRMS APCI).

**Figure S50.** High-resolution MALDI-TOF MS spectrum of compound **9**.

**Figure S51.** Experimental (red) and simulated (black) isotopic distributions of compound **9** (HRMS MALDI-TOF).

**Figure S52.** High-resolution MALDI-TOF MS spectrum of **Cl-DBOV-Mes**.

**Figure S53.** Experimental (red) and simulated (black) isotopic distributions of **Cl-DBOV-Mes** (HRMS-MALDI-TOF).

References

[1] G. M. Paternò, Q. Chen, X. Y. Wang, J. Liu, S. G. Motti, A. Petrozza, X. Feng, G. Lanzani, K. Müllen, A. Narita, F. Scotognella, *Angew. Chem., Int. Ed.* **2017**, *56*, 6753.

[2] M. J. Frisch, G. W. Trucks, H. B. Schlegel, G. E. Scuseria, M. A. Robb, J. R. Cheeseman, G. Scalmani, V. Barone, G. A. Petersson, H. Nakatsuji, X. Li, M. Caricato, A. Marenich, J. Bloino, B. G. Janesko, R. Gomperts, B. Mennucci, H. P. Hratchian, J. V. Ortiz, A. F. Izmaylov, J. L. Sonnenberg, D. Williams-Young, F. Ding, F. Lipparini, F. Edigi, J. Goings, B. Peng, A. Petrone, T. Henderson, D. Ranasinghe, V. G. Zakrzewski, J. Gao, N. Rega, G. Zheng, W. Liang, M. Hada, M. Ehara, K. Toyota, R. Fukuda, J. Hasegawa, M. Ishida, T. Nakajima, Y. Honda, O. Kitao, H. Nakai, T. Vreven, K. Throssell, J. A. Montgomery Jr., J. E. Peralta, F. Ogliaro, M. Bearpark, J. J. Heyd, E. Brothers, K. N. Kudin, V. N. Staroverov, T. Keith, R. Kobayashi, J. Normand, K. Raghavachari, A. Rendell, J. C. Burant, S. S. Iyengar, J. Tomasi, M. Cossi, J. M. Millam, M. Klene, C. Adamo, R. Cammi, J. W. Ochterski, R. L. Martin, K. Morokuma, O. Farkas, J. B. Foresman, D. J. Fox, *Gaussian 09 (Revision A.02)*, Gaussian Inc., Wallingford CT, **2016**.

[3] Y. Aida, J. Nogami, H. Sugiyama, H. Uekusa, K. Tanaka, *Chem. – Eur. J.* **2020**, *26*, 12579.

[4] G. Bassi, Two dimensional electron spectroscopy of graphene nanoribbons: modelling and experiment. Master of Science, Politecnico di Milano, Milano, **2023**.
