# Supplementary material for: Time‐Domain Visualization of Electron‐Phonon Coupling in Nanographenes
Source: Small Methods. 2025 May 19;10(2):2500419. doi: 10.1002/smtd.202500419 (PMC12825327; doi:10.1002/smtd.202500419)
Supplement: Supplementary file 2 — Supporting Information [file SMTD-10-2500419-s001.pptx]

## Slide 1
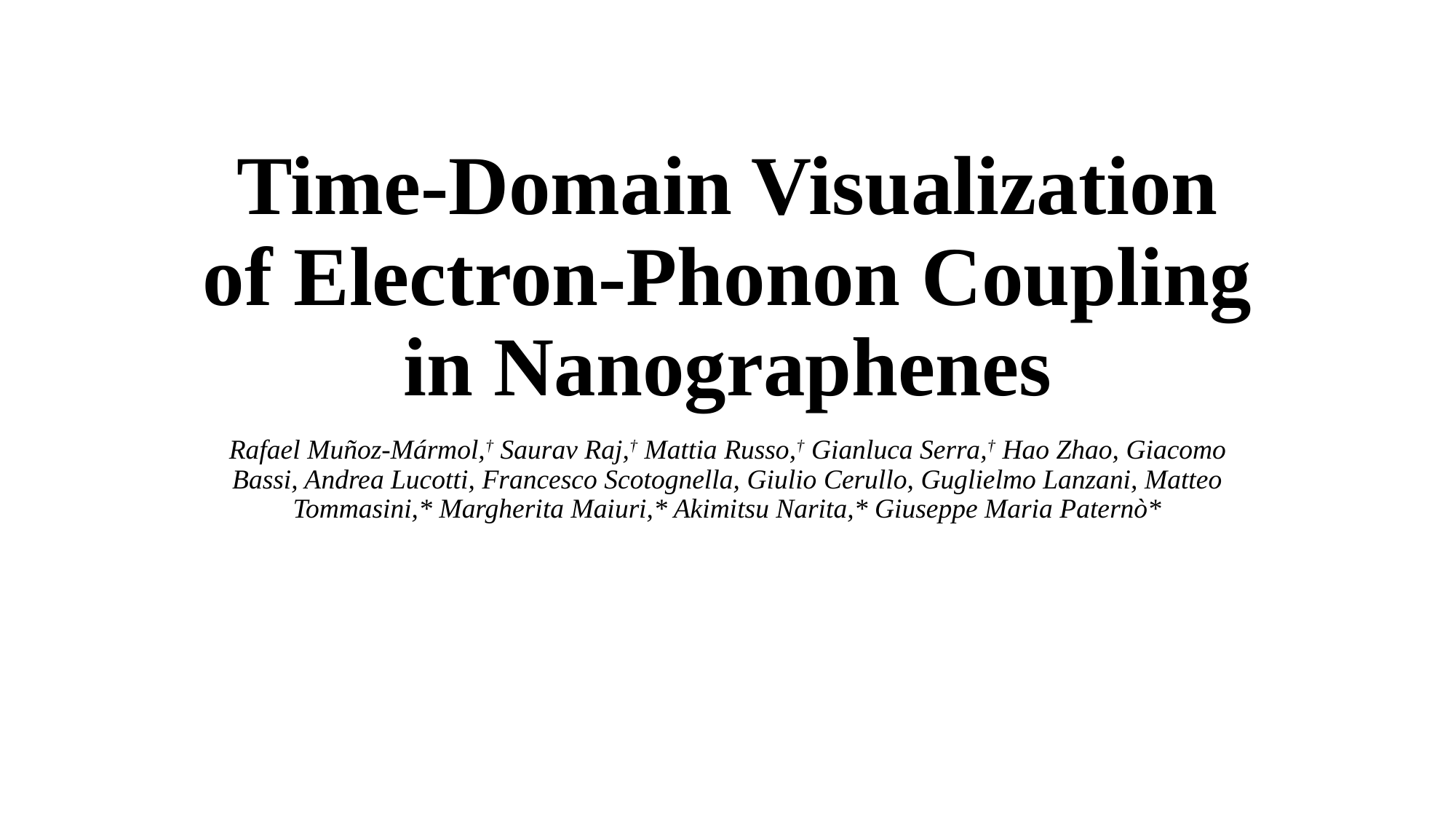

# Time-Domain Visualization of Electron-Phonon Coupling in Nanographenes
Rafael Muñoz-Mármol,† Saurav Raj,† Mattia Russo,† Gianluca Serra,† Hao Zhao, Giacomo Bassi, Andrea Lucotti, Francesco Scotognella, Giulio Cerullo, Guglielmo Lanzani, Matteo Tommasini,* Margherita Maiuri,* Akimitsu Narita,* Giuseppe Maria Paternò*

## Slide 2
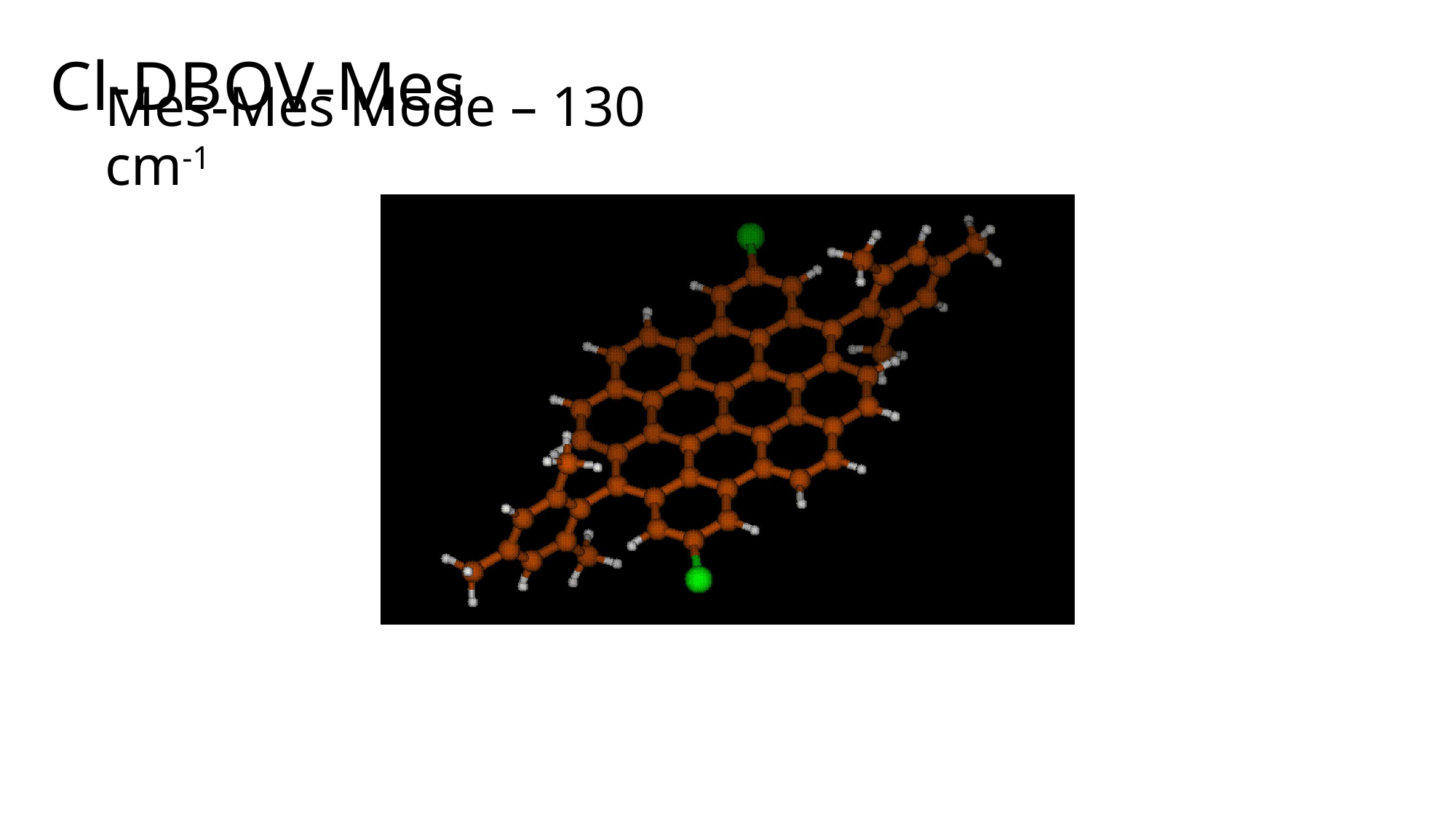

# Cl-DBOV-Mes
Mes-Mes Mode – 130 cm-1

## Slide 3
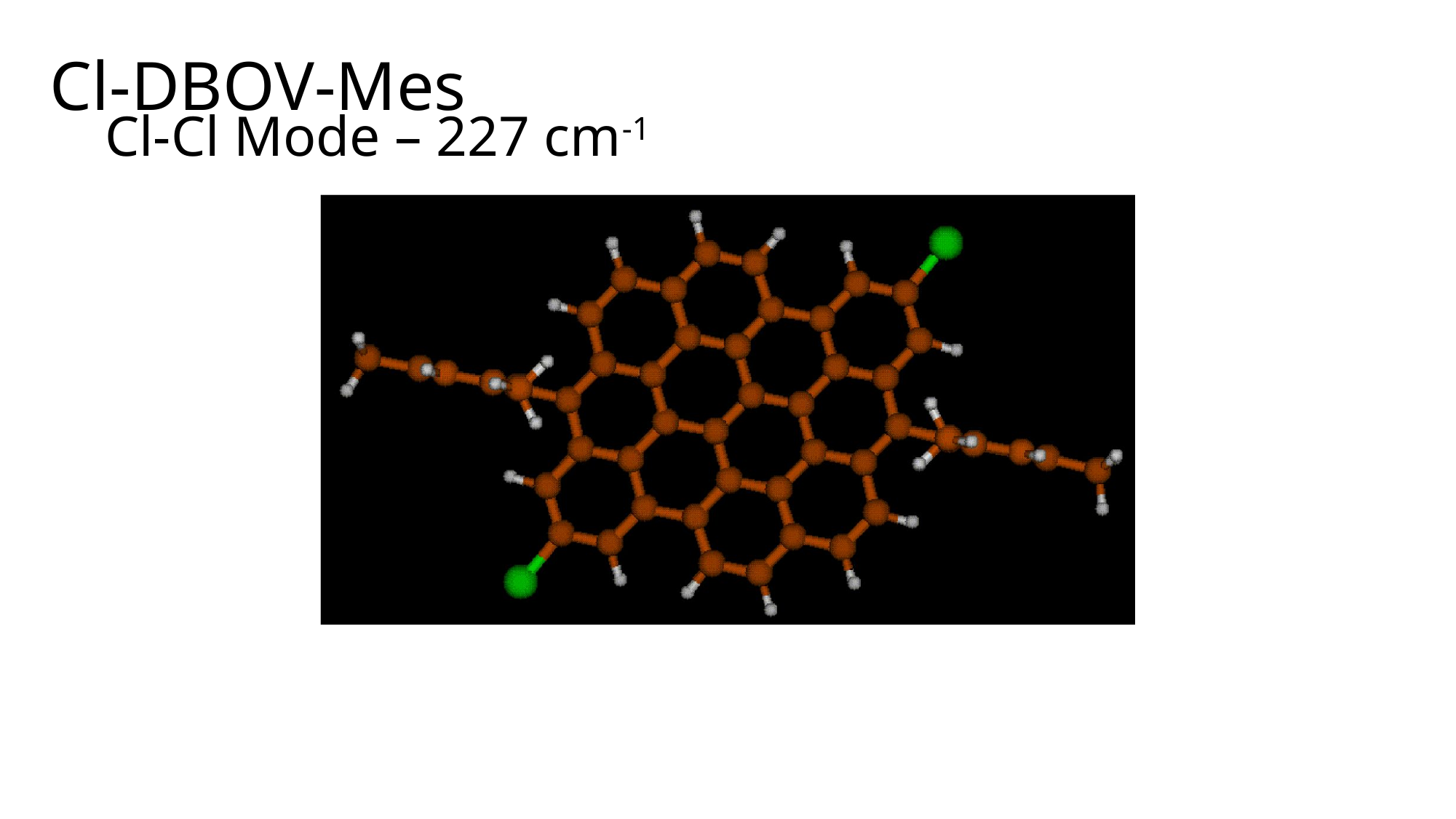

# Cl-DBOV-Mes
Cl-Cl Mode – 227 cm-1

## Slide 4
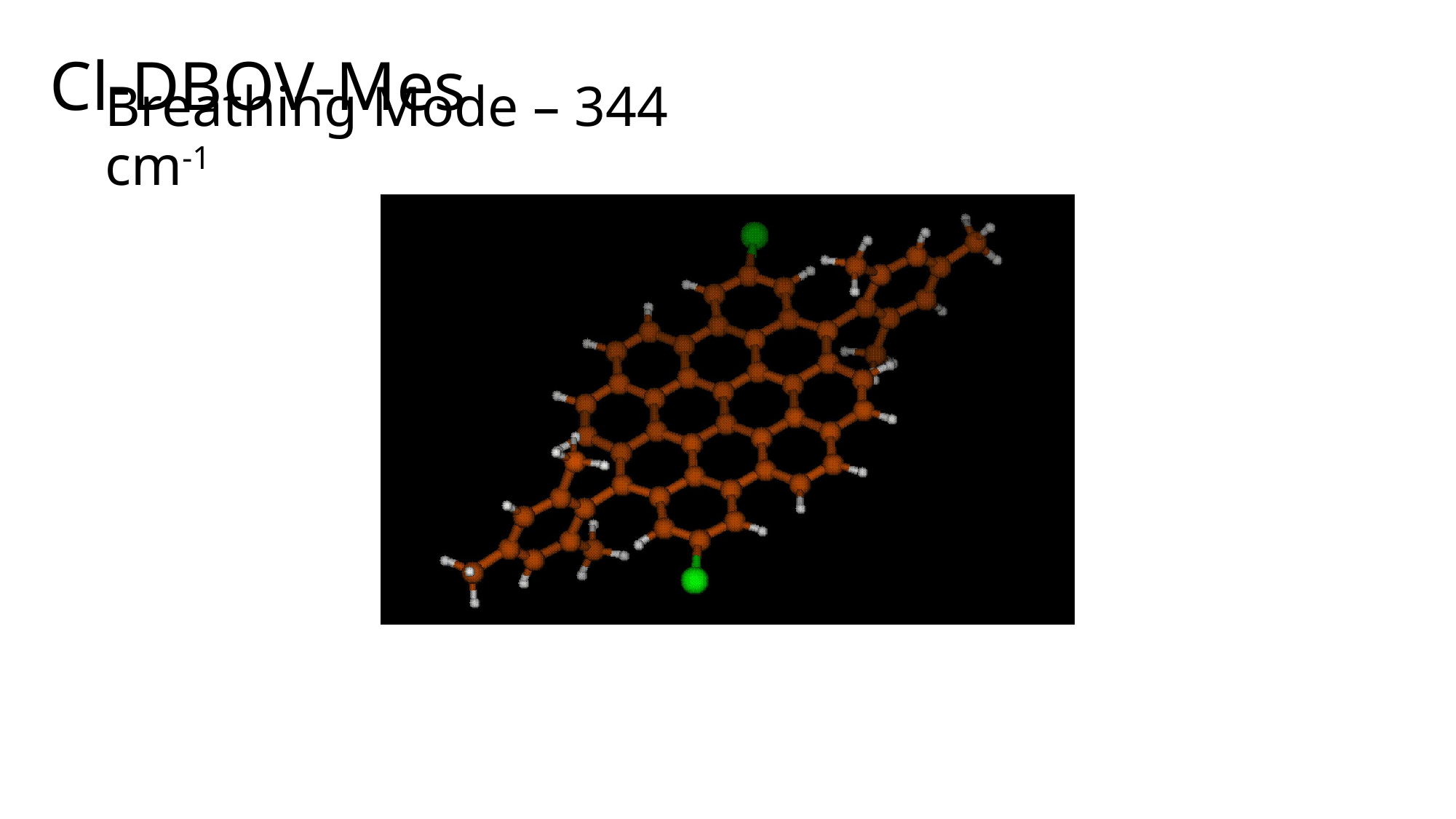

# Cl-DBOV-Mes
Breathing Mode – 344 cm-1

## Slide 5
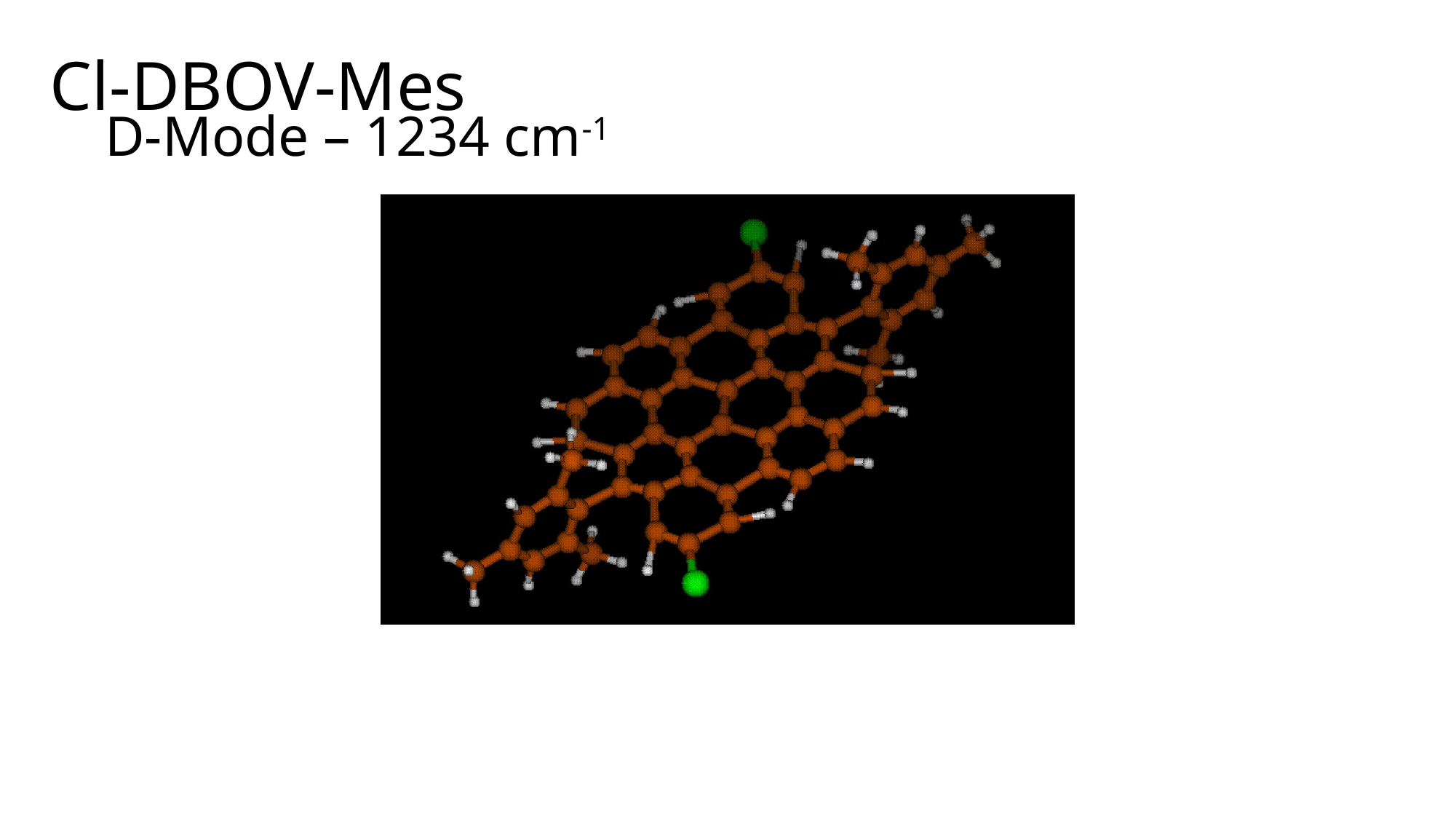

# Cl-DBOV-Mes
D-Mode – 1234 cm-1

## Slide 6
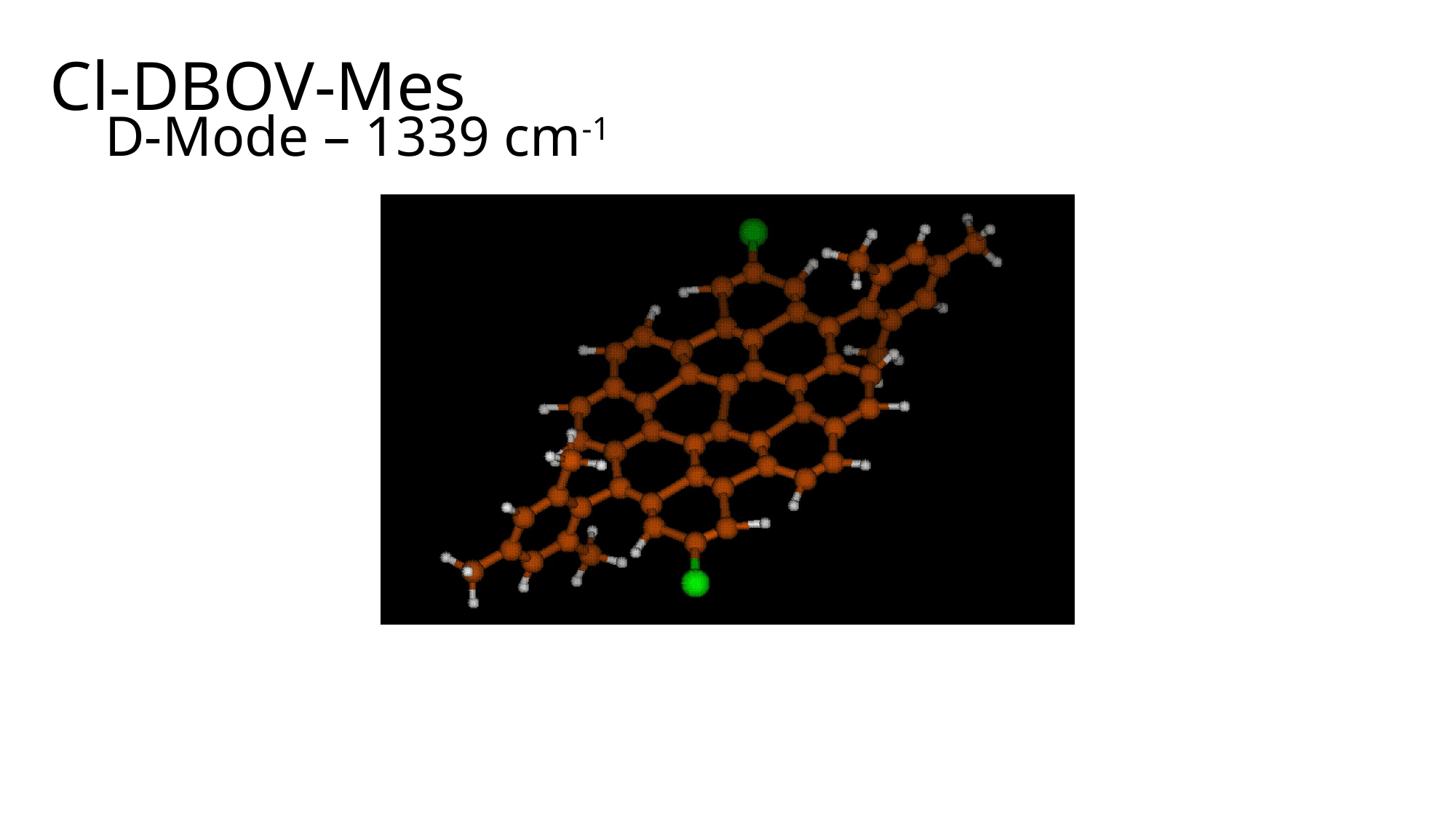

# Cl-DBOV-Mes
D-Mode – 1339 cm-1

## Slide 7
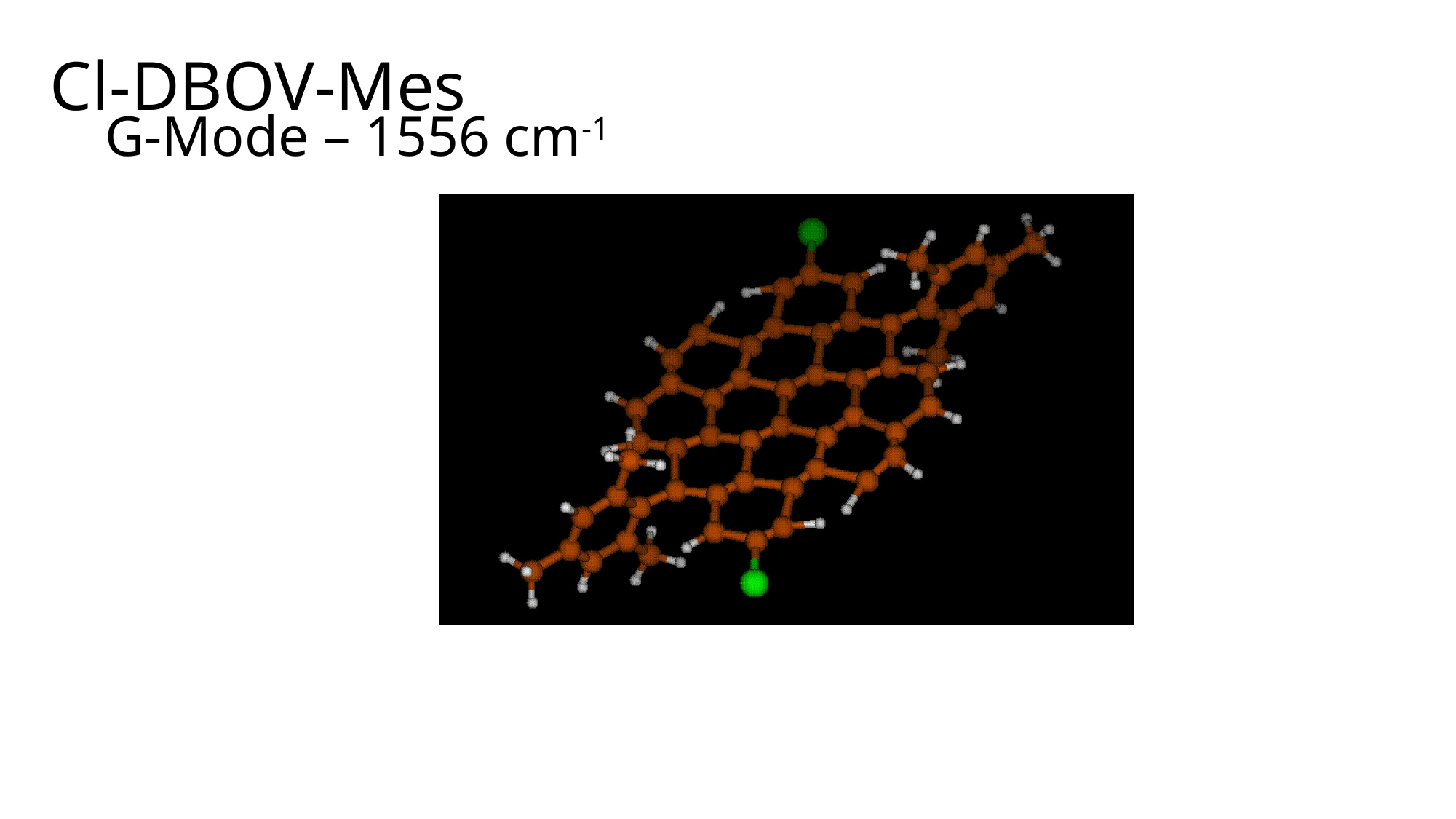

# Cl-DBOV-Mes
G-Mode – 1556 cm-1

## Slide 8
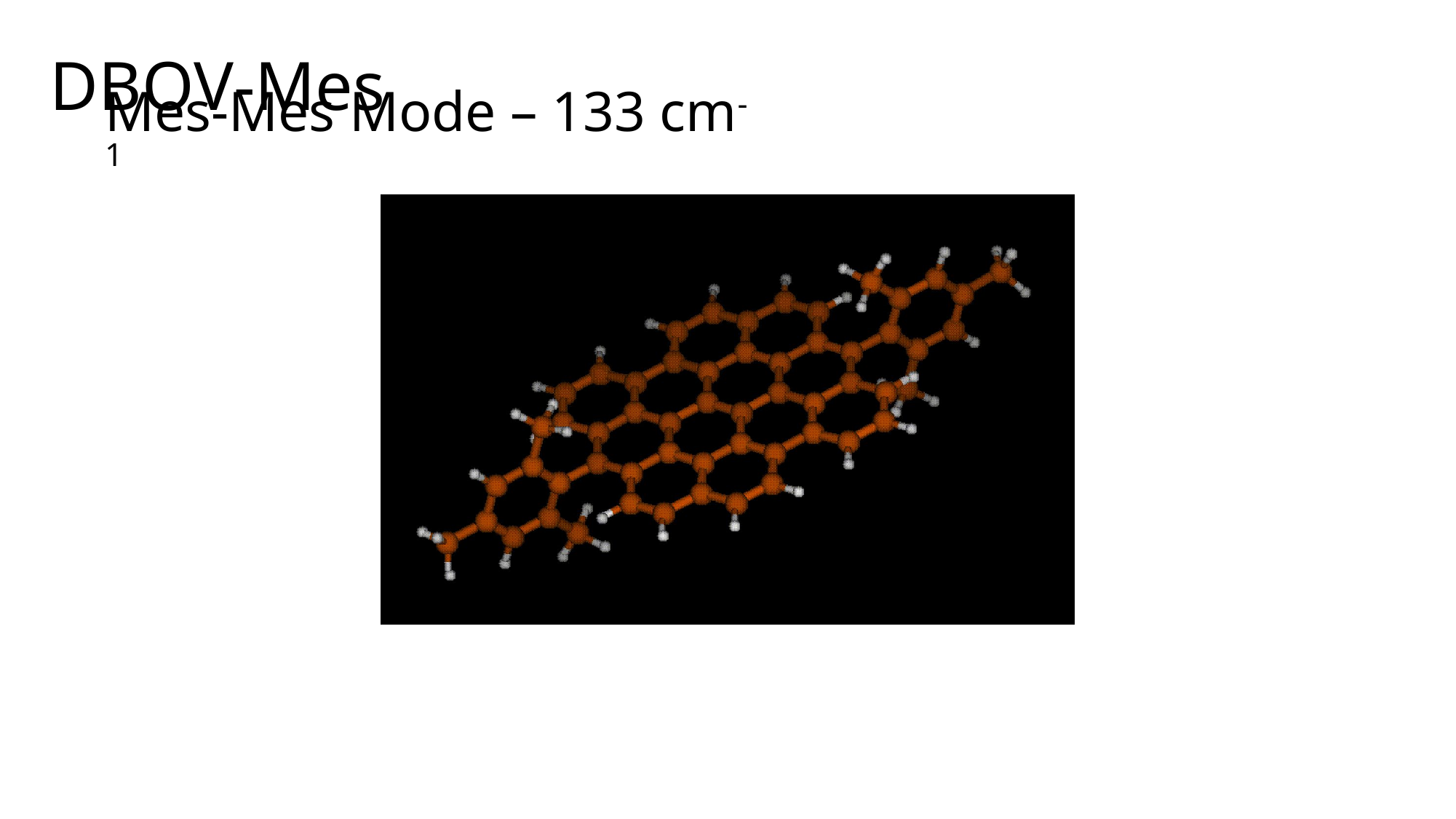

# DBOV-Mes
Mes-Mes Mode – 133 cm-1

## Slide 9
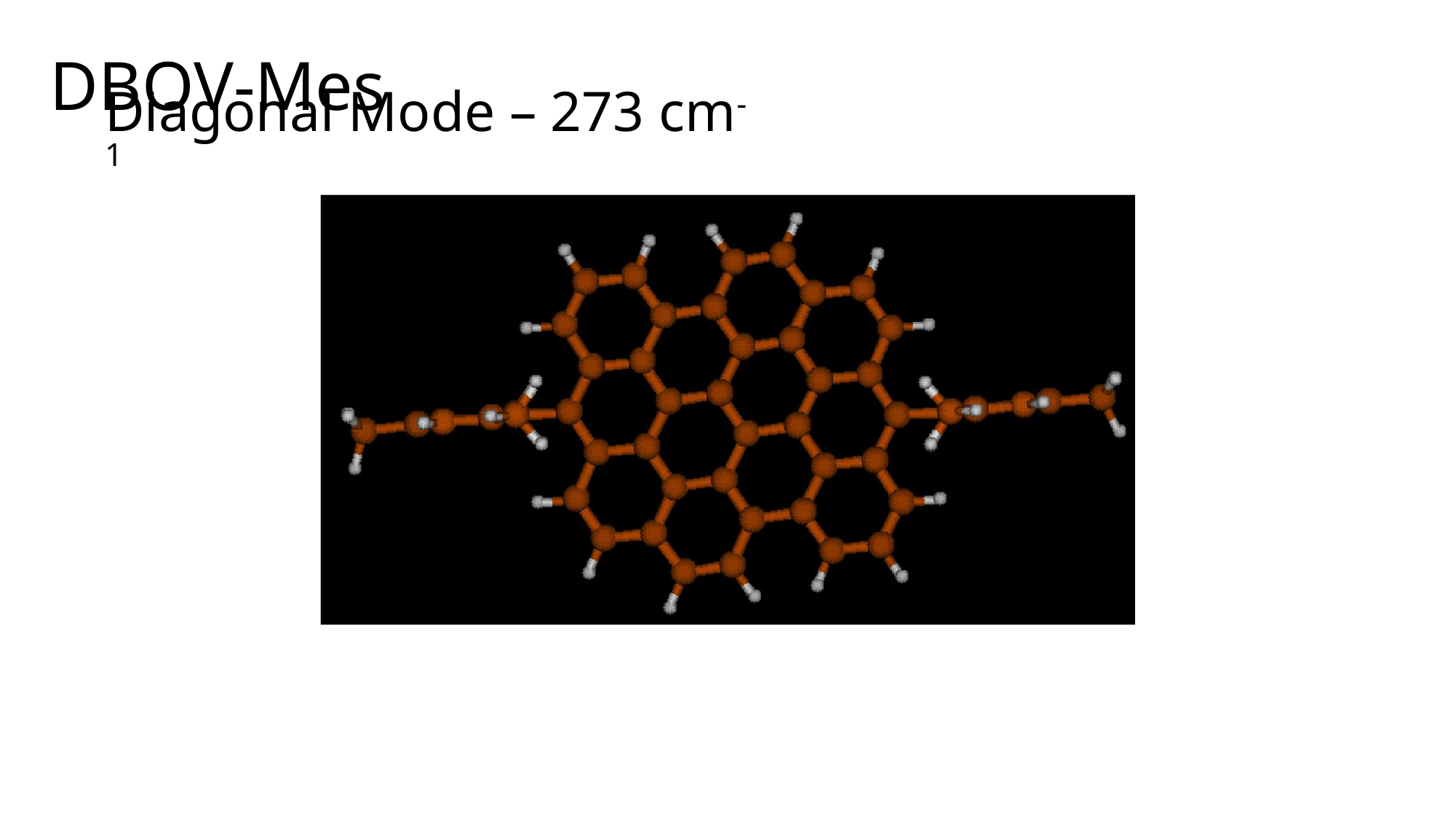

# DBOV-Mes
Diagonal Mode – 273 cm-1

## Slide 10
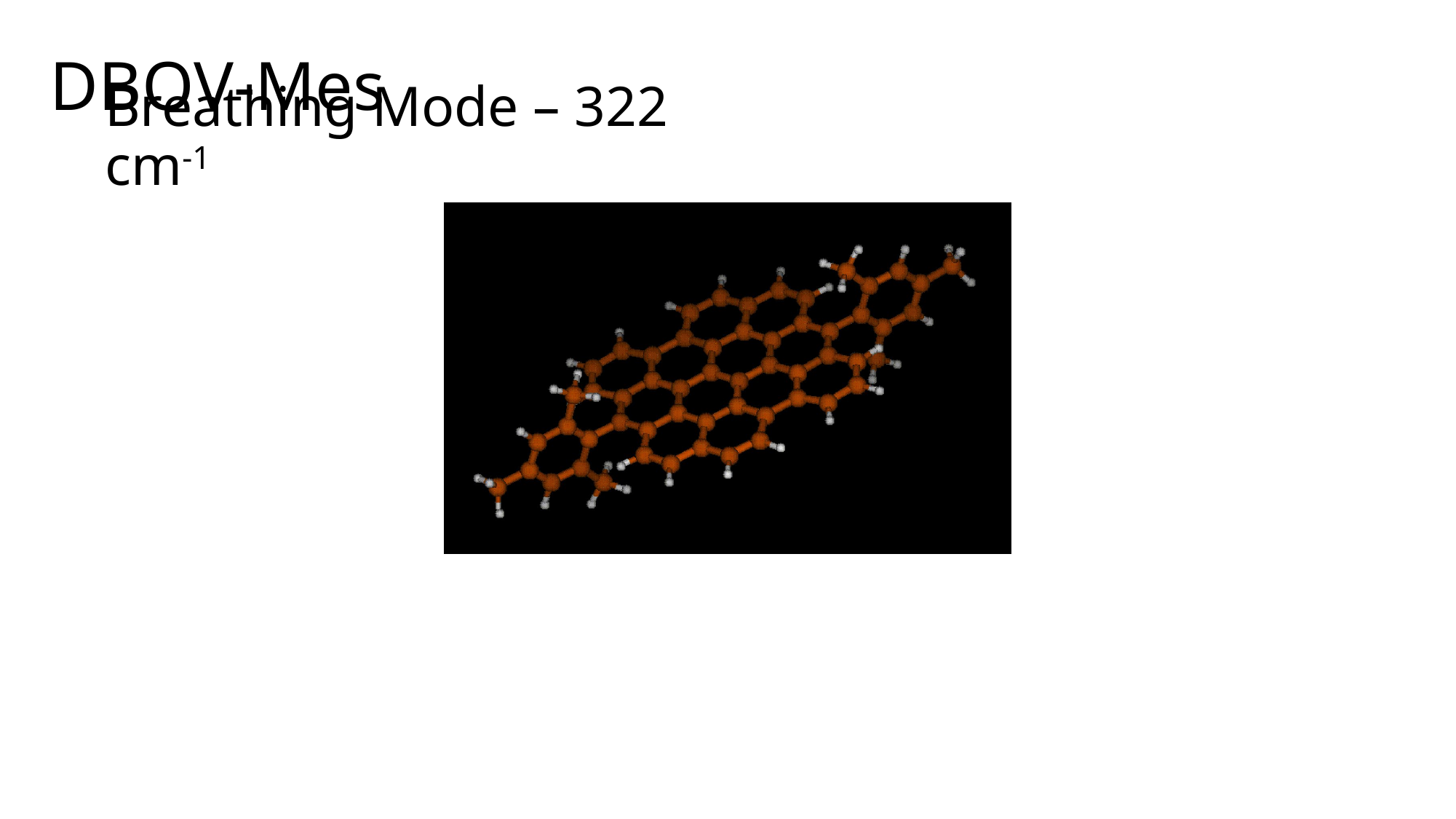

# DBOV-Mes
Breathing Mode – 322 cm-1

## Slide 11
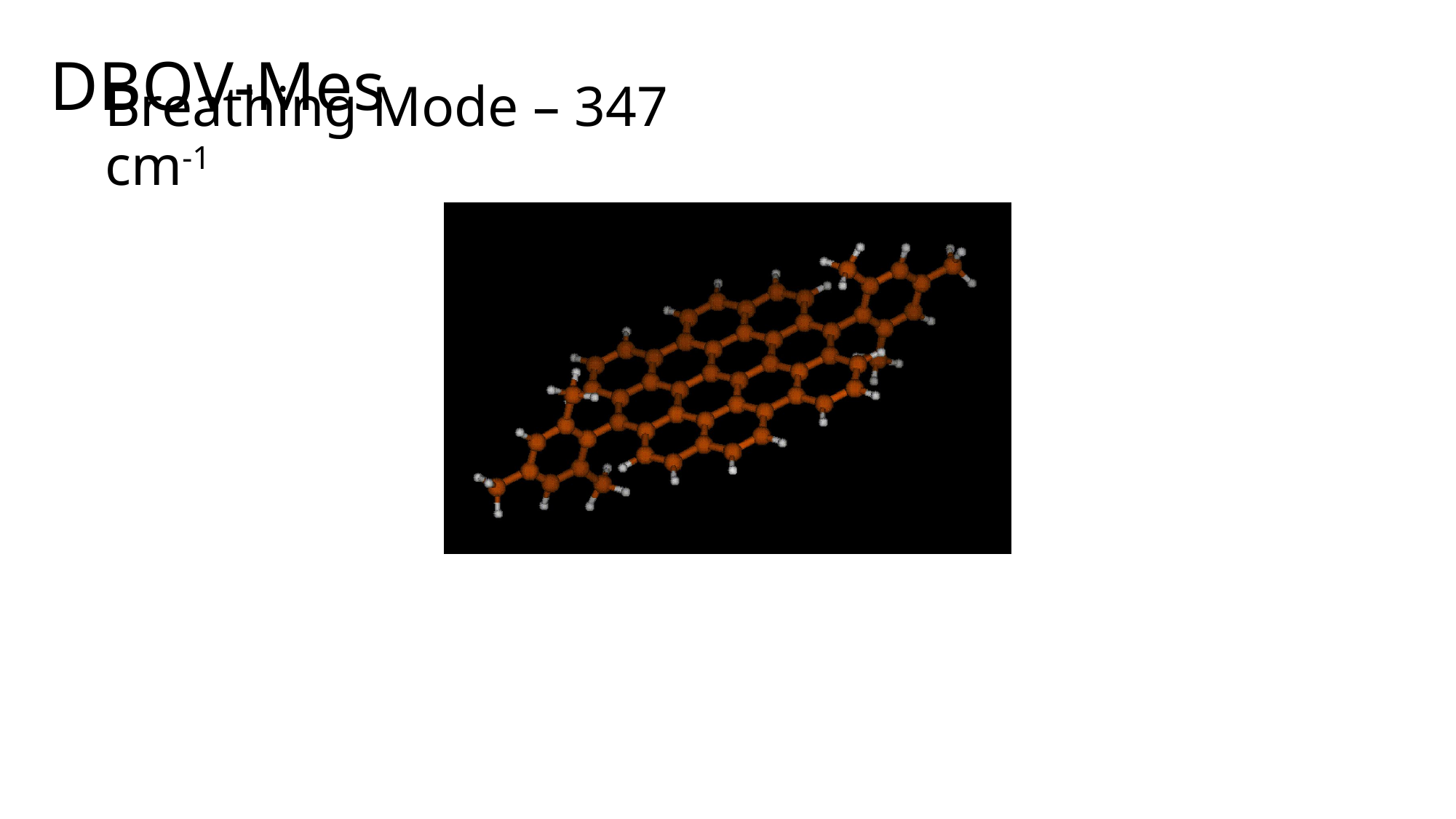

# DBOV-Mes
Breathing Mode – 347 cm-1

## Slide 12
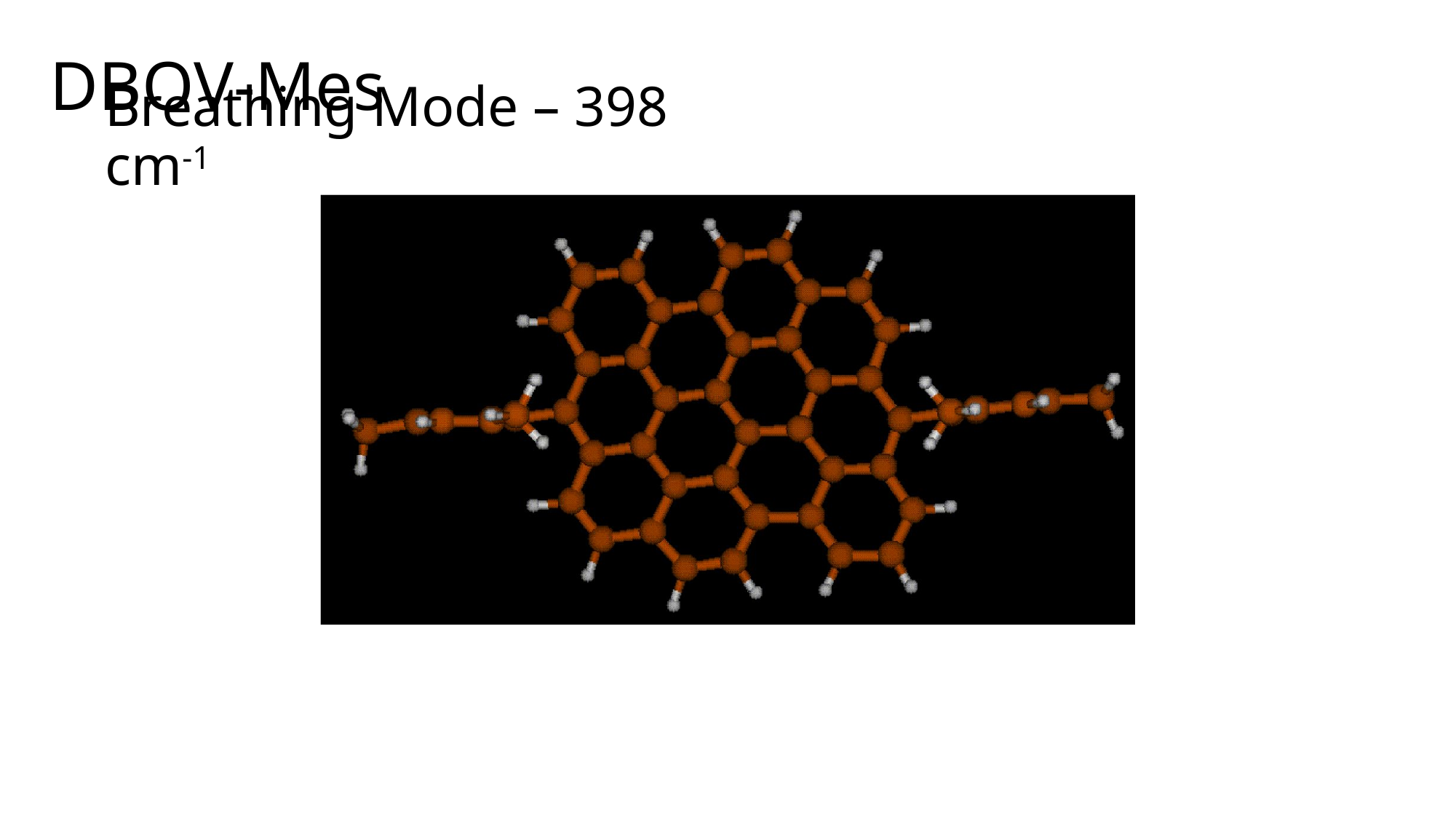

# DBOV-Mes
Breathing Mode – 398 cm-1

## Slide 13
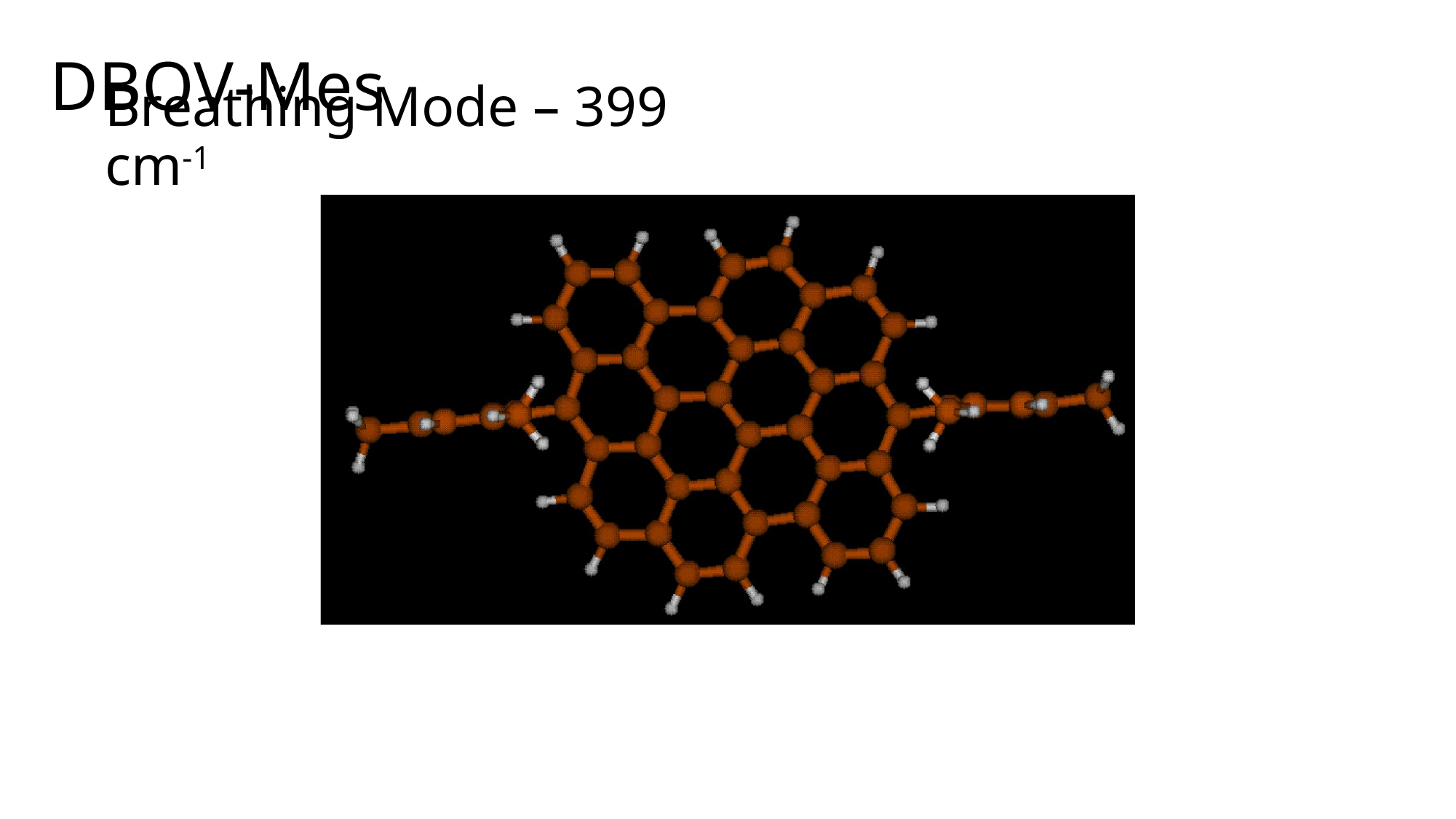

# DBOV-Mes
Breathing Mode – 399 cm-1

## Slide 14
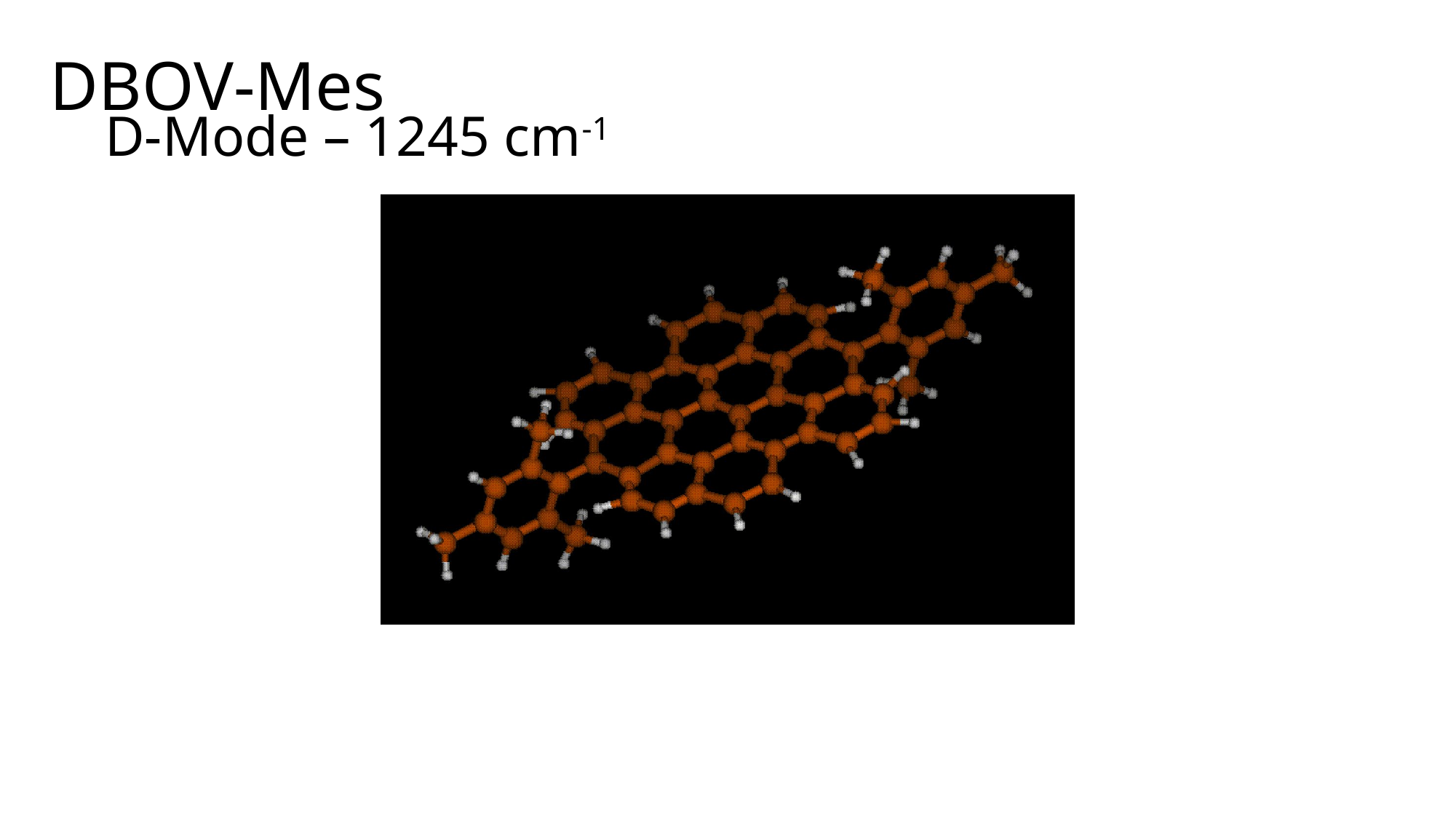

# DBOV-Mes
D-Mode – 1245 cm-1

## Slide 15
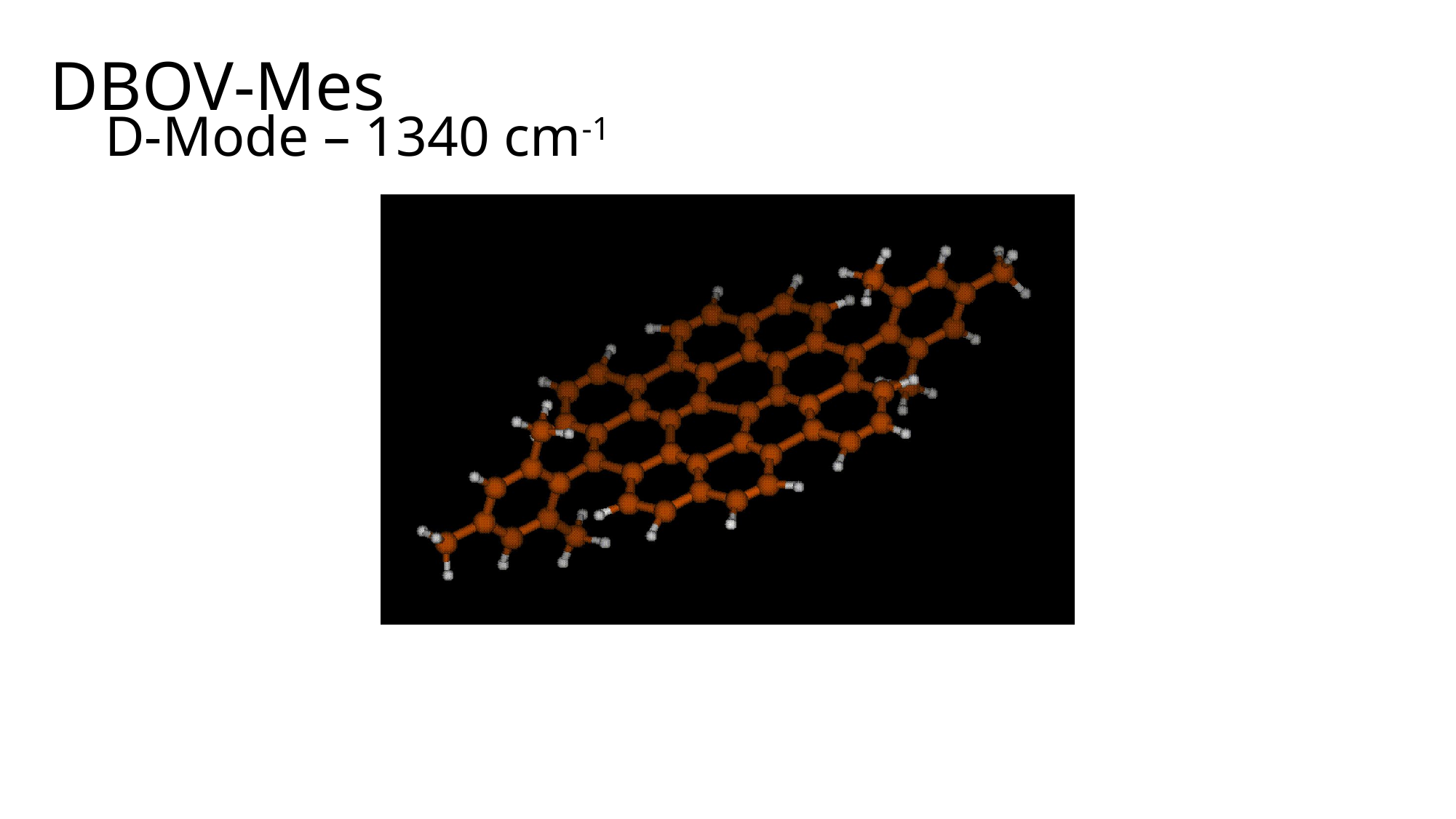

# DBOV-Mes
D-Mode – 1340 cm-1

## Slide 16
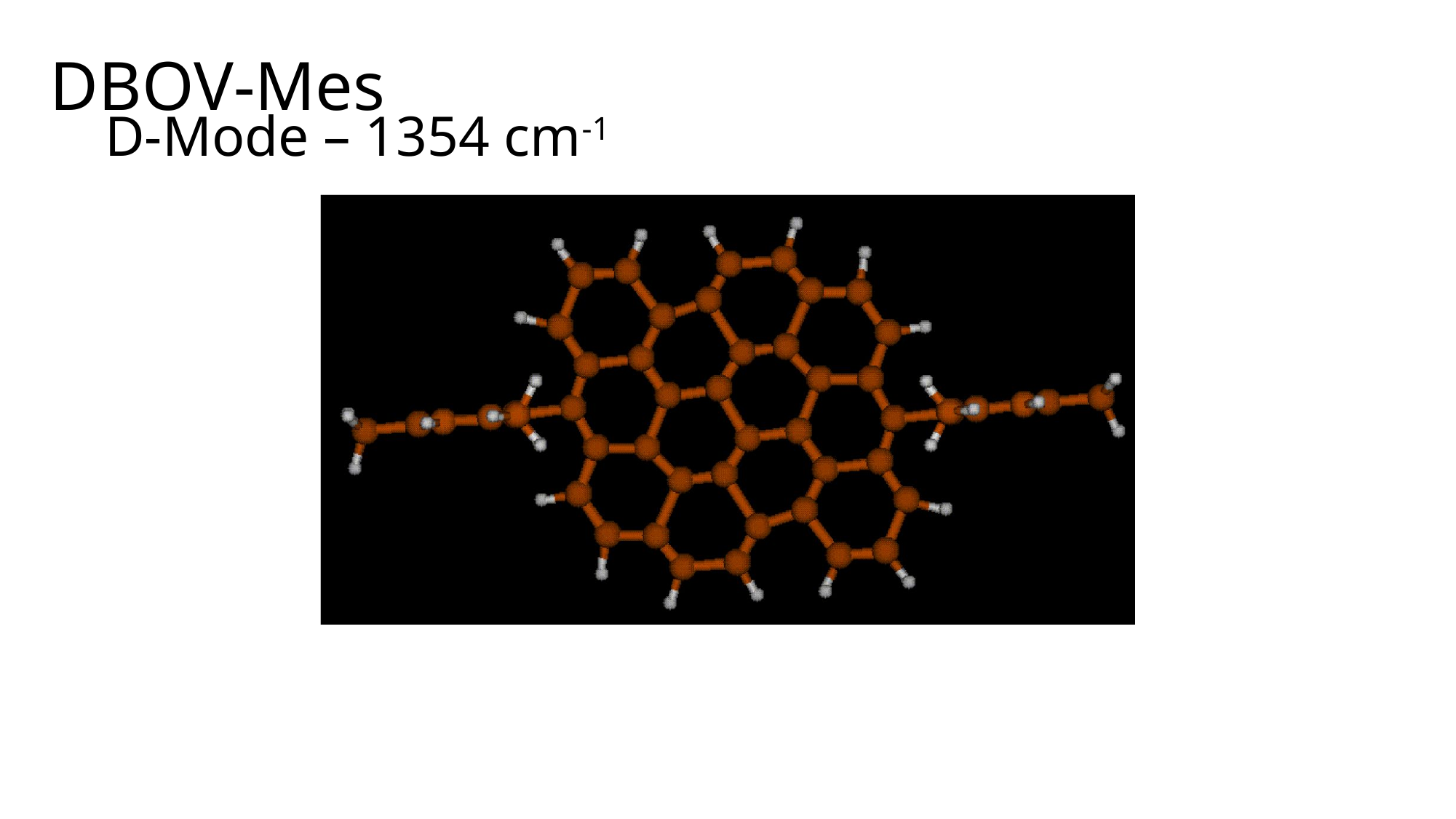

# DBOV-Mes
D-Mode – 1354 cm-1

## Slide 17
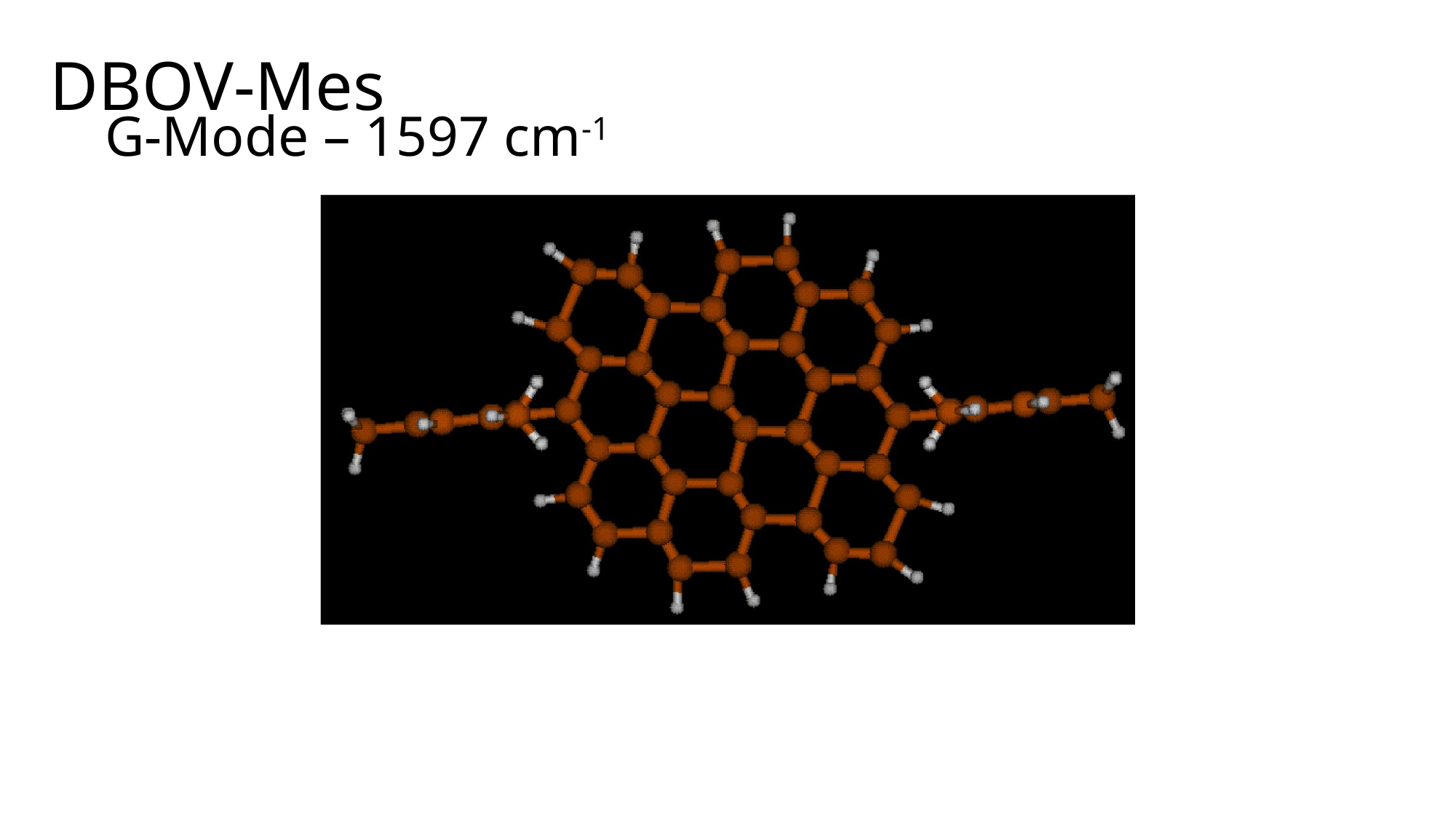

# DBOV-Mes
G-Mode – 1597 cm-1
